# Supplementary material for: Estimating the COVID-19 epidemic trajectory and hospital capacity requirements in South West England: a mathematical modelling framework
Source: BMJ Open. 2021 Jan 7;11(1):e041536. doi: 10.1136/bmjopen-2020-041536 (PMC7797241; doi:10.1136/bmjopen-2020-041536)
Supplement: Supplementary data [file bmjopen-2020-041536supp001.pdf]

# Supplementary information

## "Estimating the COVID-19 epidemic trajectory and hospital capacity requirements in South West England: a mathematical modelling framework"

Ross D. Booton[1], Louis MacGregor[2,3], Lucy Vass[1,2], Katharine J. Looker[2,3], Catherine Hyams[4], Philip D. Bright[5], Irasha Harding[6], Rajeka Lazarus[7], Fergus Hamilton[8], Daniel Lawson[9], Leon Danon[10], Adrian Pratt[11], Richard Wood[12,13], Ellen Brooks-Pollock[1,2,3,†], Katherine M.E. Turner[1,2,3,13,†,*]

*[1]Bristol Veterinary School, University of Bristol, Bristol, UK*

*[2]Population Health Science Institute, Bristol Medical School, University of Bristol, Bristol, UK*

*[3]NIHR Health Protection Research Unit in Behavioural Science and Evaluation*

*[4]Academic Respiratory Unit, Southmead Hospital, University of Bristol, Bristol, UK*

*[5]Immunology, Pathology Sciences, North Bristol NHS Trust, Bristol, UK*

*[6]Consultant in Microbiology, University Hospitals Bristol, Bristol, UK*

*[7]Consultant in Microbiology and Infectious Diseases, University Hospitals Bristol, Bristol, UK*

*[8]Infection Science, Southmead Hospital, North Bristol NHS Trust, Bristol, UK*

*[9]School of Mathematics, University of Bristol, Bristol, UK*

*[10]College of Engineering, Mathematics and Physical Sciences, University of Exeter, Exeter, UK*

*[11]Principal BI Analyst Modelling and Analytics, NHS Bristol, North Somerset and South Gloucestershire CCG*

*[12]Head of Modelling and Analytics, NHS Bristol, North Somerset and South Gloucestershire CCG*

*[13]Health Data Research UK South-West of England Partnership*

*[†]should be considered joint senior author*

**Correspondence: katy.turner@bristol.ac.uk*

| Age group | Population size SW England | Percent population size SW England | Percent population size UK |
|---|---|---|---|
| 0-4 | 296,357 | 5.3% | 6.0% |
| 5-18 | 805,965 | 14.4% | 15.4% |
| 18-29 | 806,885 | 14.4% | 15.4% |
| 30-39 | 654,469 | 11.7% | 13.4% |
| 40-49 | 684,872 | 12.2% | 12.8% |
| 50-59 | 782,317 | 14.0% | 13.4% |
| 60-69 | 671,294 | 12.0% | 10.5% |
| 70+ | 897,576 | 16.0% | 13.1% |

**Table S1:** Demography of SW England compared to the UK.

## *Key assumptions used in the model*

i. *Closed, static population size with no immigration or emigration due to the model being run over a short period of time, and current travel restrictions which should prevent significant movement of individuals in and out of the South West.*

ii. *No nosocomial transmission.*

iii. *Recovered individuals are not susceptible to reinfection within the timeframe of the model horizon.*

iv. *Trajectory of model outputs is assuming that there is no easing of lockdown situation over the timeframe of the model horizon.*

v. *There is no restriction on number of hospital (IC and acute) beds available.*

vi. *Asymptomatic and symptomatic are equally infectious and recover at the same rate.*

vii. *95% reduction in 0-18-year old contacts from school closures*

viii. *Range of 0 - 50% reduction in contacts from social distancing and range of 63 - 90% reduction in contact rates due to lockdown*

ix. *The reduction of any given contact rate is taken to be the minimum from lockdown, school closures and social distancing.*

x. *R_0 is sampled between 2.79 +/- 1.16 and the infectious period and transmission probability are chosen to achieve this*

xi. *Proportion symptomatic which require hospitalisation depends on age, with increased risk for older age groups*

xii. *Proportion in IC who will die depends on age, with increased risk for older age groups*

xiii. *The percentage of infections which become symptomatic is 73.15 - 91.05%*

xiv. *The percentage of those requiring hospital who will require IC 0 - 30%*

xv. *The percentage of those requiring acute beds (but not IC) who will die 5 - 35%*

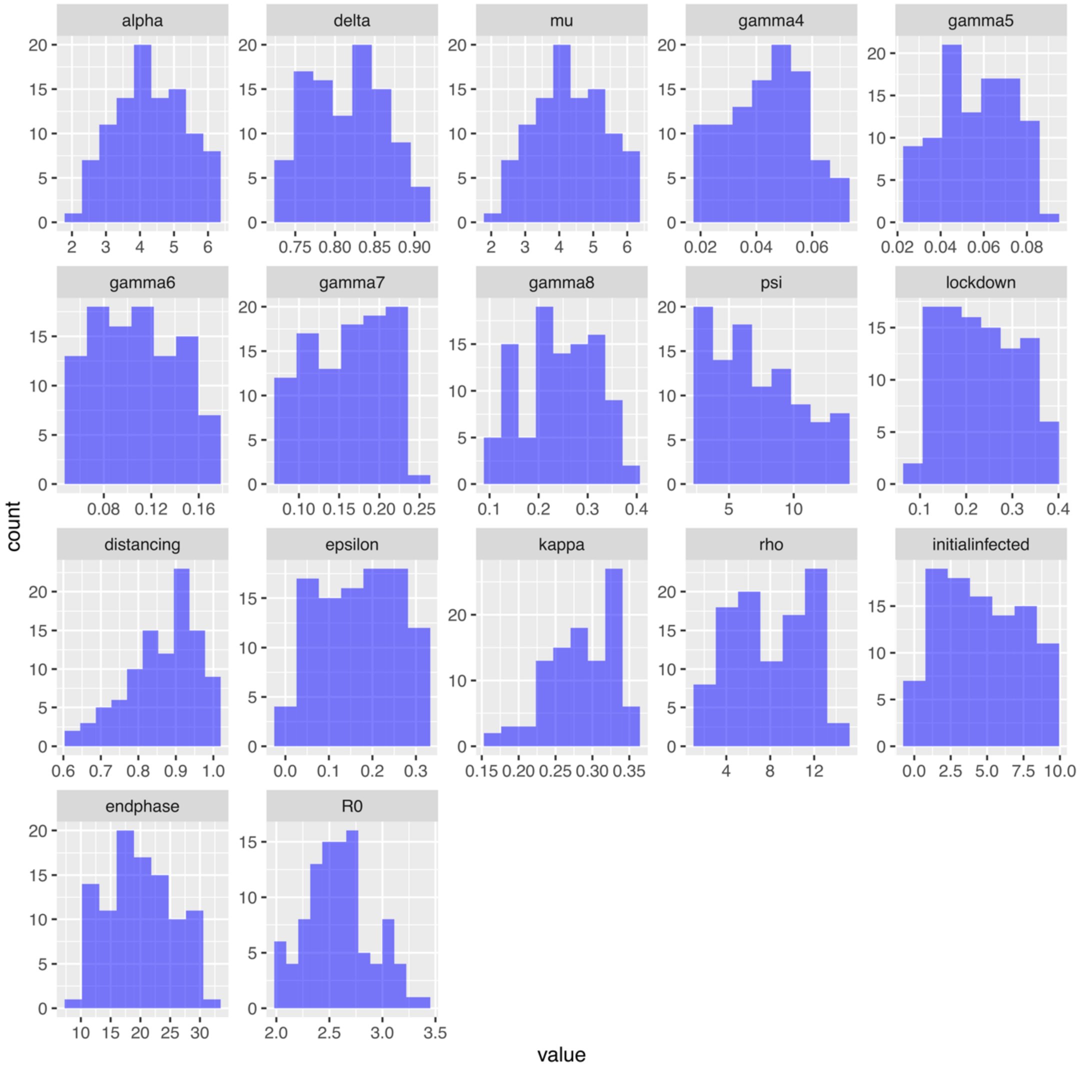


**Figure S1a:** The distribution of the best 100 parameters selected for in the Latin Hypercube Sampling and likelihood fitting from 100,000 simulations.

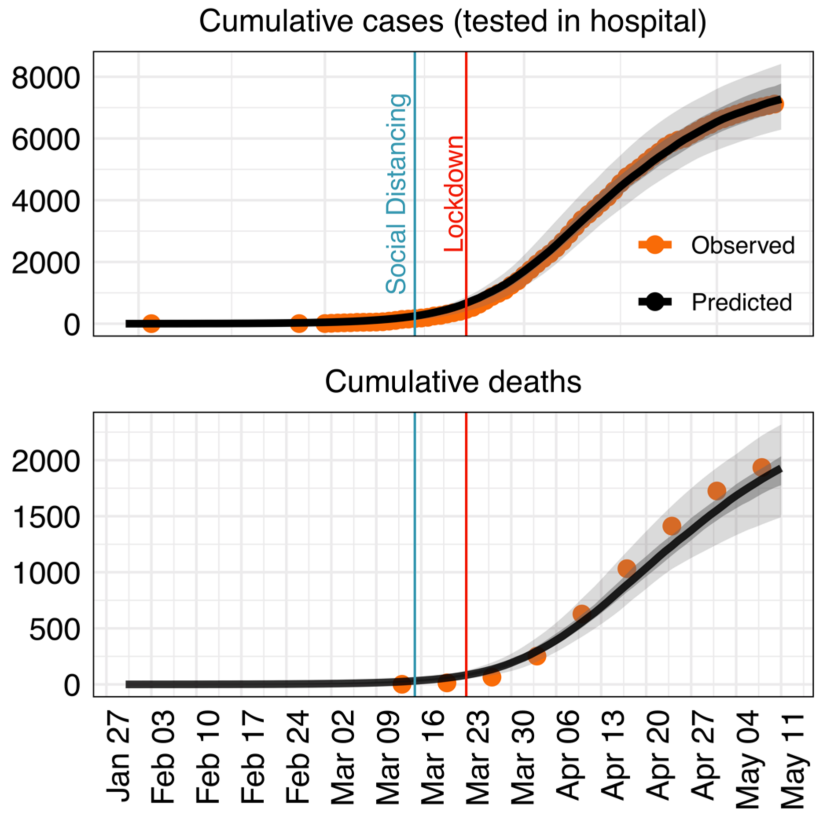


**Figure S2a:** Fitting performance of the model. The cumulative case numbers and hospital deaths for COVID-19 in SW England until lockdown measures were gradually lifted (11th May 2020), based on case data and death data (orange). 95% credible intervals of our model projections are shown in light grey, 50% in dark grey and the median value of the model is highlighted in black. The shaded region indicates the prediction of the model from the data. We did not consider the effects of lockdown being lifted, and our transmission rates are fitted to both before lockdown and after lockdown. Blue and red vertical lines represent the dates when social distancing and school closures/lockdown were introduced nationally, respectively.

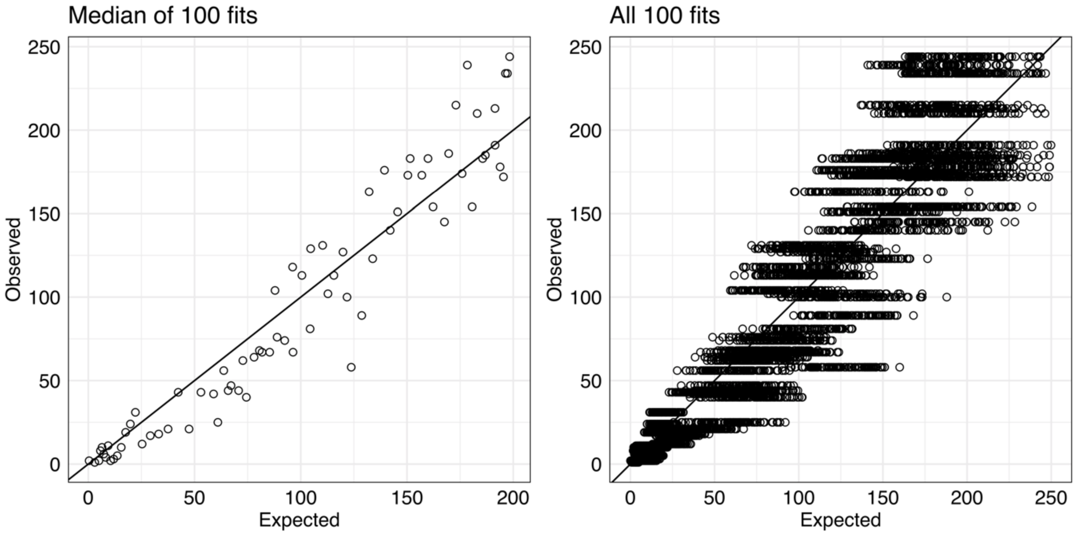


**Figure S2b:** The expected (model) versus observed (data) for the median of 100 fits, and all 100 fits for counts of daily case numbers. The model tends to overestimate the case data at earlier stages of the epidemic and underestimate at later stages of the epidemic.

| | Cumulative Cases | | | | Cumulative Deaths | | | |
|---|---|---|---|---|---|---|---|---|
| Date | Observed | Predicted | Predicted Lower | Predicted Upper | Observed | Predicted | Predicted Lower | Predicted Upper |
| 30/01/2020 | | 1 | 0 | 2 | | 0 | 0 | 0 |
| 31/01/2020 | | 1 | 0 | 3 | | 0 | 0 | 0 |
| 01/02/2020 | | 2 | 0 | 4 | | 0 | 0 | 0 |
| 02/02/2020 | | 2 | 0 | 4 | | 0 | 0 | 0 |
| 03/02/2020 | 2 | 2 | 0 | 5 | | 0 | 0 | 0 |
| 04/02/2020 | | 3 | 0 | 6 | | 0 | 0 | 0 |
| 05/02/2020 | | 3 | 0 | 7 | | 0 | 0 | 1 |
| 06/02/2020 | | 4 | 0 | 8 | | 0 | 0 | 1 |
| 07/02/2020 | | 4 | 1 | 8 | | 0 | 0 | 1 |
| 08/02/2020 | | 4 | 1 | 9 | | 0 | 0 | 1 |
| 09/02/2020 | | 5 | 1 | 10 | | 0 | 0 | 1 |
| 10/02/2020 | | 5 | 1 | 11 | | 1 | 0 | 2 |
| 11/02/2020 | | 6 | 1 | 12 | | 1 | 0 | 2 |
| 12/02/2020 | | 6 | 1 | 13 | | 1 | 0 | 2 |
| 13/02/2020 | | 7 | 1 | 15 | | 1 | 0 | 2 |

| | | | | | | | | |
|---|---|---|---|---|---|---|---|---|
| 14/02/2020 | | 8 | 1 | 16 | | 1 | 0 | 2 |
| 15/02/2020 | | 9 | 1 | 17 | | 1 | 0 | 3 |
| 16/02/2020 | | 10 | 2 | 19 | | 1 | 0 | 3 |
| 17/02/2020 | | 11 | 2 | 21 | | 1 | 0 | 3 |
| 18/02/2020 | | 12 | 2 | 23 | | 2 | 0 | 4 |
| 19/02/2020 | | 13 | 3 | 25 | | 2 | 0 | 4 |
| 20/02/2020 | | 14 | 3 | 27 | | 2 | 0 | 4 |
| 21/02/2020 | | 16 | 3 | 30 | | 2 | 0 | 5 |
| 22/02/2020 | | 18 | 4 | 33 | | 2 | 1 | 5 |
| 23/02/2020 | | 20 | 5 | 37 | | 3 | 1 | 6 |
| 24/02/2020 | | 23 | 5 | 40 | | 3 | 1 | 6 |
| 25/02/2020 | | 25 | 6 | 45 | | 3 | 1 | 7 |
| 26/02/2020 | 3 | 28 | 7 | 49 | | 4 | 1 | 8 |
| 27/02/2020 | | 32 | 9 | 55 | | 4 | 1 | 9 |
| 28/02/2020 | | 36 | 10 | 61 | | 5 | 1 | 9 |
| 29/02/2020 | | 40 | 12 | 67 | | 5 | 1 | 10 |
| 01/03/2020 | 5 | 45 | 14 | 75 | | 6 | 2 | 12 |
| 02/03/2020 | 13 | 50 | 16 | 83 | | 7 | 2 | 13 |
| 03/03/2020 | 23 | 57 | 19 | 93 | | 8 | 2 | 14 |
| 04/03/2020 | 29 | 64 | 22 | 104 | | 8 | 3 | 16 |
| 05/03/2020 | 33 | 72 | 26 | 116 | | 10 | 3 | 17 |
| 06/03/2020 | 44 | 82 | 30 | 129 | | 11 | 4 | 19 |
| 07/03/2020 | 46 | 92 | 35 | 144 | | 12 | 4 | 21 |
| 08/03/2020 | 49 | 105 | 41 | 159 | | 14 | 5 | 24 |
| 09/03/2020 | 54 | 118 | 47 | 177 | | 15 | 6 | 26 |
| 10/03/2020 | 64 | 134 | 55 | 196 | | 17 | 7 | 29 |
| 11/03/2020 | 83 | 151 | 65 | 218 | | 19 | 8 | 32 |
| 12/03/2020 | 107 | 171 | 76 | 243 | | 21 | 9 | 36 |
| 13/03/2020 | 138 | 194 | 88 | 271 | 1 | 24 | 11 | 40 |
| 14/03/2020 | 150 | 219 | 103 | 303 | | 28 | 13 | 44 |
| 15/03/2020 | 167 | 248 | 119 | 339 | | 31 | 15 | 49 |
| 16/03/2020 | 185 | 281 | 138 | 382 | | 35 | 18 | 55 |
| 17/03/2020 | 206 | 320 | 161 | 432 | | 40 | 21 | 62 |
| 18/03/2020 | 249 | 364 | 187 | 488 | | 45 | 25 | 69 |
| 19/03/2020 | 270 | 410 | 220 | 551 | | 51 | 28 | 77 |

| | | | | | | | | |
|---|---|---|---|---|---|---|---|---|
| 20/03/2020 | 313 | 461 | 258 | 618 | 13 | 58 | 33 | 86 |
| 21/03/2020 | 355 | 521 | 301 | 693 | | 65 | 38 | 97 |
| 22/03/2020 | 399 | 589 | 351 | 776 | | 74 | 43 | 110 |
| 23/03/2020 | 461 | 669 | 403 | 868 | | 83 | 50 | 124 |
| 24/03/2020 | 529 | 754 | 458 | 969 | | 94 | 57 | 139 |
| 25/03/2020 | 633 | 840 | 519 | 1085 | | 106 | 64 | 155 |
| 26/03/2020 | 751 | 946 | 588 | 1216 | | 119 | 73 | 174 |
| 27/03/2020 | 880 | 1045 | 670 | 1354 | 65 | 134 | 82 | 195 |
| 28/03/2020 | 982 | 1154 | 761 | 1499 | | 152 | 93 | 218 |
| 29/03/2020 | 1082 | 1271 | 860 | 1648 | | 170 | 105 | 244 |
| 30/03/2020 | 1245 | 1406 | 967 | 1824 | | 192 | 119 | 273 |
| 31/03/2020 | 1385 | 1552 | 1078 | 2010 | | 216 | 135 | 305 |
| 01/04/2020 | 1568 | 1702 | 1201 | 2220 | | 240 | 155 | 340 |
| 02/04/2020 | 1751 | 1861 | 1331 | 2435 | | 266 | 177 | 378 |
| 03/04/2020 | 1937 | 2025 | 1467 | 2655 | 253 | 297 | 203 | 417 |
| 04/04/2020 | 2111 | 2193 | 1612 | 2881 | | 327 | 231 | 458 |
| 05/04/2020 | 2265 | 2365 | 1772 | 3107 | | 360 | 260 | 503 |
| 06/04/2020 | 2448 | 2552 | 1940 | 3333 | | 394 | 290 | 550 |
| 07/04/2020 | 2661 | 2747 | 2121 | 3556 | | 431 | 322 | 599 |
| 08/04/2020 | 2895 | 2941 | 2312 | 3782 | | 472 | 356 | 652 |
| 09/04/2020 | 3139 | 3134 | 2491 | 4006 | | 512 | 390 | 707 |
| 10/04/2020 | 3373 | 3327 | 2674 | 4226 | 628 | 554 | 424 | 765 |
| 11/04/2020 | 3545 | 3517 | 2858 | 4442 | | 598 | 461 | 820 |
| 12/04/2020 | 3723 | 3710 | 3044 | 4636 | | 641 | 498 | 876 |
| 13/04/2020 | 3914 | 3891 | 3227 | 4852 | | 689 | 538 | 936 |
| 14/04/2020 | 4099 | 4075 | 3392 | 5065 | | 738 | 578 | 998 |
| 15/04/2020 | 4309 | 4262 | 3553 | 5258 | | 789 | 620 | 1061 |
| 16/04/2020 | 4548 | 4446 | 3710 | 5454 | | 840 | 665 | 1123 |
| 17/04/2020 | 4763 | 4614 | 3866 | 5652 | 1032 | 889 | 714 | 1184 |
| 18/04/2020 | 4908 | 4779 | 4026 | 5841 | | 938 | 762 | 1245 |
| 19/04/2020 | 5062 | 4930 | 4184 | 6021 | | 988 | 807 | 1305 |
| 20/04/2020 | 5235 | 5090 | 4338 | 6193 | | 1039 | 852 | 1365 |
| 21/04/2020 | 5408 | 5256 | 4480 | 6356 | | 1089 | 900 | 1424 |
| 22/04/2020 | 5559 | 5408 | 4614 | 6510 | | 1141 | 946 | 1481 |
| 23/04/2020 | 5735 | 5548 | 4746 | 6657 | | 1190 | 989 | 1538 |

| | | | | | | | | |
|---|---|---|---|---|---|---|---|---|
| 24/04/2020 | 5858 | 5689 | 4876 | 6796 | 1412 | 1234 | 1029 | 1593 |
| 25/04/2020 | 5947 | 5839 | 5001 | 6927 | | 1284 | 1063 | 1647 |
| 26/04/2020 | 6005 | 5971 | 5122 | 7051 | | 1327 | 1096 | 1699 |
| 27/04/2020 | 6132 | 6085 | 5239 | 7169 | | 1373 | 1127 | 1749 |
| 28/04/2020 | 6245 | 6203 | 5353 | 7279 | | 1421 | 1158 | 1797 |
| 29/04/2020 | 6376 | 6317 | 5450 | 7384 | | 1467 | 1187 | 1844 |
| 30/04/2020 | 6457 | 6428 | 5539 | 7496 | | 1512 | 1217 | 1889 |
| 01/05/2020 | 6570 | 6539 | 5624 | 7607 | 1725 | 1554 | 1247 | 1933 |
| 02/05/2020 | 6637 | 6638 | 5704 | 7706 | | 1598 | 1276 | 1974 |
| 03/05/2020 | 6711 | 6722 | 5780 | 7795 | | 1643 | 1304 | 2014 |
| 04/05/2020 | 6787 | 6801 | 5852 | 7880 | | 1683 | 1331 | 2053 |
| 05/05/2020 | 6854 | 6869 | 5923 | 7960 | | 1721 | 1357 | 2096 |
| 06/05/2020 | 6921 | 6947 | 5992 | 8038 | | 1759 | 1383 | 2139 |
| 07/05/2020 | 6985 | 7010 | 6057 | 8113 | | 1794 | 1403 | 2180 |
| 08/05/2020 | 7025 | 7090 | 6119 | 8193 | 1935 | 1830 | 1427 | 2219 |
| 09/05/2020 | 7069 | 7160 | 6178 | 8268 | | 1864 | 1449 | 2253 |
| 10/05/2020 | 7116 | 7218 | 6235 | 8341 | | 1897 | 1470 | 2286 |
| 11/05/2020 | 7172 | 7273 | 6288 | 8415 | | 1928 | 1491 | 2317 |

**Table S2:** The predicted median and 95% credible interval for cumulative cases and deaths alongside the observed cumulative cases and deaths.

## Model performance

Here we evaluate the model performance as a tool to forecast the potential cumulative cases and deaths related to COVID-19. We do this by using only a certain percentage of the total available data until a) 27th April 2020, b) 20th April 2020, c) 13th April 2020 and d) 6th April 2020) to fit the model, then comparing these projections to the actual data observed past each of these dates. This gives an interpretable way to estimate how well the forecast generated by the model could be expected to perform.

Figures S3a-d show the overall performance of the model, which in general overestimates the total cumulative cases, but captures the cumulative deaths more accurately. All final estimates from the data are within the 95% expected credible interval predictions regardless of the date chosen to fit the model to.

Figure S3a shows the model prediction using data only up to 27th April 2020, which is based on 82% of the available data. This the most reliable prediction, while the most variable predictions come from those which use 53% of the available data (Figure S3d), suggesting that the model can accurately forecast the immediate future with higher reliability – while further time points are harder to predict (although the model predictions based on only 53% of the data also perform reasonably well and the final actuals are within the predicted 95% credible interval).

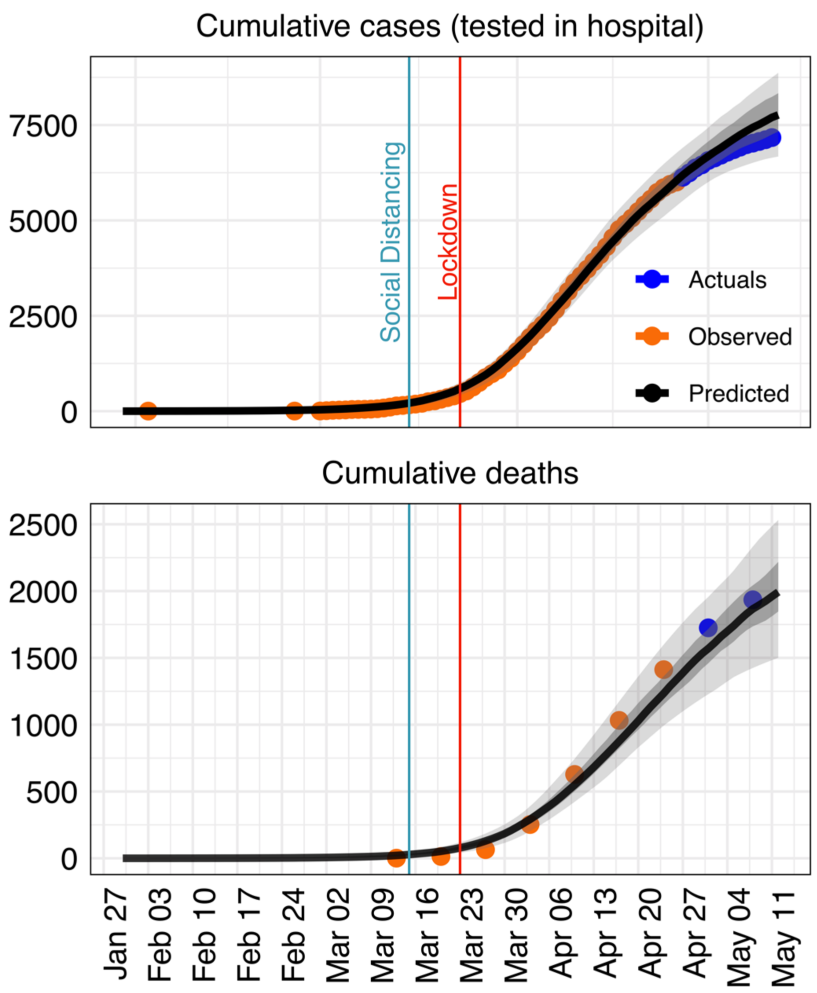


**Figure S3a:** Fitting performance of the model using only the data until the 27th April 2020 (which represents 82% of the available data points). 95% credible intervals of our model projections are shown in light grey, 50% in dark grey and the median value of the model is highlighted in black. The shaded region indicates the prediction of the model from the data. Blue and red vertical lines represent the dates when social distancing and school closures/lockdown were introduced nationally, respectively. 'Actuals' represent the points not included in the fitting, while observed data are used to fit the model.

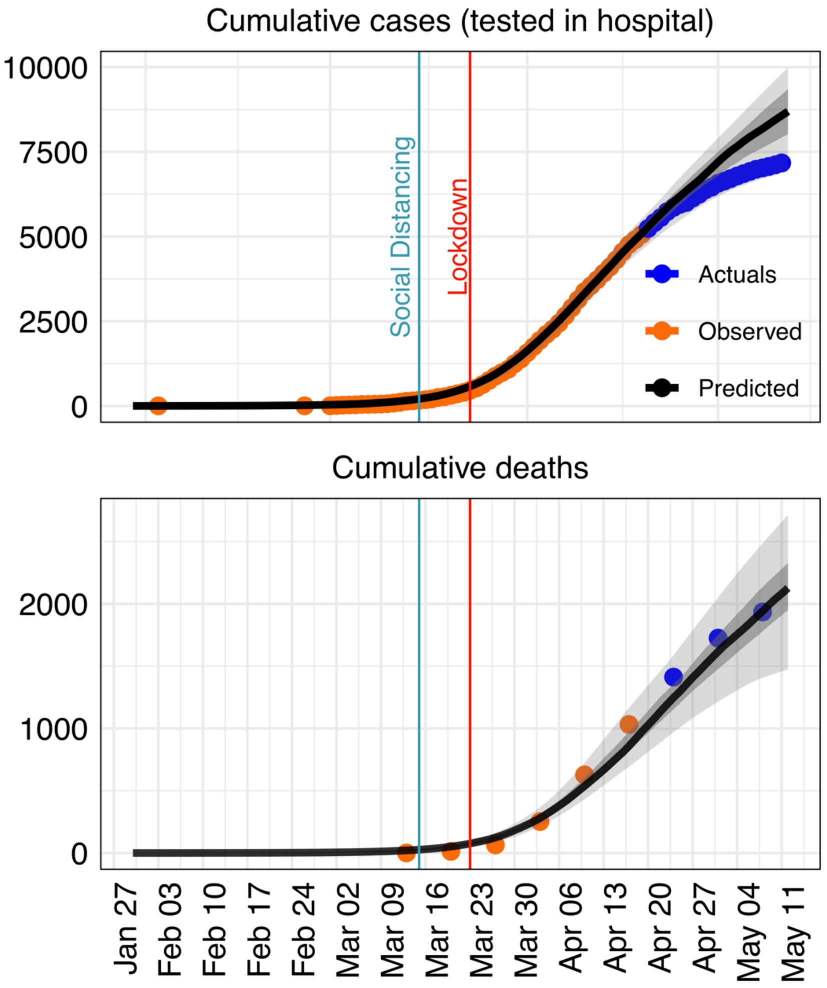


**Figure S3b** Fitting performance of the model using only the data until the 20th April 2020 (which represents 72% of the available data points).

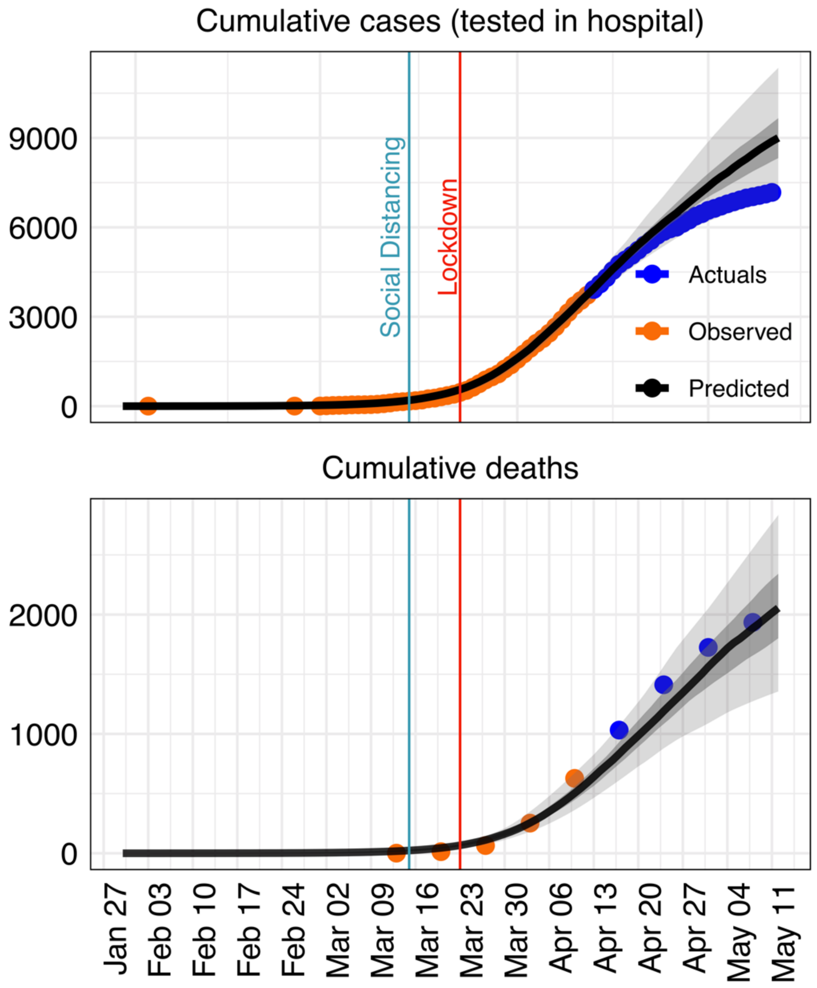


119

120 **Figure S3c:** Fitting performance of the model using only the data until the 13th April 2020 (which
121 represents 62% of the available data points).

122

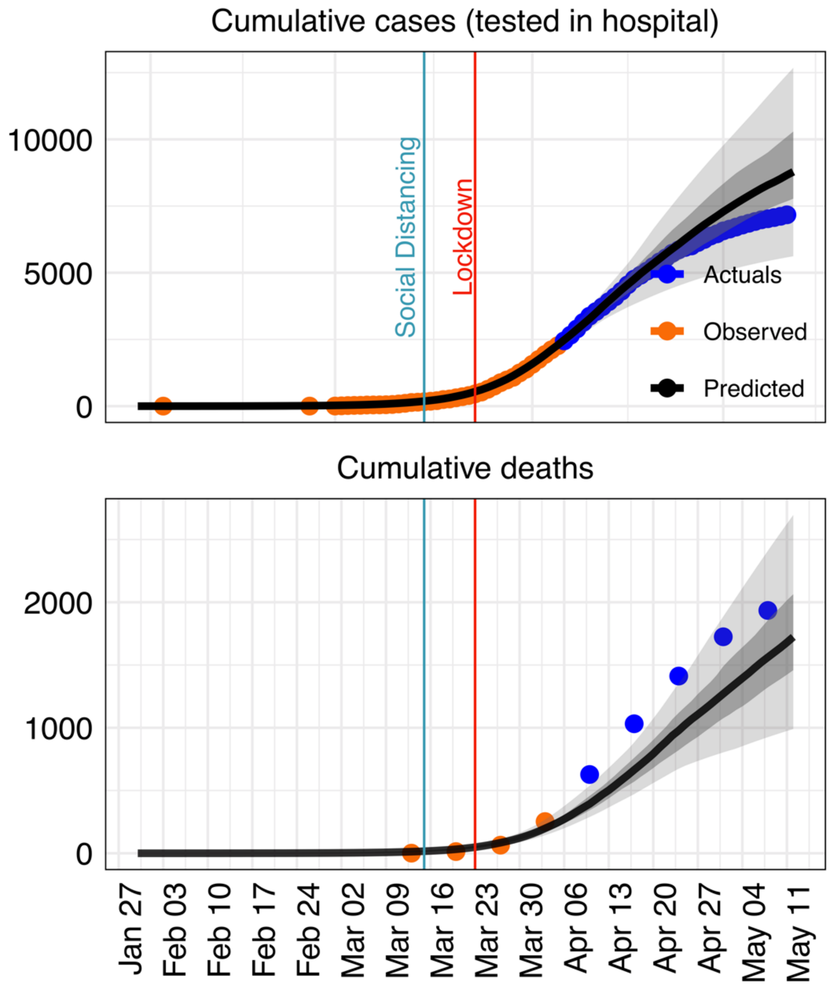


**Figure S3d:** Fitting performance of the model using only the data until the 6th April 2020 (which represents 53% of the available data points).

BMJ Publishing Group Limited (BMJ) disclaims all liability and responsibility arising from any reliance placed on this supplemental material which has been supplied by the author(s)


## Sensitivity analysis

The choice of the final sample size *m*=100 taken from 100k samples is based on a bias-variance trade-off with lower m giving a better model fit but higher *m* allowing more predictions to be included. The choice of *m* is not of practical significance for the median and 95% credible intervals of the main outcome measures of the model – bed modelling - as shown in the below table.

| | **Median (95% CrI) value on 11th May 2020** | | | |
|---|---|---|---|---|
| **Size of final sample size from 100k samples** | *Total recovered* | *Total infectious* | *Total in acute beds* | *Total in IC beds* |
| 50 | 191k (143k – 276k) | 5k (2k – 10k) | 579 (152 – 1424) | 107 (14 – 497) |
| 100 | 189k (142k – 278k) | 6k (2k – 12k) | 701 (169 – 1543) | 110 (8 – 464) |
| 200 | 189k (141k - 276k) | 6k (2k – 16k) | 709 (171 – 1564) | 105 (5 – 454) |
| 500 | 191k (132k – 289k) | 7k (2k – 20k) | 690 (171 – 1644) | 109 (5 – 426) |

Increasing *m* does slightly increase the estimate of the infectious population (*total infectious)*. This is explained by including models with slightly higher *R* value, i.e. *R* is biased upwards as we move further away from the best answer. The bias is small compared to the modelling uncertainty.

| | **Median (95% CrI) of parameter** | | | | | | | |
|---|---|---|---|---|---|---|---|---|
| **Size of final sample size from 100k samples** | *R* | $\delta$ | $1/\mu$ | $1/\rho$ | $1/\psi$ | $\epsilon$ | $\kappa$ | *endphase* |
| 50 | 2.5 (2.1 - 3.1) | 82 (74 – 90%) | 3.9 (2.6 – 5.8) | 6.3 (2.4 – 13.1) | 6.5 (2.6 – 13.8) | 19 (4 – 28%) | 29 (19 – 34%) | 21.3 (11.1 – 30.0) |
| 100 | 2.6 (2.1 - 3.2) | 82 (74 – 90%) | 4.3 (2.5 – 6.0) | 7.7 (2.3 – 13.3) | 6.7 (2.5 – 13.8) | 16 (2 – 30%) | 28 (19 – 34% | 19.7 (10.8 – 30.0) |
| 200 | 2.6 (2.1 - 3.3) | 82 (74 – 91%) | 4.5 (2.4 – 6.7) | 7.8 (2.3 – 13.6) | 6.7 (2.4 – 13.8) | 16 (1 – 30%) | 28 (17 – 34%) | 18.5 (8.6 – 30.3) |
| 500 | 2.7 (2.1 - 3.5) | 82 (74 – 90%) | 5.0 (2.5 – 8.3) | 7.3 (2.4 – 13.6) | 7.2 (2.5 – 13.8) | 17 (1 – 29%) | 28 (15 – 35%) | 18.3 (6.4 – 30.3) |

The inverse of $\mu$ and $\psi$ becomes bigger (as it is related to the selection of R), as the sample size becomes larger. This then reduces the parameters *endphase*, $\kappa$, and $\epsilon$ in order to account for this bias in R.

We choose to report values for *m*=100 samples as part of the bias-variance trade-off. The bias is small for this choice, with R close to unbiased and the confidence intervals capture all of the significant variation components. The qualitative inferences would not change with any of the above choices of *m* and uncertainty has been well captured.
