# Supplementary material for: Estimating the COVID-19 epidemic trajectory and hospital capacity requirements in South West England: a mathematical modelling framework
Source: BMJ Open. 2021 Jan 7;11(1):e041536. doi: 10.1136/bmjopen-2020-041536 (PMC7797241; doi:10.1136/bmjopen-2020-041536)
Supplement: Author's manuscript [file bmjopen-2020-041536.draft_revisions.pdf]

BMJ Open is committed to open peer review. As part of this commitment we make the peer review history of every article we publish publicly available.

When an article is published we post the peer reviewers' comments and the authors' responses online. We also post the versions of the paper that were used during peer review. These are the versions that the peer review comments apply to.

The versions of the paper that follow are the versions that were submitted during the peer review process. They are not the versions of record or the final published versions. They should not be cited or distributed as the published version of this manuscript.

BMJ Open is an open access journal and the full, final, typeset and author-corrected version of record of the manuscript is available on our site with no access controls, subscription charges or pay-per-view fees (http://bmjopen.bmj.com).

If you have any questions on BMJ Open's open peer review process please email info.bmjopen@bmj.com

BMJ Open

Estimating the COVID-19 epidemic trajectory and hospital capacity requirements in South West England: a mathematical modelling framework

Journal:	*BMJ Open*
Manuscript ID	bmjopen-2020-041536
Article Type:	Original research
Date Submitted by the Author:	10-Jun-2020

Complete List of Authors:	Booton, Ross; University of Bristol School of Veterinary Sciences<br>MacGregor, Louis; Bristol Medical School<br>Vass, Lucy; University of Bristol School of Veterinary Sciences<br>Looker, Katharine; Bristol Medical School<br>Hyams, Catherine; Academic Respiratory Unit, Southmead Hospital<br>Bright, Philip; Immunology, Pathology Sciences, North Bristol NHS Trust<br>Harding, Irasha; Consultant in Microbiology, University Hospitals Bristol<br>Lazarus, Rajeka; Consultant in Microbiology and Infectious Diseases, University Hospitals Bristol<br>Hamilton, Fergus; Infection Science, Southmead Hospital, North Bristol NHS Trust<br>Lawson, Daniel; University of Bristol School of Mathematics<br>Danon, Leon; University of Exeter College of Engineering Mathematics and Physical Sciences<br>Pratt, Adrian; Head of Modelling and Analytics, NHS Bristol, North Somerset and South Gloucestershire CCG<br>Wood, Richard; Principal BI Analyst Modelling and Analytics, NHS Bristol, North Somerset and South Gloucestershire CCG<br>Brooks-Pollock, Ellen; University of Bristol School of Veterinary Sciences; Bristol Medical School<br>Turner, Katy; University of Bristol School of Veterinary Sciences; Health Data Research UK South-West of England Partnership, Population Health Science Institute

Keywords:	EPIDEMIOLOGY, Public health < INFECTIOUS DISEASES, Infection control < INFECTIOUS DISEASES

SCHOLARONE™
Manuscripts

I, the Submitting Author has the right to grant and does grant on behalf of all authors of the Work (as defined in the below author licence), an exclusive licence and/or a non-exclusive licence for contributions from authors who are: i) UK Crown employees; ii) where BMJ has agreed a CC-BY licence shall apply, and/or iii) in accordance with the terms applicable for US Federal Government officers or employees acting as part of their official duties; on a worldwide, perpetual, irrevocable, royalty-free basis to BMJ Publishing Group Ltd ("BMJ") its licensees and where the relevant Journal is co-owned by BMJ to the co-owners of the Journal, to publish the Work in this journal and any other BMJ products and to exploit all rights, as set out in our licence.

The Submitting Author accepts and understands that any supply made under these terms is made by BMJ to the Submitting Author unless you are acting as an employee on behalf of your employer or a postgraduate student of an affiliated institution which is paying any applicable article publishing charge ("APC") for Open Access articles. Where the Submitting Author wishes to make the Work available on an Open Access basis (and intends to pay the relevant APC), the terms of reuse of such Open Access shall be governed by a Creative Commons licence – details of these licences and which Creative Commons licence will apply to this Work are set out in our licence referred to above.

Other than as permitted in any relevant BMJ Author's Self Archiving Policies, I confirm this Work has not been accepted for publication elsewhere, is not being considered for publication elsewhere and does not duplicate material already published. I confirm all authors consent to publication of this Work and authorise the granting of this licence.

For peer review only

Estimating the COVID-19 epidemic trajectory and hospital capacity requirements in South West England: a mathematical modelling framework

Ross D. Booton[1], Louis MacGregor[2,3], Lucy Vass[1,2], Katharine J. Looker[2,3], Catherine Hyams[4], Philip D. Bright[5], Irasha Harding[6], Rajeka Lazarus[7], Fergus Hamilton[8], Daniel Lawson[9], Leon Danon[10], Adrian Pratt[11], Richard Wood[12,13], Ellen Brooks-Pollock[1,2,3,†], Katherine M.E. Turner[1,2,3,13,†,*]

[1]Bristol Veterinary School, University of Bristol, Bristol, UK

[2]Population Health Science Institute, Bristol Medical School, University of Bristol, Bristol, UK

[3]NIHR Health Protection Research Unit in Behavioural Science and Evaluation

[4]Academic Respiratory Unit, Southmead Hospital, University of Bristol, Bristol, UK

[5]Immunology, Pathology Sciences, North Bristol NHS Trust, Bristol, UK

[6]Consultant in Microbiology, University Hospitals Bristol, Bristol, UK

[7]Consultant in Microbiology and Infectious Diseases, University Hospitals Bristol, Bristol, UK

[8]Infection Science, Southmead Hospital, North Bristol NHS Trust, Bristol, UK

[9]School of Mathematics, University of Bristol, Bristol, UK

[10]College of Engineering, Mathematics and Physical Sciences, University of Exeter, Exeter, UK

[11]Principal BI Analyst Modelling and Analytics, NHS Bristol, North Somerset and South Gloucestershire CCG

[12]Head of Modelling and Analytics, NHS Bristol, North Somerset and South Gloucestershire CCG

[13]Health Data Research UK South-West of England Partnership

[†]should be considered joint senior author

**Correspondence: katy.turner@bristol.ac.uk*

Word count: 4002 (not including references, figures or tables)

Keywords: *Mathematical model, COVID-19, hospital capacity, R value, epidemic trajectory*

Abstract

For peer review only

Objectives: To develop a regional model of COVID-19 dynamics, for use in estimating the number of infections, deaths and required acute and intensive care (IC) beds using the South West of England (SW) as an example case.

Design: Open-source age-structured variant of a susceptible-exposed-infectious-recovered (SEIR) compartmental mathematical model. Latin hypercube sampling and maximum likelihood estimation were used to calibrate to cumulative cases and cumulative deaths.

Setting: SW at a time considered early in the pandemic, where National Health Service (NHS) authorities required evidence to guide localised planning and support decision-making.

Participants: Publicly-available data on COVID-19 patients.

Primary and secondary outcome measures: The expected numbers of infected cases, deaths due to COVID-19 infection, patient occupancy of acute and IC beds and the reproduction ("R") number over time.

Results: SW model projections indicate that, as of the 11th May 2020 (when 'lockdown' measures were eased), 5,793 (95% credible interval, CrI, 2,003-12,051) individuals were still infectious (0.10% of the total SW England population, 95%CrI 0.04-0.22%), and a total of 189,048 (95%CrI 141,580-277,955) had been infected with the virus (either asymptomatically or symptomatically), but recovered, which is 3.4% (95%CrI 2.5-5.0%) of the SW population. The total number of patients in acute and IC beds in the SW on the 11th May 2020 was predicted to be 701 (95%CrI 169-1,543) and 110 (95%CrI 8-464) respectively. The R value in SW England was predicted to be 2.6 (95%CrI 2.0-3.2) prior to any interventions, with social distancing reducing this to 2.3 (95%CrI 1.8-2.9) and lockdown/school closures further reducing the R value to 0.6 (95CrI% 0.5-0.7).

Conclusions: The developed model has proved a valuable asset for regional healthcare services. The model will be used further in the SW as the pandemic evolves, and – as open source software – is portable to healthcare systems in other geographies.

For peer review only

Article summary

Strengths and limitations of this study:

i. Open-source modelling tool available for wider use and re-use.

ii. Customisable to a number of granularities such as at the local, regional and national level.

iii. Supports a more holistic understanding of intervention efficacy through estimating unobservable quantities, e.g. asymptomatic population.

iv. While not presented here, future use of the model could evaluate the effect of various interventions on transmission of COVID-19.

v. Further developments could consider the impact of bedded capacity in terms of resulting excess deaths.

For peer review only

Introduction

Since the initial outbreak in 2019 in Hubei Province, China, COVID-19, the disease caused by Severe Acute Respiratory Syndrome Coronavirus-2 (SARS-CoV-2), has gone on to cause a pandemic [1]. As of 11th May 2020, the Centre for Systems Science and Engineering at Johns Hopkins University reports over 4,000,000 confirmed cases and 250,000 deaths globally [2]. National responses to the outbreak have varied; from severe restrictions on human mobility alongside widespread testing and contact tracing in China [3] to the comparatively relaxed response in Sweden, where lockdown measures have not been enacted [4]. In the UK, advice to socially distance if displaying symptoms was given on the 15th March, while school closures and 'lockdown' measures were implemented from 23rd March onwards [5].

Mathematical modelling has been used to predict the course of the COVID-19 pandemic and to evaluate the effectiveness of proposed and enacted interventions [6–11]. These models have been predominantly aimed at the national level and have largely been based on epidemiological and biological data sourced from the initial epidemic in Wuhan, China [12] and the first large outbreak in Lombardy, Italy [13].

In the UK, the epidemic escalated most rapidly in London [14] and the majority of national modelling is seemingly driven by the trends in London, due to its large case numbers and large population. One of the key issues facing NHS authorities is planning for more localised capacity needs and estimating the timings of surges in demand at a regional or healthcare system level. This is especially challenging given the rapidly evolving epidemiological and biological data; the changes in COVID-19 testing availability (e.g. previously limited and changing eligibility requirements); the uncertainty in the effectiveness of interventions in different contexts; significant and uncertain time-lags between initial infection and hospitalisation or death; and different regions being at different points in the epidemic curve [9]. South West England (SW) is the region with the lowest number of total

For peer review only

cases in England (as of 11th May 2020), lagging behind the national data driven by the earlier epidemic in London [9,14].

COVID-19 results in a significant requirement for hospitalisation, and high mortality amongst patients requiring admission to critical care (particularly amongst those requiring ventilation) [15,16]. In the SW, the population is on average older than in London [17] and is older than the UK as a whole (Table S1). Older age puts individuals at elevated risk of requiring hospital care [18–20]. Consequently, we might expect higher mortality and greater demand for beds in the SW than estimations output from national models that may lack such granularity or risk sensitivity.

However, the SW's first case occurred around 2 weeks later than the first UK case [14]; perhaps implying that the local SW epidemic may be more effectively controlled due to a lower number of baseline cases (than the national average) at the time national interventions were implemented, as well as reduced transmission due to rurality. This sub-national analysis can support in mapping the local epidemic, planning local hospital capacity outside of the main urban centres, and ensuring effective mobilisation of additional support and resources if required. Should demand be lower than expected, reliable forecasts could facilitate more effective use of available resources through re-introducing elective treatments (that had initially been postponed) and responding to other, non-COVID-19 sources of emergency demand.

In this study, taking into account the timeline of UK-wide non-pharmaceutical interventions (social distancing, school closures/lockdown) we illustrate use of our model in projecting estimates for the expected distributions of cases, deaths, asymptomatic and symptomatic infections, and demand for acute and intensive care (IC) beds. We present the model trajectories for SW England using publicly-available data.

Methods

For peer review only

We developed a deterministic, ordinary differential equation model of the transmission dynamics of COVID-19, including age-structured contact patterns, age specific disease progression and demand for hospitalisation, both to acute and IC. We then parameterised the model using available literature and calibrated the model to data from the SW. The model is readily adapted to fit data at sub-regional (e.g. Clinical Commissioning Group), regional or national level. Key assumptions of the model are summarised in the supplementary information.

The model was developed in R and all code and links to source data are freely available (*github.com/rdbooton/bricovmod*). The model is coded using package *deSolve*, with contact matrices from package *socialmixr*, and sampling from package *lhs*.

Model structure

The stages of COVID-19 included within this model are $\boldsymbol{S}$-susceptible, $\boldsymbol{E}$-exposed (not currently infectious, but have been exposed to the virus), $\boldsymbol{A}$- asymptomatic infection (will never develop symptoms), $\boldsymbol{I}$-symptomatic infection (consisting of pre-symptomatic or mild to moderate symptoms), $\boldsymbol{H}$-severe symptoms requiring hospitalisation but not IC, $\boldsymbol{C}$-very severe symptoms requiring IC, $\boldsymbol{R}$-recovered and $\boldsymbol{D}$-death. The total population is $\boldsymbol{N} = \boldsymbol{S} + \boldsymbol{E} + \boldsymbol{A} + \boldsymbol{I} + \boldsymbol{H} + \boldsymbol{C} + \boldsymbol{R} + \boldsymbol{D}$ (Figure 1).

Each compartment $\boldsymbol{X_g}$ is stratified by age-group (0-4, 5-17, 18-29, 30-39, 40-49, 50-59, 60-69, ≥70) where $\boldsymbol{X}$ denotes the stage of COVID-19 (S,E,I,A,H,C,R,D) and $\boldsymbol{g}$ denotes the age group class of individuals. Age groups were chosen to capture key social contact patterns (primary, secondary and tertiary education and employment) and variability in hospitalisation rates and outcomes from COVID-19 especially in older age groups. The total in each age group is informed by recent Office for National Statistics (ONS) estimates [21].

For peer review only

Susceptible individuals become exposed to the virus at a rate governed by the force of infection $\boldsymbol{\lambda_g}$, and individuals are non-infectious in the exposed category. A proportion $\boldsymbol{\delta}$ move from exposed to symptomatic infection and the remaining to asymptomatic infection, both at the latent rate $\boldsymbol{\eta}$. Individuals leave both the asymptomatic and symptomatic compartments at rate $\boldsymbol{\mu}$. All asymptomatic individuals eventually recover and there are no further stages of disease: the rate of leaving the asymptomatic compartment is therefore equivalent to the infectious period, $\boldsymbol{\mu}$. A proportion of symptomatic individuals $\boldsymbol{\gamma_g}$ go on to develop severe symptoms which require hospitalisation, but not intensive care. Once requiring hospitalisation, we assume individuals are no longer infectious to the general population due to self-isolation guidelines restricting further mixing with anyone aside from household members (if unable to be admitted to hospital) or frontline NHS staff (if admitted to hospital). Individuals move out of the acute hospitalised compartment at rate $\boldsymbol{\rho}$, either through death, being moved to intensive care at rate $\boldsymbol{\epsilon}$, or through recovery (all remaining individuals). A proportion $\boldsymbol{\omega_g}$ of patients requiring IC will die at rate $\boldsymbol{\psi}$, while the rest will recover.

The model (schematic in Figure 1) is therefore described by the following differential equations:

$$\textit{Susceptible } \boldsymbol{S_g} \qquad \frac{dS_g}{dt} = -\lambda_g S_g \tag{1a}$$

$$\textit{Exposed } \boldsymbol{E_g} \qquad \frac{dE_g}{dt} = \lambda_g S_g - \eta E_g \tag{1b}$$

$$\textit{Asymptomatic } \boldsymbol{A_g} \qquad \frac{dA_g}{dt} = \eta(1-\delta)E_g - \mu A_g \tag{1c}$$

$$\textit{Infectious } \boldsymbol{I_g} \qquad \frac{dI_g}{dt} = \eta\delta E_g - \mu I_g \tag{1d}$$

$$\textit{Hospitalised in acute bed } \boldsymbol{H_g} \qquad \frac{dH_g}{dt} = \mu\gamma_g I_g - \rho H_g \tag{1e}$$

For peer review only - http://bmjopen.bmj.com/site/about/guidelines.xhtml

Hospitalised in IC $\boldsymbol{C_g}$

$$\frac{dC_g}{dt} = \rho\epsilon H_g - \psi C_g \#(1f)$$

Recovered $\boldsymbol{R_g}$

$$\frac{dR_g}{dt} = \mu A_g + \mu(1-\gamma_g)I_g + (1-\epsilon)(1-\kappa)\rho H_g + (1-\omega_g)\psi C_g \#(1g)$$

Death $\boldsymbol{D_g}$

$$\frac{dD_g}{dt} = (1-\epsilon)\kappa\rho H_g + \omega_g\psi C_g \#(1h)$$

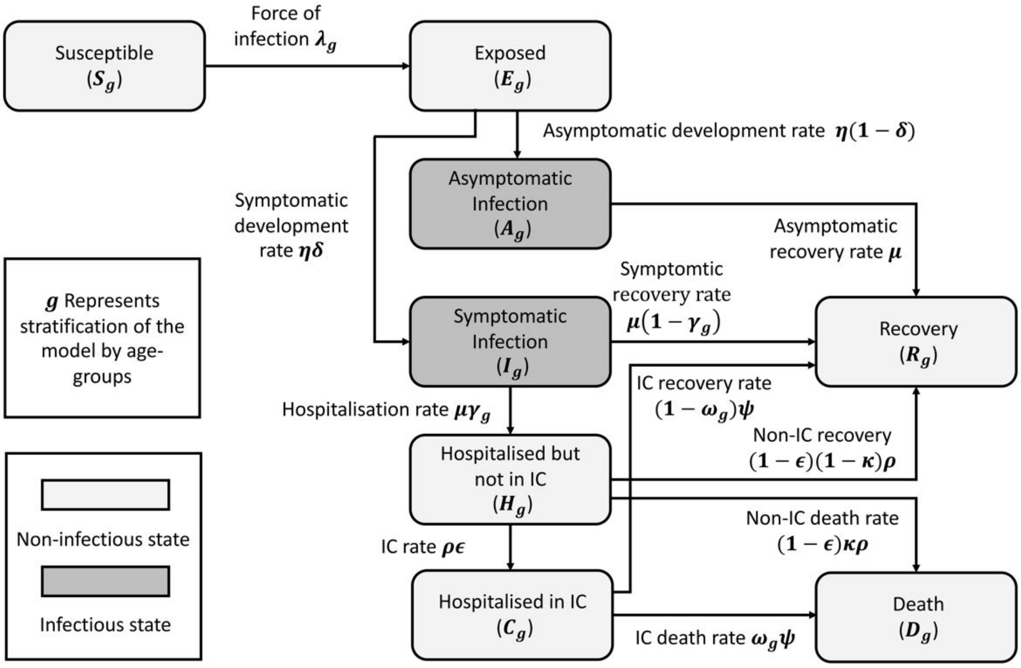

Figure 1: Compartmental flow model diagram depicting stages of disease and transitions between states. Asymptomatic infection represents the number of people never showing symptoms, while symptomatic infection includes all those who show pre-symptomatic / mild symptoms to those who show more severe symptoms (pre-hospitalisation). Those who are hospitalised first occupy a non-IC bed (acute bed) after which they can either move into IC, recover or die. Those in IC can either recover or die at an increased rate compared to those in acute beds. This model does not capture those deaths which occur outside of hospital as a result of COVID-19.

Contact patterns under national interventions

We assume the population is stratified into pre-defined age groups with age-specific mixing pattern represented by a contact matrix $\boldsymbol{M}$ with an element of $\boldsymbol{m_{ij}}$ representing the contacts between someone of age group $\boldsymbol{i} \in \boldsymbol{G}$ with someone in age group $\boldsymbol{j} \in \boldsymbol{G}$. The baseline contact matrix (with no interventions in place) is taken from the POLYMOD survey conducted in the United Kingdom [22]. The contact pattern may also be influenced by a range of interventions (social distancing was encouraged on 15th March 2020, schools were closed and lockdown occurred on 23rd March 2020). We implement these interventions by assuming that the percentage of 0-18-year olds attending school after the 23rd March 2020 was 5% (reducing all contacts between school age individuals by 95%) and that social distancing reduced all contacts by 0-50%. We take the element-wise minimum for each age group's contact with another age group from all active interventions (distancing, schools/lockdown). A study on post-lockdown contact patterns (CoMix [11]) is used to inform contacts after lockdown (first survey 24th March 2020, with an average of 73% reduction in daily contacts observed per person compared to POLYMOD).

Moving between contact matrices of multiple interventions was implemented by assuming a phased, linear decrease. After lockdown we vary a parameter (*endphase*) to capture the time taken to fully adjust (across the population, on average) to the new measures (allowed to vary from 1 to 31 days). This assumption represents the time taken for individuals to fully adapt to new measures (and household transmission), and is in line with data on the delay in the control of COVID-19 (reductions in hospital admissions and deaths after lockdown) [23]. The parameter *endphase* can be interpreted as accounting for the time taken to adjust to all interventions (and not just lockdown).

The force of infection

For peer review only

The age-specific force of infection $\boldsymbol{\lambda_g}$, depends on the proportion of the population who are infectious (asymptomatic $\boldsymbol{A_g}$ and symptomatic $\boldsymbol{I_g}$ only) and probability of transmission $\boldsymbol{\beta}$:

$$\lambda_g = \beta \sum_{i \in G} m_{ig} \left(\frac{A_i}{N_g} + \frac{I_i}{N_g} \right) \#(2)$$

The basic reproduction number R_0

The basic reproduction number R_0 of COVID-19 is estimated to be 2.79 $\pm$ 1.16 [24]. We include this estimate within our model by calculating the maximum eigenvalue of the contact matrix $\boldsymbol{M}$, and allowing the transmission parameter to vary such that R_0 is equal to the maximum eigenvalue of $\boldsymbol{M}$ multiplied by the infectious period μ and the transmission parameter β. This gives the value for the initial basic reproduction number R_0, which changes as the contact patterns change as lockdown and other interventions are implemented.

Parameter estimates and data sources

Model parameters are detailed in Table 1. We used available published literature to inform parameter estimates. We used the following publicly-available metrics for model fitting: regional cumulative cases in SW England (tested and confirmed case in hospital), and deaths (daily/cumulative counts) from the Public Health England COVID-19 dashboard [14], and ONS weekly provisional data on COVID-19 related deaths [25]. The case data is finalised prior to the previous five days, so we include all data until 14th May 2020, based on data reported until 18th May 2020. The mortality data from ONS does not explicitly state the number of COVID-19 related deaths occurring in hospital, but they do report this value nationally (83.9% of COVID-19 deaths in hospital, as of 17th April 2020). We assume that this percentage applies to the SW data and re-scale the mortality to 83.9% to represent an estimate of total deaths in hospital.

For peer review only

Model calibration

Using the available data (Table 1), we define ranges for all parameters in our model and sample all parameters simultaneously between these minimum and maximum values assuming uniform distributions using Latin Hypercube Sampling (statistical method for generating random parameters from multidimensional distribution), for a total of 100,000 simulations. We used maximum likelihood estimation on total cumulative cases and cumulative deaths with a Poisson negative log likelihood calculated and summed over all observed and predicted points. For i observed cases X_i (from data), and i predicted cases Y_i (from simulations of the model), we select the best 100 parameter sets which maximise the log-likelihood $\sum X_i \log(Y_i) - Y_i$ from the total sample of 100,000 simulations. The best 100 samples were taken as part of a bias-variance trade-off (Supplementary Information, Sensitivity analysis), and the qualitative inferences would not change with other choices of sample size. For each data point (taken from cases and deaths) we calculate this log-likelihood, and weight each according to the square-root of the mean of the respective case or death data. This ensures that we are considering case and death data equally within our likelihood calculations.

Symbol	*Description*	*Uniform prior (min and max) or point estimate*
$\mathbf{1/\eta}$	Duration of the non-infectious exposure period	5.1 days [26]
$\boldsymbol{\delta}$	Percentage of infections which become symptomatic	82.1% [27]; Vary between 73.15 – 91.05%
$\mathbf{1/\mu}$	Duration of symptoms whilst not hospitalised (independent of outcome)	Vary between 2 – 14 days
$\mathbf{1/\rho}$	Duration of stay in acute bed (independent of outcome)	Vary between 2 – 14 days
$\boldsymbol{\gamma_g}$	Percentage of symptomatic cases which will require hospitalisation	0-4 = 0.00%, 5-17 = 0.0408%, 18-29 = 1.04%, 30-39 = 2.04-7.00%, 40-49 = 2.53-8.68%, 50-59 = 4.86-16.7%, 60-69 = 7.01-24.0%, 70+ = 9.87-37.6% [16]
$\mathbf{1/\psi}$	Duration of stay in IC bed (independent of outcome)	3 – 11 days [28]
$\boldsymbol{\epsilon}$	Percentage of those requiring hospitalisation who will require IC	Vary between 0 – 30%
$\boldsymbol{\omega_g}$	Percentage of those requiring IC who will die	0-4 = 0.00%, 5-17 = 0.00%, 18-29 = 18.1%, 30-39 = 18.1%, 40-49 = 24.7%,

For peer review only

		50-59 = 39.3%, 60-69 = 53.9%, 70+ = 65.3% [28]
$\boldsymbol{\kappa}$	Percentage of those requiring acute beds (but not IC) who will die	Vary between 5 – 35%
school	Percentage of 0-18-year olds attending school after 23/03/2020	Assume 5%
distancing	Percentage reduction in contact rates due to social distancing after 15/03/2020	Vary between 0 – 50%
lockdown	Percentage reduction in contact rates due to lockdown after 23/03/2020	Retail/recreation: Bristol 86% Bath 90% Plymouth 85% Gloucs 84%, Somerset 82%, Devon 85%, Dorset 84% [29]<br>Transit stations: Bristol 78% Bath 71% Plymouth 65% Gloucs 69%, Somerset 67%, Devon 66%, Dorset 63% [29]<br>Vary between 63 - 90%
$\boldsymbol{R_0}$	Initial reproductive number of COVID-19	1.63 – 3.95 [24]

Table 1: Parameter estimates used in the model and their sources. The distributions of unknown parameters are shown in Figure S1 for the best 100 fits.

Model outputs

For each of the 100 best parameter sets we run the model until 11th May 2020 and output the cumulative cases and deaths in the SW. We output the predicted proportion of the population who are infectious and who have ever been infected over time. Finally, we estimate the daily and cumulative patterns of admission to and discharge from hospital (intensive care and acute) and cumulative mortality from COVID-19.

Results and outputs

From 100,000 simulated parameter sets, we selected the best 100 baseline model fits on the basis of agreement to the calibration data on daily confirmed COVID-19 cases and weekly mortality due to COVID-19 in SW England. The distribution of the best fitting values are shown in Figure S1a (and the priors in Figure S1b). All results are shown with median and 95% credible intervals (95%CrI).

For peer review only

On 11th May 2020, the reported cumulative number of individuals with (lab confirmed) COVID-19 was 7,116 in SW England [14], and the most recent report on total cumulative deaths showed that 2,306 had died from COVID-19 (as of 8th May 2020) [25].

Estimating the total proportion of individuals in South West England with COVID-19

Figure 2 shows the projected numbers of exposed, recovered and infectious (asymptomatic and symptomatic infections) until lockdown measures were lessened on the 11th May 2020. On this date, the model predicts that a total of 5,793 (95%CrI 2,003 – 12,051) were infectious (0.10% of the total SW England population, 95%CrI 0.04 – 0.22%). The model also predicts that a total of 189,048 (95%CrI 141,580 – 277,955) have had the virus but recovered (either asymptomatically or symptomatically), which is 3.4% (95%CrI 2.5 – 5.0%) of the SW population (not infectious and not susceptible to reinfection).

For peer review only

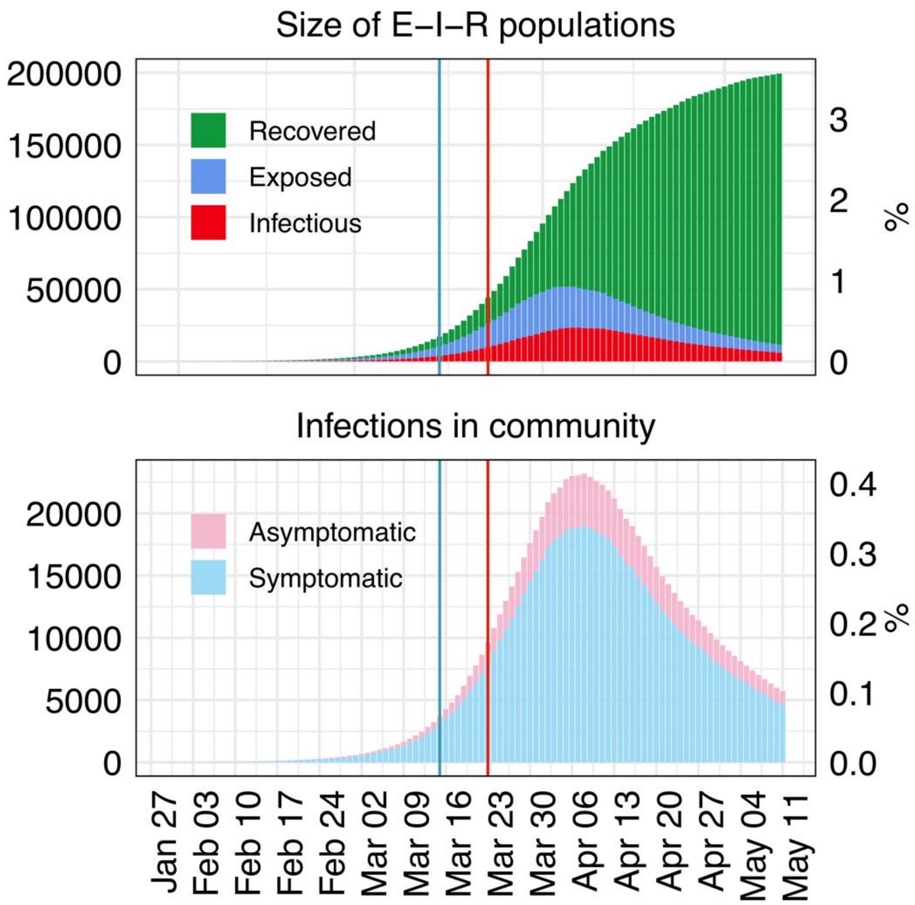

256

257 **Figure 2:** The predicted median size of the exposed (E), infectious (I) and recovered classes (R), along with the
258 size of asymptomatic and symptomatic individuals on each day in SW England until 11th May 2020. Blue and red
259 vertical lines represent the date the government introduced social distancing and school closures/lockdown,
260 respectively.

review only

Estimating the total COVID-19 hospitalised patients in acute and intensive care beds

The total number of patients in acute (non-intensive care) hospital beds across SW England was projected to be 701 (95%CrI 169 – 1,543) and the total number of patients in intensive care hospital beds was projected to be 110 (95%CrI 8 – 464) on the 11th May 2020 (Figure 3). Note that these ranges are quite large due to the uncertainty in the data and as more data becomes available these predictions will change.

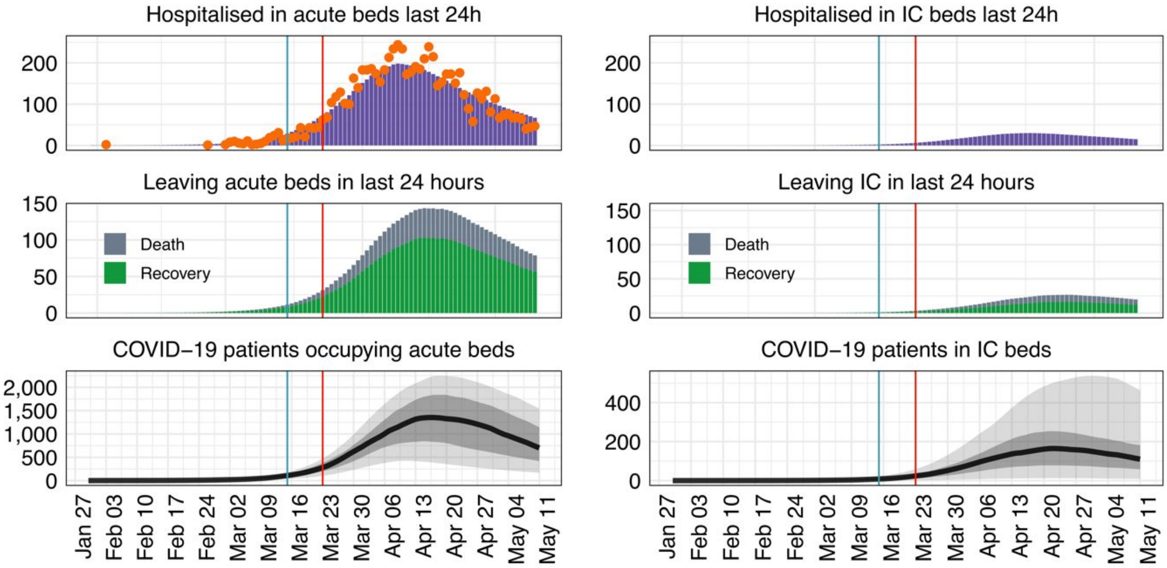

Figure 3: The predicted number of hospitalised patients in acute and intensive care beds in the SW until 11th May 2020. The number of daily incoming patients diagnosed with COVID-19 are shown in orange (from SW daily case data [14]), 95% credible intervals are shown in light grey, 50% in dark grey and the median value of the fits is highlighted in black. The shaded region indicates the prediction of the model from the data. Blue and red vertical lines represent the date the government introduced social distancing, and school closures/lockdown, respectively.

Estimating the reproduction number under interventions

Figure 4 shows the model prediction for the reproduction ("R") number over time until 11th May 2020, when lockdown measures were relaxed. All interventions (social distancing, school closures/lockdown) had a significant impact on the reproductive number for COVID-19 in the

South West of England. We predict that prior to any interventions R was 2.6 (95%CrI 2.0 – 3.2), and the introduction of social distancing reduced this number to 2.3 (95%CrI 1.8 – 2.9). At the minimum, R was 0.6 (95%CrI 0.5 – 0.7) after all prior interventions were enacted and adhered to (social distancing, school closures and lockdown).

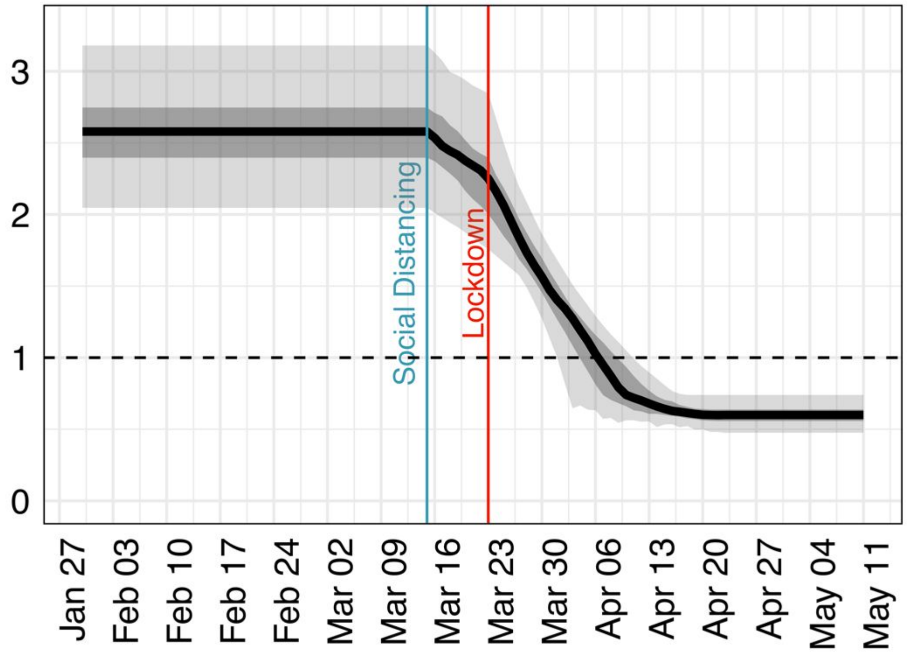

Figure 4: The effect of interventions on estimates of R (y-axis) over time until the 11th May 2020.

Discussion

We have developed a deterministic ordinary differential equation model of the epidemic trajectory of COVID-19 focussing on acute and IC hospital bed capacity planning to support local NHS authorities, calibrating to SW-specific data. The model is age-structured and includes time-specific implementation of current interventions (following advice and enforcement of social distancing, school closures and lockdown) to predict the potential range of COVID-19 epidemic trajectories.

Using the publicly-available data on cases and deaths, combined with the early estimates of parameters from early epidemics in other settings, we predict that on the 11th May 2020 a total of 5,793 (95%CrI 2,003 – 12,051) were infectious, which equates to 0.10% (95%CrI 0.04 – 0.22%) of the total SW population. In addition, we find that the model predicts a total of 189,048 (95%CrI 141,580 – 277,955) have had the virus but recovered, which is 3.4% (95%CrI 2.5 – 5.0%) of the SW population.

We also estimate that the total number of patients in acute hospital beds in SW England on 11th May 2020 was 701 (95%CrI 169 – 1,543) and in IC were 110 (95%CrI 8 – 464), while the R number has decreased from 2.6 (95%CrI 2.0 – 3.2) to 0.6 (95%CrI 0.5 – 0.7) after all interventions were enacted and fully adhered to.

The fits generally agree well with both the daily case data, and the cumulative count of deaths in the SW, although the model overestimates the case data at early stages, and underestimates later on (which can see seen in Figure S2a, and a scatter plot of expected versus observed outputs in Figure S2b). This could be because we are using formal fitting methods, or from the under-reporting of cases in the early epidemic.

The primary strength of this study is that we have developed generalisable and efficient modelling code incorporating disease transmission, interventions and hospital bed demand

For peer review only

which can be adapted for use in other regional or national scenarios, with the model available on GitHub for open review and use (*github.com/rdbooton/bricovmod*). We have worked closely with the NHS and at Clinical Commissioning Group (CCG) level to ensure the model captures key clinical features of disease management in SW hospitals and provides output data in a format relevant to support local planning. We combined local clinical expertise with detailed literature searches, to ensure reasonable parameter ranges and assumptions in the presence of high levels of parameter uncertainty.

The main challenge of this work is in balancing the urgent need locally for prediction tools which are up to date (i.e. not relying on the national trends to inform capacity planning), versus more exhaustive and robust methods for model comparison. The latter of which uses existing models and more time-consuming (but more robust) data fitting methods [30,31]. However, we believe that release of this paper and sharing of model code will facilitate multidisciplinary collaboration, rapid review and support future model comparison and uncertainty analyses [31].

As with all models of new infections there are significant parameter uncertainties. Rapidly emerging literature is exploring a wide range of biological and epidemiological factors concerning COVID-19, but due to the worldwide nature of these studies, often parameter bands are wide and may be context specific. For example, early estimates of the basic reproduction number ranged from 1.6 to 3.8 in different locations [32,33], with an early estimate of 2.4 used in UK model projections [8]. In addition, the information which informs our parameter selection is rapidly evolving as new data is made available, sometimes on a daily basis. From our initial analysis, we identified the following parameters as critical in determining the epidemic trajectory within our model – the percentage of infections which become symptomatic, the recovery time for cases which do not require hospital, the period between acute and IC occupancy, the length of stay in IC, the probability of transmission per contact

For peer review only

and the gradual implementation of lockdown rather than immediate effect. Other parameters (such as the percentage reduction in school-age contacts from school closures) did not seem to influence the dynamic trajectory as strongly – and thus we assume point estimates for these parameters. However, for example assuming that 95% of school-age contacts are reduced as a direct result of school closures is perhaps an overestimate, and future modelling work should address these uncertainties and their impacts on the epidemic trajectory of COVID-19 (but in this case, this value was somewhat arbitrary, and the assumption was used in the absence of school-age contact survey data). More research is urgently needed to refine these parameter ranges and to validate these biological parameters experimentally.

We have also assumed that there is no nosocomial transmission of infection between hospitalised cases and healthcare workers, as we do not have good data for within-hospital transmission. However frontline healthcare staff were likely to have been infected early on in the epidemic [34], which could have implications for our predicted epidemic trajectory. Our model also assumes a closed system, which may not strictly be true due to continuing essential travel. But given that up until 11th May, travel restrictions are very severe due to lockdown measures [5], any remaining inter-regional travel is likely to have minimal effects on our model outputs.

Similar to most other COVID-19 models, we use a variant on a susceptible-exposed-infectious-recovered (SEIR) structure [8–10,16,30,35,36]. We do not spatially structure the population as in other UK modelling [9,10], but we do include age-specific mixing based on POLYMOD data [22], and the post-lockdown CoMix study [11]. We also explicitly measure the total asymptomatic infection, and the total in each of the clinically relevant hospital classes (acute or IC), which is a strength of our approach. Future models could also take into account local bed capacity within hospitals (including Nightingale centres) and accommodate the effect of demand outstripping supply leading to excess deaths, inclusive of non-hospital-based death

For peer review only

such as is occurring within care homes. As with all modelling, we have not taken into account all possible sources of modelling misspecification. Some of these misspecifications will tend to increase the predicted epidemic period, and others will decrease it. One factor that could significantly change our predicted epidemic period is the underlying structure within the population leading to heterogeneity in the average number of contacts under lockdown e.g. key workers have high levels of contact but others are able to minimise contacts effectively, this might lead to an underestimate of ongoing transmission, but potentially an overestimate of the effect of releasing lockdown. We also know that there are important socio-economic considerations in determining people's ability to stay at home and particularly to work from home [37].

Early UK modelling predicted the infection peak to be reached roughly 3 weeks from the initiation of severe lockdown measures, as taken by the UK government in mid-March [8]. A more recent study factoring spatial distribution of the population indicated the peak to follow in early April due to R_0 reducing to below 1 in many settings in weeks following lockdown [9]. Other modelling indicated that deaths in the UK would peak in mid-late April; furthermore, that the UK would not have enough acute and IC beds to meet demand [38]. While modelling from the European Centre for Disease Prevention and Control estimated peak cases to occur in most Euopean countries in mid-April [20], these estimations were largely at a national level. Due to the expected lag of other regions behind London, these estimated peaks are likely to be shifted further into the future for the separate regions of the UK, and as shown by our model occurred in early- to mid-April. This is also likely to be true for future peaks which may result from relaxing lockdown.

Outside of the UK, similar modelling from France [35] (which went into lockdown at a similar time the UK on 17th March), predicted the peak in daily IC admissions at the end of March. Interestingly however, when dissected by region, the peak in IC bed demand varied by

For peer review only

roughly 2 weeks. Swiss modelling similarly predicted a peak in hospitalisation and numbers of patients needing IC beds in early April, after lockdown implementation commenced on 17th March [36]. US modelling [39] disaggregated by State, also highlights the peak of excess bed demand varies geographically, with this peak ranging between the 2nd week of April, through to May dependent on the State under consideration. The modelling based in France also cautioned that due to only 5.7% of the population having been infected by 11th May when the restrictions would be eased, the population would be vulnerable to a second epidemic peak thereafter [35].

The ONS in England estimated that an average of 0.25% of the population had COVID-19 between the 4th and 17th May 2020 (95% confidence interval: 0.16 – 0.38%) [40], which is greater than the 0.10% (95%CrI 0.04 – 0.22%) we found with our model (on 11th May 2020), but with some overlap. In addition, the ONS estimated that 6.78% (95%CrI 5.21 – 8.64%) tested positive for antibodies to COVID-19 up to 24th May 2020 in England [41], and Public Health England estimated that approximately 4% (2 – 6%) tested positive for antibodies to COVID-19 between the 20th and 26th April 2020 in the SW [42]. Comparing to our model, 3.4% (95%CrI 2.5 – 5.0%) had recovered on the 11th May 2020 (2 weeks later), demonstrating that our model estimates may be within sensible bounds, and further highlighting the need for more regional estimates of crucial epidemiological parameters and seroprevalence. We have assumed that individuals are not susceptible to reinfection within the model timeframe, however in future work it will be important to explore this assumption. It is not known what the long term pattern of immunity to COVID-19 will be [43], and this will be key to understanding the future epidemiology in the absence of a vaccine or effective treatment options.

With this in mind, our findings demonstrate that there are still significant data gaps – and in the absence of such data, mathematical models can provide a valuable asset for local

For peer review only

and regional healthcare services. This regional model will be used further in the SW as the pandemic evolves and could be used within other healthcare systems in other geographies to support localised predictions. Controlling intervention measures at a more local level could be made possible through monitoring and assessment at the regional level through a combination of clinical expertise and local policy, guided by localised predictive forecasting as presented in this study.

Funding statement

This work was funded with support from the Elizabeth Blackwell Institute, Bristol UNCOVER (Bristol COVID Emergency Research) and Medical Research Council UK. LM, KJL, EBP and KMET acknowledge support from the NIHR Health Protection Research Unit in Behavioural Science and Evaluation at the University of Bristol.

This work was also supported by Health Data Research UK, which is funded by the UK Medical Research Council, Engineering and Physical Sciences Research Council, Economic and Social Research Council, National Institute for Health Research, Chief Scientist Office of the Scottish Government Health and Social Care Directorates, Health and Social Care Research and Development Division (Welsh Government), Public Health Agency (South Western Ireland), British Heart Foundation and Wellcome.

Competing interests statement

We declare no competing interests.

Author contributions

For peer review only

Conception and design of the study: LD, EBP. Acquisition of data: RDB, EBP, RW, KMET. Mathematical modelling: RDB, LM, LD, EBP, RW, KMET. Coding and simulations: RDB. Analysis and interpretation of results: RDB, LM, LV, KJL, CH, PB, IH, RL, FH, DL, LD, AP, EBP, RW, KMET. Writing and drafting of the manuscript: RDB, LM, LV, KJL, CH, PB, IH, RL, FH, DL, LD, AP, EBP, RW, KMET. Approval of the submitted manuscript: RDB, LM, LV, KJL, CH, PB, IH, RL, FH, DL, LD, AP, EBP, RW, KMET.

Data sharing statement

All model code is open source and available for download on GitHub https://github.com/rdbooton/bricovmod. All data is freely available via the GOV.UK COVID-19 dashboard [14] and ONS [21,25].

Ethics and dissemination

No ethical approval was required for this mathematical modelling work.

Patient and Public Involvement

This research was done without patient involvement. Patients were not invited to comment on the study design and were not consulted to develop patient relevant outcomes or interpret the results. Patients were not invited to contribute to the writing or editing of this document for readability or accuracy.

For peer review only

References

1. World Health Organization. WHO Director-General's opening remarks at the mission briefing on COVID-19. 2020 [cited 27 Apr 2020]. Available: https://www.who.int/dg/speeches/detail/who-director-general-s-opening-remarks-at-the-media-briefing-on-covid-19---11-march-2020
2. Johns Hopkins University. COVID-19 Data Center. COVID-19 Case Tracker. [cited 27 Apr 2020]. Available: https://coronavirus.jhu.edu/
3. Payne C. State intervention in China. Nat Hum Behav. 2020;1.
4. Public Health Agency of Sweden. Public Health Authority regulations and general advice on everyone's responsibility to prevent the infection of COVID-19. 2020 [cited 7 Apr 2020]. Available: https://www.folkhalsomyndigheten.se/publicerat-material/publikationsarkiv/h/hslf-fs-202012/
5. Public Health England. Guidance on social distancing for everyone in the UK. In: Guidance on social distancing for everyone in the UK [Internet]. 2020 [cited 27 Apr 2020]. Available: https://www.gov.uk/government/publications/covid-19-guidance-on-social-distancing-and-for-vulnerable-people/guidance-on-social-distancing-for-everyone-in-the-uk-and-protecting-older-people-and-vulnerable-adults
6. Prem K, Liu Y, Russell TW, Kucharski AJ, Eggo RM, Davies N, et al. The effect of control strategies to reduce social mixing on outcomes of the COVID-19 epidemic in Wuhan, China: a modelling study. Lancet Public Heal. 2020.
7. Flaxman S, Mishra S, Gandy A, Unwin JT, Coupland H, Mellan TA, et al. Estimating the number of infections and the impact of non-pharmaceutical interventions on COVID-19 in 11 European countries. Imp Coll London COVID-19 Report No 13. 2020.
8. Ferguson NM, Laydon D, Gemma Nedjati-Gilani, Natsuko Imai, Kylie Ainslie MB,

For peer review only

Sangeeta Bhatia, Adhiratha Boonyasiri, Zulma Cucunubá, Gina Cuomo-Dannenburg, Amy Dighe I, Dorigatti, Han Fu, Katy Gaythorpe, Will Green, Arran Hamlet, Wes Hinsley, Lucy C Okell S van, Elsland, Hayley Thompson, Robert Verity, Erik Volz, Haowei Wang, Yuanrong Wang PGW, et al. Impact of non-pharmaceutical interventions (NPIs) to reduce COVID19 mortality and healthcare demand. Imp Coll London COVID-19 Report No 9. 2020.

9. Challen R, Tsaneva-Atanasova K, Pitt M. Estimates of regional infectivity of COVID-19 in the United Kingdom following imposition of social distancing measures. medRxiv. 2020.

10. Danon L, Brooks-Pollock E, Bailey M, Keeling MJ. A spatial model of CoVID-19 transmission in England and Wales: early spread and peak timing. medRxiv. 2020.

11. Jarvis CI, Zandvoort K Van, Gimma A, Prem K, Group CC-19 working, Klepac P, et al. Quantifying the impact of physical distance measures on the transmission of COVID-19 in the UK. medRxiv. 2020.

12. The Novel Coronavirus Pneumonia Emergency Response Epidemiology Team. The epidemiological characteristics of an outbreak of 2019 novel coronavirus diseases (COVID-19) — China, 2020. Chin J Epidemiol. 2020;41.

13. Onder G, Rezza G, Brusaferro S. Case-fatality rate and characteristics of patients dying in relation to COVID-19 in Italy. JAMA - Journal of the American Medical Association. 2020.

14. Public Health England. Coronavirus (COVID-19) in the UK: data dashboard. 2020 [cited 28 Apr 2020]. Available: https://coronavirus.data.gov.uk/#

15. Yang X, Yu Y, Xu J, Shu H, Xia J, Liu H, et al. Clinical course and outcomes of critically ill patients with SARS-CoV-2 pneumonia in Wuhan, China: a single-centered, retrospective, observational study. Lancet Respir Med. 2020.

For peer review only

16. Verity R, Okell LC, Dorigatti I, Winskill P, Whittaker C, Imai N, et al. Estimates of the severity of coronavirus disease 2019 : a model-based analysis. Lancet Infect Dis. 2020.

17. McCurdy C. Ageing, fast and slow: when place and demography collide. Resolut Found. 2019. Available: https://wwwresolutionfoundationorg/app/uploads/2019/10/Ageing-fast-and-slowpdf

18. Yang J, Zheng Y, Gou X, Pu K, Chen Z, Guo Q, et al. Prevalence of comorbidities in the novel Wuhan coronavirus (COVID-19) infection: a systematic review and meta-analysis. Int J Infect Dis. 2020.

19. Zhou F, Yu T, Du R, Fan G, Liu Y, Liu Z, et al. Clinical course and risk factors for mortality of adult inpatients with COVID-19 in Wuhan, China: a retrospective cohort study. Lancet. 2020.

20. Stockholm: ECDC (European Centre for Disease Prevention and Control). Coronavirus disease 2019 (COVID-19) pandemic: increased transmission in the EU/EEA and the UK – seventh update, 25 March 2020. 2020. Available: https://www.ecdc.europa.eu/sites/default/files/documents/RRA-seventh-update-Outbreak-of-coronavirus-disease-COVID-19.pdf

21. Office for National Statistics. Estimates of the population for the UK, England and Wales, Scotland and Northern Ireland. 2020. Available: https://www.ons.gov.uk/peoplepopulationandcommunity/populationandmigration/populationestimates/datasets/populationestimatesforukenglandandwalesscotlandandnorthernireland

22. Mossong J, Hens N, Jit M, Beutels P, Auranen K, Mikolajczyk R, et al. Social contacts and mixing patterns relevant to the spread of infectious diseases. PLoS Med. 2008. doi:10.1371/journal.pmed.0050074

For peer review only

23. Stedman M, Davies M, Anderson SG, Lunt M, Verma A, Heald A. A phased approach to unlocking during the COVID-19 pandemic; Lessons from trend analysis. medRxiv. 2020. doi:10.1101/2020.04.20.20072264

24. Liu Y, Gayle AA, Wilder-Smith A, Rocklöv J. The reproductive number of COVID-19 is higher compared to SARS coronavirus. J Travel Med. 2020;27. doi:10.1093/jtm/taaa021

25. Office for National Statistics. Deaths registered in England and Wales, provisional 2020: up to week ending 17 April 2020. 2020. Available: https://www.ons.gov.uk/peoplepopulationandcommunity/birthsdeathsandmarriages/deaths/datasets/weeklyprovisionalfiguresondeathsregisteredinenglandandwales

26. Lauer SA, Grantz KH, Bi Q, Jones FK, Zheng Q, Meredith HR, et al. The incubation period of coronavirus disease 2019 (COVID-19) from publicly reported confirmed cases: estimation and application. Ann Intern Med. 2020.

27. Mizumoto K, Kagaya K, Zarebski A, Chowell G. Estimating the asymptomatic proportion of coronavirus disease 2019 (COVID-19) cases on board the Diamond Princess cruise ship, Yokohama, Japan, 2020. Eurosurveillance. 2020.

28. ICNARC COVID-19 study case mix programme database. ICNARC report on COVID-19 in critical care 15 May 2020. 2020. Available: https://www.icnarc.org/Our-Audit/Audits/Cmp/Reports

29. Google. United Kingdom COVID-19 Community Mobility Report: Mobility changes April 5 2020. 2020. Available: https://www.gstatic.com/covid19/mobility/2020-04-05_GB_Mobility_Report_en.pdf

30. Wynants L, Van Calster B, Bonten MMJ, Collins GS, Debray TPA, De Vos M, et al. Prediction models for diagnosis and prognosis of covid-19 infection: systematic review and critical appraisal. BMJ. 2020.

For peer review only

31. Latif S, Usman M, Manzoor S, Iqbal W, Qadir J, Tyson G, Castro I, Razi A, Boulos MN CJ. Leveraging Data Science To Combat COVID-19: A Comprehensive Review. 2020.

32. Kucharski AJ, Russell TW, Diamond C, Liu Y, Edmunds J, Funk S, et al. Early dynamics of transmission and control of COVID-19: a mathematical modelling study. Lancet Infect Dis. 2020.

33. Read JM, Bridgen JRRE, Cummings DATA, Ho A, Jewell CP. Novel coronavirus 2019-nCoV: early estimation of epidemiological parameters and epidemic predictions. medRxiv. 2020.

34. The Centre for Evidence-Based Medicine Website. How many healthcare workers are infected? 2020 [cited 23 Apr 2020]. Available: https://wwwcebmnet/covid-19/covid-19-how-many-healthcare-workers-are-infected/

35. Salje H, Kiem C, Lefrancq N, Courtejoie N, Bosetti P. Estimating the burden of SARS-CoV-2 in France. HAL ID pasteur-02548181. 2020.

36. Althaus C. Real-time modeling and projections of the COVID-19 epidemic in Switzerland. Inst Soc Prev Med Univ Bern, Switz. 2020. Available: https://ispmberngithubio/covid-19/swiss-epidemic-model/

37. Dingel J, Neiman B. How Many Jobs Can be Done at Home? NBER Work Pap. 2020.

38. Institute for Health Metrics and Evaluation. New COVID-19 forecasts for Europe: Italy & Spain have passed the peak of their epidemics; UK, early in its epidemic, faces a fast-mounting death toll. 2020. Available: http://www.healthdata.org/news-release/new-covid-19-forecasts-europe-italy-spain-have-passed-peak-their-epidemics-uk-early-its

39. IHME COVID-19 health service utilization forecasting team. Forecasting COVID-19 impact on hospital bed-days, ICU-days, ventilator-days and deaths by US state in the

For peer review only

582 next 4 months. medxriv. 2020.

583 40. ONS. Coronavirus (COVID-19) Infection Survey pilot: England, 21 May 2020. 2020.

584 41. ONS. Coronavirus (COVID-19) Infection Survey pilot: 28 May 2020. 2020.

585 42. Public Health England. Weekly Coronavirus Disease 2019 (COVID-19) Surveillance
586 Report, Week 22. 2020. Available:
587 https://assets.publishing.service.gov.uk/government/uploads/system/uploads/attachmen
588 t_data/file/888254/COVID19_Epidemiological_Summary_w22_Final.pdf

589 43. Altmann DM, Douek DC, Boyton RJ. What policy makers need to know about
590 COVID-19 protective immunity. The Lancet Comments. 2020.

591

592

593

For peer review only

Supplementary information

Age group	Population size SW England	Percent population size SW England	Percent population size UK
0-4	296,357	5.3%	6.0%
5-18	805,965	14.4%	15.4%
18-29	806,885	14.4%	15.4%
30-39	654,469	11.7%	13.4%
40-49	684,872	12.2%	12.8%
50-59	782,317	14.0%	13.4%
60-69	671,294	12.0%	10.5%
70+	897,576	16.0%	13.1%

Table S1: Demography of SW England compared to the UK.

For peer review only

Key assumptions used in the model

i. *Closed, static population size with no immigration or emigration due to the model being run over a short period of time, and current travel restrictions which should prevent significant movement of individuals in and out of the South West.*
ii. *No nosocomial transmission.*
iii. *Recovered individuals are not susceptible to reinfection within the timeframe of the model horizon.*
iv. *Trajectory of model outputs is assuming that there is no easing of lockdown situation over the timeframe of the model horizon.*
v. *There is no restriction on number of hospital (IC and acute) beds available.*
vi. *Asymptomatic and symptomatic are equally infectious and recover at the same rate.*
vii. *95% reduction in 0-18-year old contacts from school closures*
viii. *Range of 0 - 50% reduction in contacts from social distancing and range of 63 - 90% reduction in contact rates due to lockdown*
ix. *The reduction of any given contact rate is taken to be the minimum from lockdown, school closures and social distancing.*
x. *R_0 is sampled between 2.79 +/- 1.16 and the infectious period and transmission probability are chosen to achieve this*
xi. *Proportion symptomatic which require hospitalisation depends on age, with increased risk for older age groups*
xii. *Proportion in IC who will die depends on age, with increased risk for older age groups*
xiii. *The percentage of infections which become symptomatic is 73.15 - 91.05%*
xiv. *The percentage of those requiring hospital who will require IC 0 - 30%*
xv. *The percentage of those requiring acute beds (but not IC) who will die 5 - 35%*

For peer review only

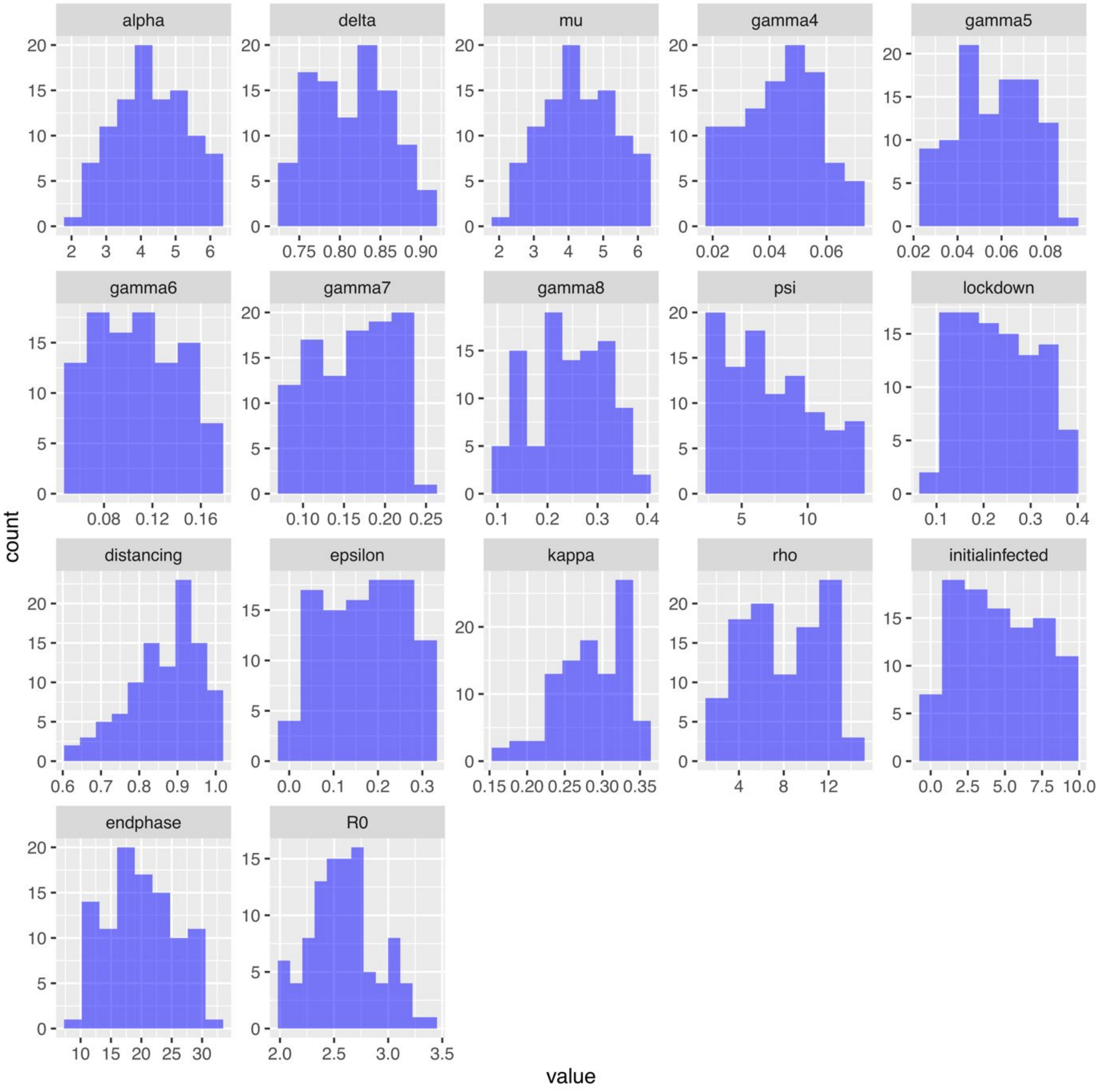

Figure S1a: The distribution of the best 100 parameters selected for in the Latin Hypercube Sampling and likelihood fitting from 100,000 simulations.

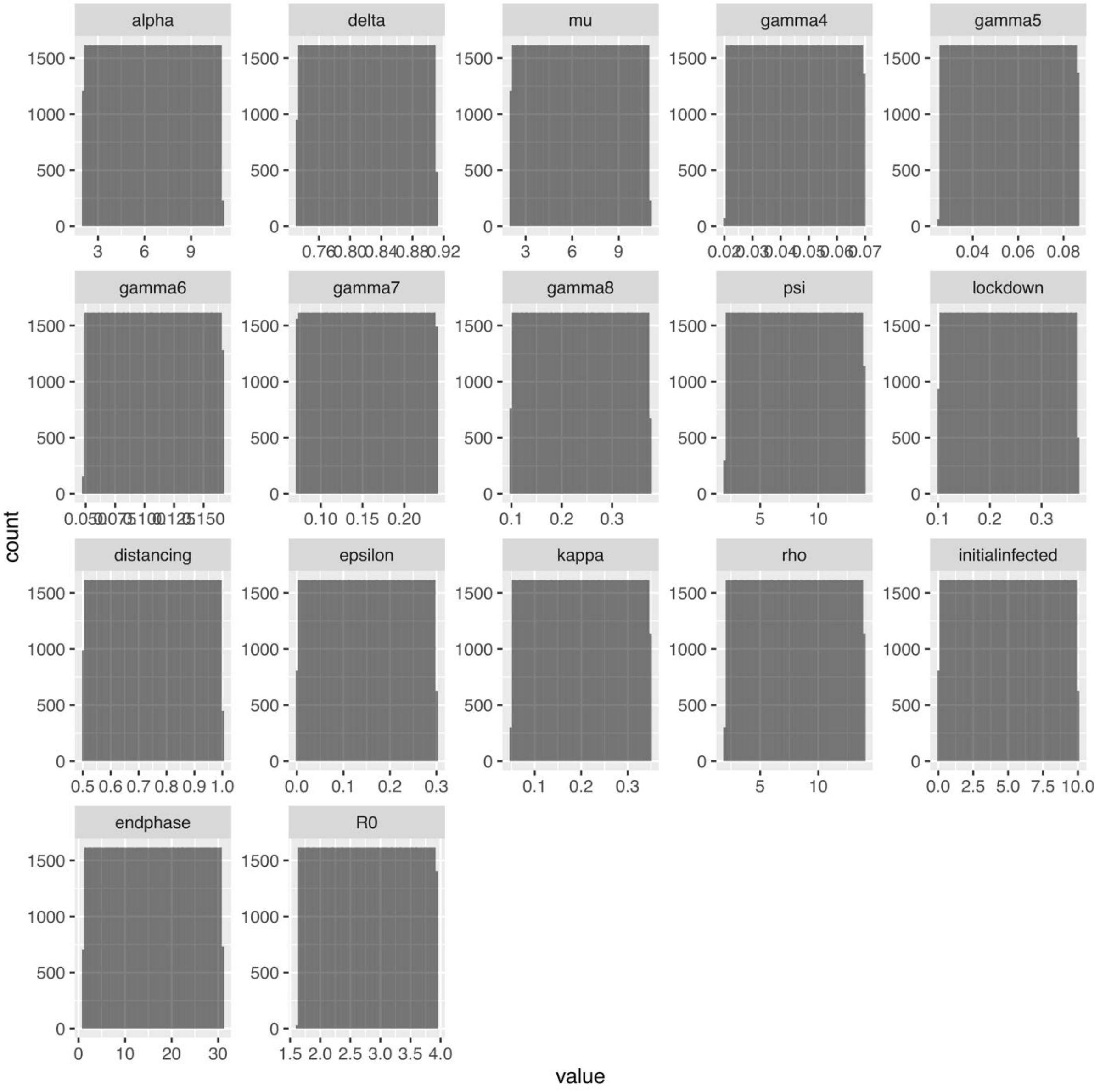

Figure S1b: The distribution of the uniform priors in the Latin Hypercube Sampling from 100,000 samples.

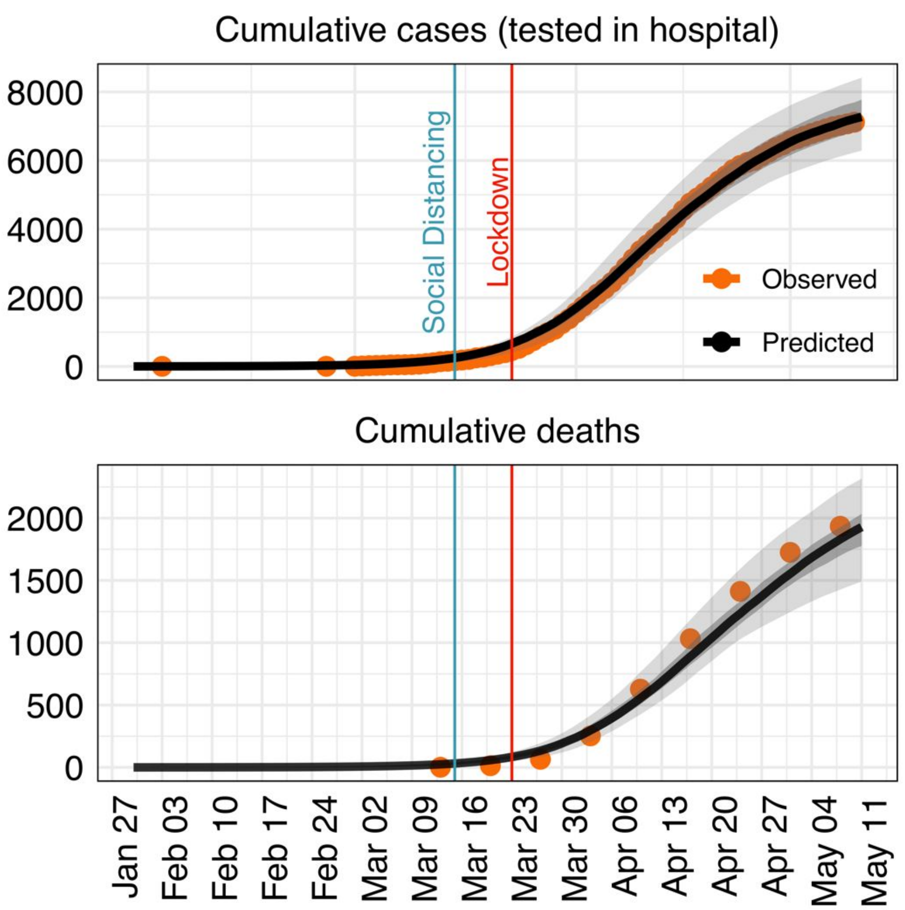

Figure S2a: Fitting performance of the model. The cumulative case numbers and hospital deaths for COVID-19 in SW England until lockdown measures were gradually lifted (11th May 2020), based on case data and death data (orange). 95% credible intervals of our model projections are shown in light grey, 50% in dark grey and the median value of the model is highlighted in black. The shaded region indicates the prediction of the model from the data. We did not consider the effects of lockdown being lifted, and our transmission rates are fitted to both before lockdown and after lockdown. Blue and red vertical lines represent the dates when social distancing and school closures/lockdown were introduced nationally, respectively.

view only

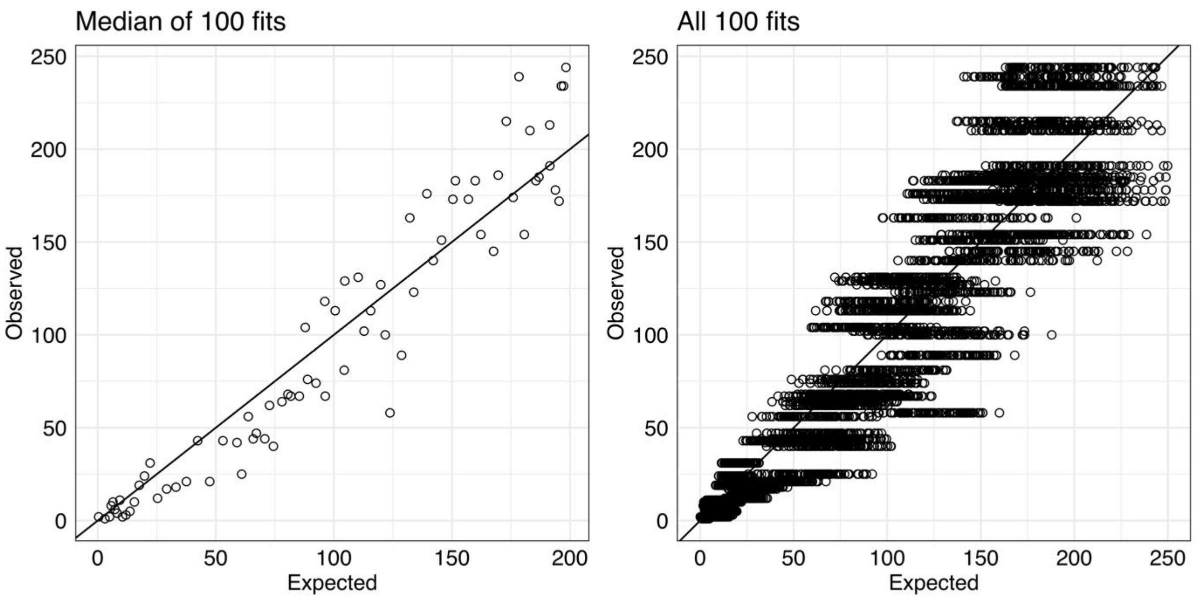

Figure S2b: The expected (model) versus observed (data) for the median of 100 fits, and all 100 fits for counts of daily case numbers. The model tends to overestimate the case data at earlier stages of the epidemic and underestimate at later stages of the epidemic.

For peer review only

Sensitivity analysis

The choice of the final sample size m=100 taken from 100k samples is based on a bias-variance trade-off with lower m giving a better model fit but higher m allowing more predictions to be included. The choice of m is not of practical significance for the median and 95% credible intervals of the main outcome measures of the model – bed modelling - as shown in the below table.

	Median (95% CrI) value on 11th May 2020			
**Size of final sample size from 100k samples**	*Total recovered*	*Total infectious*	*Total in acute beds*	*Total in IC beds*
50	191k (143k – 276k)	5k (2k – 10k)	579 (152 – 1424)	107 (14 – 497)
100	189k (142k – 278k)	6k (2k – 12k)	701 (169 – 1543)	110 (8 – 464)
200	189k (141k - 276k)	6k (2k – 16k)	709 (171 – 1564)	105 (5 – 454)
500	191k (132k – 289k)	7k (2k – 20k)	690 (171 – 1644)	109 (5 – 426)

Increasing m does slightly increase the estimate of the infectious population (*total infectious*). This is explained by including models with slightly higher R value, i.e. R is biased upwards as we move further away from the best answer. The bias is small compared to the modelling uncertainty.

	Median (95% CrI) of parameter							
**Size of final sample size from 100k samples**	R	δ	$1/\mu$	$1/\rho$	$1/\psi$	ϵ	κ	*endphase*
50	2.5 (2.1 - 3.1)	82 (74 – 90%)	3.9 (2.6 – 5.8)	6.3 (2.4 – 13.1)	6.5 (2.6 – 13.8)	19 (4 – 28%)	29 (19 – 34%)	21.3 (11.1 – 30.0)
100	2.6 (2.1 - 3.2)	82 (74 – 90%)	4.3 (2.5 – 6.0)	7.7 (2.3 – 13.3)	6.7 (2.5 – 13.8)	16 (2 – 30%)	28 (19 – 34%	19.7 (10.8 – 30.0)
200	2.6 (2.1 - 3.3)	82 (74 – 91%)	4.5 (2.4 – 6.7)	7.8 (2.3 – 13.6)	6.7 (2.4 – 13.8)	16 (1 – 30%)	28 (17 – 34%)	18.5 (8.6 – 30.3)
500	2.7 (2.1 - 3.5)	82 (74 – 90%)	5.0 (2.5 – 8.3)	7.3 (2.4 – 13.6)	7.2 (2.5 – 13.8)	17 (1 – 29%)	28 (15 – 35%)	18.3 (6.4 – 30.3)

For peer review only

The inverse of μ and ψ becomes bigger (as it is related to the selection of R), as the sample size becomes larger. This then reduces the parameters *endphase*, κ, and ϵ in order to account for this bias in R.

We choose to report values for m=100 samples as part of the bias-variance trade-off. The bias is small for this choice, with R close to unbiased and the confidence intervals capture all of the significant variation components. The qualitative inferences would not change with any of the above choices of m and uncertainty has been well captured.

For peer review only

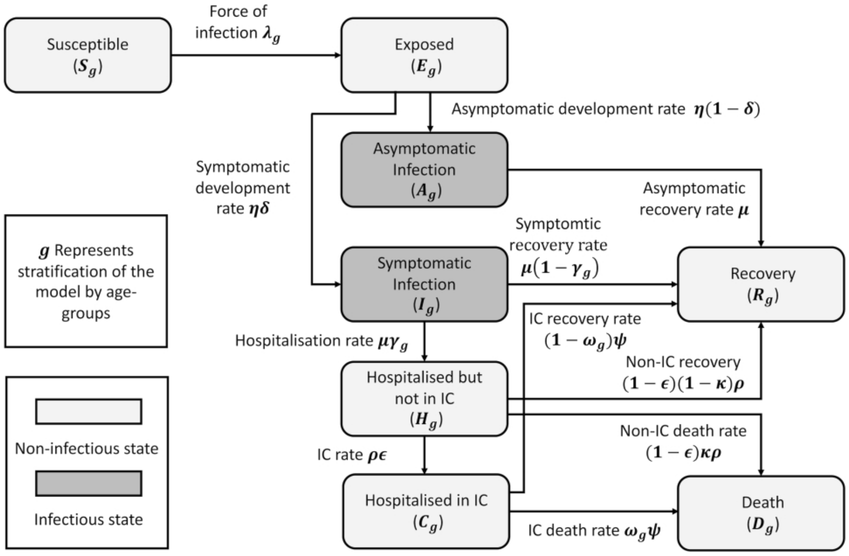

Compartmental flow model diagram depicting stages of disease and transitions between states. Asymptomatic infection represents the number of people never showing symptoms, while symptomatic infection includes all those who show pre-symptomatic / mild symptoms to those who show more severe symptoms (pre-hospitalisation). Those who are hospitalised first occupy a non-IC bed (acute bed) after which they can either move into IC, recover or die. Those in IC can either recover or die at an increased rate compared to those in acute beds. This model does not capture those deaths which occur outside of hospital as a result of COVID-19.

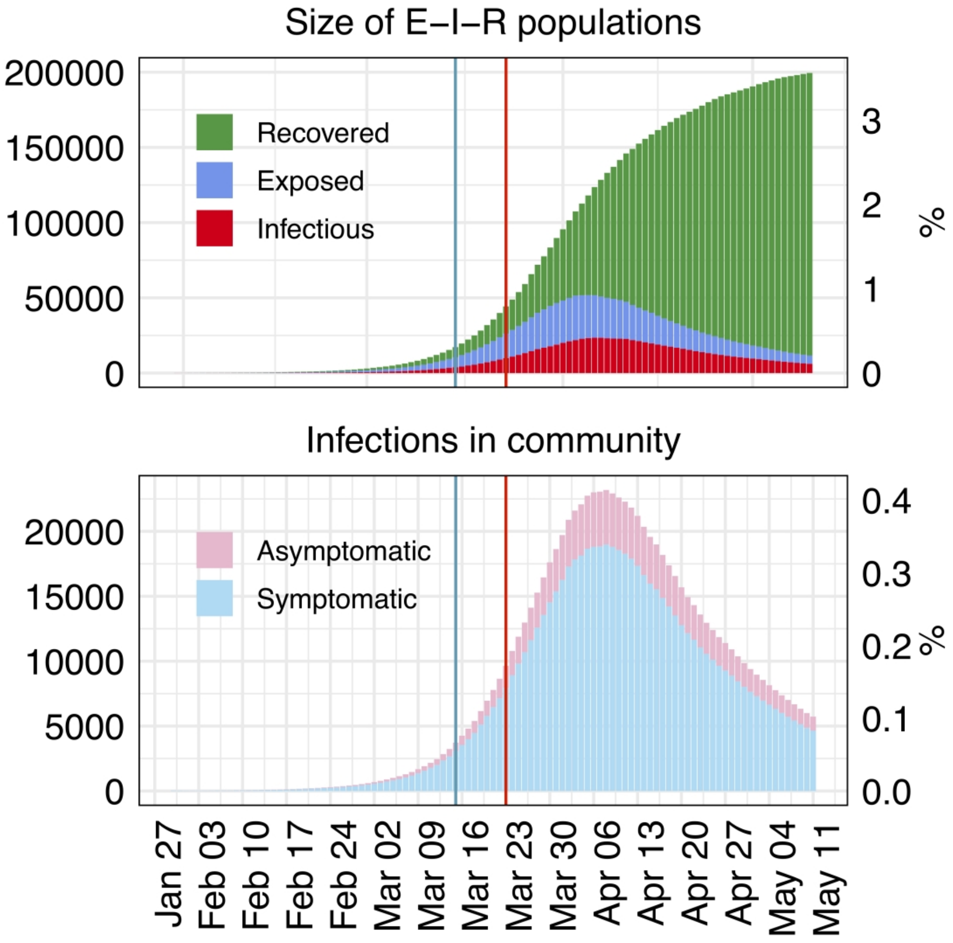

The predicted median size of the exposed (E), infectious (I) and recovered classes (R), along with the size of asymptomatic and symptomatic individuals on each day in SW England until 11th May 2020. Blue and red vertical lines represent the date the government introduced social distancing and school closures/lockdown, respectively.

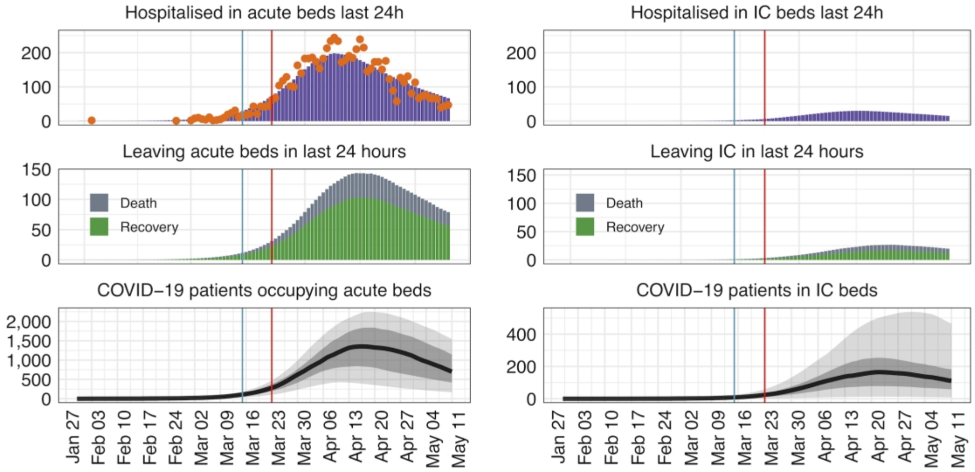

The predicted number of hospitalised patients in acute and intensive care beds in the SW until 11th May 2020. The number of daily incoming patients diagnosed with COVID-19 are shown in orange (from SW daily case data [14]), 95% credible intervals are shown in light grey, 50% in dark grey and the median value of the fits is highlighted in black. The shaded region indicates the prediction of the model from the data. Blue and red vertical lines represent the date the government introduced social distancing, and school closures/lockdown, respectively.

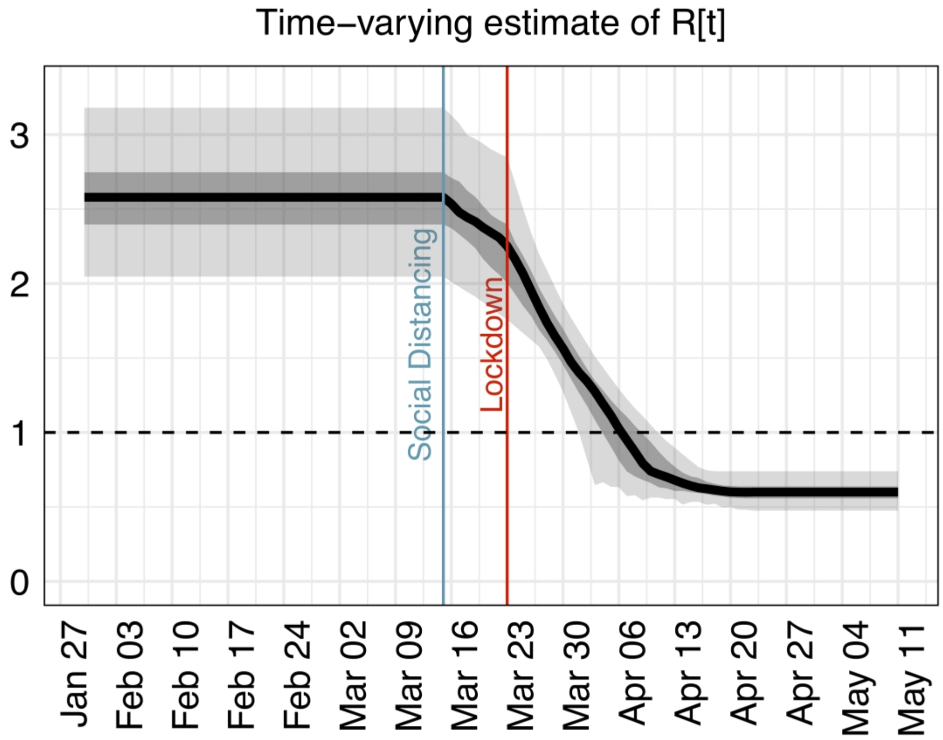

The effect of interventions on estimates of R (y-axis) over time until the 11th May 2020.

TRIPOD Checklist: Prediction Model Development

Section/Topic	Item	Checklist Item	Page
**Title and abstract**			
Title	1	Identify the study as developing and/or validating a multivariable prediction model, the target population, and the outcome to be predicted.	**1**
Abstract	2	Provide a summary of objectives, study design, setting, participants, sample size, predictors, outcome, statistical analysis, results, and conclusions.	**2**
**Introduction**			
Background and objectives	3a	Explain the medical context (including whether diagnostic or prognostic) and rationale for developing or validating the multivariable prediction model, including references to existing models.	**3**
	3b	Specify the objectives, including whether the study describes the development or validation of the model or both.	**3, 4**
**Methods**			
Source of data	4a	Describe the study design or source of data (e.g., randomized trial, cohort, or registry data), separately for the development and validation data sets, if applicable.	**11, 12, 13**
	4b	Specify the key study dates, including start of accrual; end of accrual; and, if applicable, end of follow-up.	**14**
Participants	5a	Specify key elements of the study setting (e.g., primary care, secondary care, general population) including number and location of centres.	**14**
	5b	Describe eligibility criteria for participants.	**n/a**
	5c	Give details of treatments received, if relevant.	**n/a**
Outcome	6a	Clearly define the outcome that is predicted by the prediction model, including how and when assessed.	**14**
	6b	Report any actions to blind assessment of the outcome to be predicted.	**n/a**
Predictors	7a	Clearly define all predictors used in developing or validating the multivariable prediction model, including how and when they were measured.	**12, 13, 14**
	7b	Report any actions to blind assessment of predictors for the outcome and other predictors.	**n/a**
Sample size	8	Explain how the study size was arrived at.	**12**
Missing data	9	Describe how missing data were handled (e.g., complete-case analysis, single imputation, multiple imputation) with details of any imputation method.	**12**
Statistical analysis methods	10a	Describe how predictors were handled in the analyses.	**12**
	10b	Specify type of model, all model-building procedures (including any predictor selection), and method for internal validation.	**6 - 9**
	10d	Specify all measures used to assess model performance and, if relevant, to compare multiple models.	**12, 14**
Risk groups	11	Provide details on how risk groups were created, if done.	**6**
**Results**			
Participants	13a	Describe the flow of participants through the study, including the number of participants with and without the outcome and, if applicable, a summary of the follow-up time. A diagram may be helpful.	**9**
	13b	Describe the characteristics of the participants (basic demographics, clinical features, available predictors), including the number of participants with missing data for predictors and outcome.	**6, 7**
Model development	14a	Specify the number of participants and outcome events in each analysis.	**12**
	14b	If done, report the unadjusted association between each candidate predictor and outcome.	**n/a**
Model specification	15a	Present the full prediction model to allow predictions for individuals (i.e., all regression coefficients, and model intercept or baseline survival at a given time point).	**14 - 17**
	15b	Explain how to the use the prediction model.	**6 - 9**
Model performance	16	Report performance measures (with CIs) for the prediction model.	**14 - 17**
**Discussion**			
Limitations	18	Discuss any limitations of the study (such as nonrepresentative sample, few events per predictor, missing data).	**18 - 23**
Interpretation	19b	Give an overall interpretation of the results, considering objectives, limitations, and results from similar studies, and other relevant evidence.	**18 - 23**
Implications	20	Discuss the potential clinical use of the model and implications for future research.	**23**
**Other information**			
Supplementary information	21	Provide information about the availability of supplementary resources, such as study protocol, Web calculator, and data sets.	**33 - 40**
Funding	22	Give the source of funding and the role of the funders for the present study.	**23, 24**

We recommend using the TRIPOD Checklist in conjunction with the TRIPOD Explanation and Elaboration document.

BMJ Open

Estimating the COVID-19 epidemic trajectory and hospital capacity requirements in South West England: a mathematical modelling framework

Journal:	*BMJ Open*
Manuscript ID	bmjopen-2020-041536.R1
Article Type:	Original research
Date Submitted by the Author:	16-Sep-2020
Complete List of Authors:	Booton, Ross; University of Bristol School of Veterinary Sciences<br>MacGregor, Louis; Bristol Medical School<br>Vass, Lucy; University of Bristol School of Veterinary Sciences<br>Looker, Katharine; Bristol Medical School<br>Hyams, Catherine; Academic Respiratory Unit, Southmead Hospital<br>Bright, Philip; Immunology, Pathology Sciences, North Bristol NHS Trust<br>Harding, Irasha; Consultant in Microbiology, University Hospitals Bristol<br>Lazarus, Rajeka; Consultant in Microbiology and Infectious Diseases, University Hospitals Bristol<br>Hamilton, Fergus; Infection Science, Southmead Hospital, North Bristol NHS Trust<br>Lawson, Daniel; University of Bristol School of Mathematics<br>Danon, Leon; University of Exeter College of Engineering Mathematics and Physical Sciences<br>Pratt, Adrian; Head of Modelling and Analytics, NHS Bristol, North Somerset and South Gloucestershire CCG<br>Wood, Richard; Principal BI Analyst Modelling and Analytics, NHS Bristol, North Somerset and South Gloucestershire CCG<br>Brooks-Pollock, Ellen; University of Bristol School of Veterinary Sciences; Bristol Medical School<br>Turner, Katy; University of Bristol School of Veterinary Sciences; Health Data Research UK South-West of England Partnership, Population Health Science Institute
**Primary Subject Heading**:	Public health
Secondary Subject Heading:	Epidemiology, Health informatics, Infectious diseases
Keywords:	EPIDEMIOLOGY, Public health < INFECTIOUS DISEASES, Infection control < INFECTIOUS DISEASES

SCHOLARONE™
Manuscripts

I, the Submitting Author has the right to grant and does grant on behalf of all authors of the Work (as defined in the below author licence), an exclusive licence and/or a non-exclusive licence for contributions from authors who are: i) UK Crown employees; ii) where BMJ has agreed a CC-BY licence shall apply, and/or iii) in accordance with the terms applicable for US Federal Government officers or employees acting as part of their official duties; on a worldwide, perpetual, irrevocable, royalty-free basis to BMJ Publishing Group Ltd ("BMJ") its licensees and where the relevant Journal is co-owned by BMJ to the co-owners of the Journal, to publish the Work in this journal and any other BMJ products and to exploit all rights, as set out in our licence.

The Submitting Author accepts and understands that any supply made under these terms is made by BMJ to the Submitting Author unless you are acting as an employee on behalf of your employer or a postgraduate student of an affiliated institution which is paying any applicable article publishing charge ("APC") for Open Access articles. Where the Submitting Author wishes to make the Work available on an Open Access basis (and intends to pay the relevant APC), the terms of reuse of such Open Access shall be governed by a Creative Commons licence – details of these licences and which Creative Commons licence will apply to this Work are set out in our licence referred to above.

Other than as permitted in any relevant BMJ Author's Self Archiving Policies, I confirm this Work has not been accepted for publication elsewhere, is not being considered for publication elsewhere and does not duplicate material already published. I confirm all authors consent to publication of this Work and authorise the granting of this licence.

For peer review only

Estimating the COVID-19 epidemic trajectory and hospital capacity requirements in South West England: a mathematical modelling framework

Ross D. Booton[1], Louis MacGregor[2,3], Lucy Vass[1,2], Katharine J. Looker[2,3], Catherine Hyams[4], Philip D. Bright[5], Irasha Harding[6], Rajeka Lazarus[7], Fergus Hamilton[8], Daniel Lawson[9], Leon Danon[10], Adrian Pratt[11], Richard Wood[12,13], Ellen Brooks-Pollock[1,2,3,†], Katharine M.E. Turner[1,2,3,13,†,*]

[1]Bristol Veterinary School, University of Bristol, Bristol, UK

[2]Population Health Science Institute, Bristol Medical School, University of Bristol, Bristol, UK

[3]NIHR Health Protection Research Unit in Behavioural Science and Evaluation

[4]Academic Respiratory Unit, Southmead Hospital, University of Bristol, Bristol, UK

[5]Immunology, Pathology Sciences, North Bristol NHS Trust, Bristol, UK

[6]Consultant in Microbiology, University Hospitals Bristol, Bristol, UK

[7]Consultant in Microbiology and Infectious Diseases, University Hospitals Bristol, Bristol, UK

[8]Infection Science, Southmead Hospital, North Bristol NHS Trust, Bristol, UK

[9]School of Mathematics, University of Bristol, Bristol, UK

[10]College of Engineering, Mathematics and Physical Sciences, University of Exeter, Exeter, UK

For peer review only

[11]*Principal BI Analyst Modelling and Analytics, NHS Bristol, North Somerset and South Gloucestershire CCG*

[12]*Head of Modelling and Analytics, NHS Bristol, North Somerset and South Gloucestershire CCG*

[13]*Health Data Research UK South-West of England Partnership*

†*should be considered joint senior author*

**Correspondence: katy.turner@bristol.ac.uk*

Word count: 4500 (not including references, figures or tables)

Keywords: *Mathematical model, COVID-19, hospital capacity, R value, epidemic trajectory*

Abstract

Objectives: To develop a regional model of COVID-19 dynamics, for use in estimating the number of infections, deaths and required acute and intensive care (IC) beds using the South West of England (SW) as an example case.

Design: Open-source age-structured variant of a susceptible-exposed-infectious-recovered (SEIR) compartmental mathematical model. Latin hypercube sampling and maximum likelihood estimation were used to calibrate to cumulative cases and cumulative deaths.

For peer review only

Setting: SW at a time considered early in the pandemic, where National Health Service (NHS) authorities required evidence to guide localised planning and support decision-making.

Participants: Publicly-available data on COVID-19 patients.

Primary and secondary outcome measures: The expected numbers of infected cases, deaths due to COVID-19 infection, patient occupancy of acute and IC beds and the reproduction ("R") number over time.

Results: SW model projections indicate that, as of the 11th May 2020 (when 'lockdown' measures were eased), 5,793 (95% credible interval, CrI, 2,003-12,051) individuals were still infectious (0.10% of the total SW England population, 95%CrI 0.04-0.22%), and a total of 189,048 (95%CrI 141,580-277,955) had been infected with the virus (either asymptomatically or symptomatically), but recovered, which is 3.4% (95%CrI 2.5-5.0%) of the SW population. The total number of patients in acute and IC beds in the SW on the 11th May 2020 was predicted to be 701 (95%CrI 169-1,543) and 110 (95%CrI 8-464) respectively. The R value in SW England was predicted to be 2.6 (95%CrI 2.0-3.2) prior to any interventions, with social distancing reducing this to 2.3

For peer review only

(95%CrI 1.8-2.9) and lockdown/school closures further reducing the R value to 0.6 (95CrI% 0.5-0.7).

Conclusions: The developed model has proved a valuable asset for regional healthcare services. The model will be used further in the SW as the pandemic evolves, and – as open source software – is portable to healthcare systems in other geographies.

For peer review only

Article summary

Strengths and limitations of this study:

i. Open-source modelling tool available for wider use and re-use.

ii. Customisable to a number of granularities such as at the local, regional and national level.

iii. Supports a more holistic understanding of intervention efficacy through estimating unobservable quantities, e.g. asymptomatic population.

iv. While not presented here, future use of the model could evaluate the effect of various interventions on transmission of COVID-19.

v. Further developments could consider the impact of bedded capacity in terms of resulting excess deaths.

For peer review only

Introduction

Since the initial outbreak in 2019 in Hubei Province, China, COVID-19, the disease caused by Severe Acute Respiratory Syndrome Coronavirus-2 (SARS-CoV-2), has gone on to cause a pandemic [1]. As of 11th May 2020, the Centre for Systems Science and Engineering at Johns Hopkins University reports over 4,000,000 confirmed cases and 250,000 deaths globally [2]. National responses to the outbreak have varied; from severe restrictions on human mobility alongside widespread testing and contact tracing in China [3] to the comparatively relaxed response in Sweden, where lockdown measures have not been enacted [4]. In the UK, advice to socially distance if displaying symptoms was given on the 15th March, while school closures and 'lockdown' measures were implemented from 23rd March onwards [5].

Mathematical modelling has been used to predict the course of the COVID-19 pandemic and to evaluate the effectiveness of proposed and enacted interventions [6–11]. Prem et al. [6] showed that the premature lifting of control strategies at the national level (within China) could lead to an earlier secondary peak; Flaxman et al. [7] used a semi-mechanistic model to predict the total COVID-19 infections in 11 countries;

For peer review only

Ferguson et al. [8] used an individual-based simulation model of COVID-19 transmission to explore the effects of non-pharmaceutical interventions within the USA and Great Britain; Challen et al. [9] estimated the R-number among regions of the UK; Danon et al. [10] used a spatial model to predict the potential course of COVID-19 in England and Wales in the absence of control measures; while Jarvis et al. [11] analysed the behavioural monitoring data to quantify the impact of control measures on COVID-19 transmission. These models have been predominantly aimed at the national level and have largely been based on epidemiological and biological data sourced from the initial epidemic in Wuhan, China [12] and the first large outbreak in Lombardy, Italy [13]. These models have also mainly focused on predicting the scale of COVID-19 transmission under various intervention measures, rather than producing estimates for potential numbers of COVID-19 related admissions to acute or intensive care.

In the UK, the epidemic escalated most rapidly in London [14] and the majority of national modelling is seemingly driven by the trends in London, due to its large case numbers and large population. One of the key issues facing NHS authorities is planning for more localised capacity needs and estimating the timings of surges in

For peer review only

demand at a regional or healthcare system level. This is especially challenging given the rapidly evolving epidemiological and biological data; the changes in COVID-19 testing availability (e.g. previously limited and changing eligibility requirements); the uncertainty in the effectiveness of interventions in different contexts; significant and uncertain time-lags between initial infection and hospitalisation or death; and different regions being at different points in the epidemic curve [9]. South West England (SW) is the region with the lowest number of total cases in England (as of 11th May 2020), lagging behind the national data driven by the earlier epidemic in London [9,14].

COVID-19 results in a significant requirement for hospitalisation, and high mortality amongst patients requiring admission to critical care (particularly amongst those requiring ventilation) [15,16]. In the SW, the population is on average older than in London [17] and is older than the UK as a whole (Table S1). Older age puts individuals at elevated risk of requiring hospital care [18–20]. Consequently, we might expect higher mortality and greater demand for beds in the SW than estimations output from national models that may lack such granularity or risk sensitivity.

However, the SW's first case occurred around 2 weeks later than the first UK case [14]; perhaps implying that the local SW epidemic may be more effectively

For peer review only

controlled due to a lower number of baseline cases (than the national average) at the time national interventions were implemented, as well as reduced transmission due to rurality. This sub-national analysis can support in mapping the local epidemic, planning local hospital capacity outside of the main urban centres, and ensuring effective mobilisation of additional support and resources if required. Should demand be lower than expected, reliable forecasts could facilitate more effective use of available resources through re-introducing elective treatments (that had initially been postponed) and responding to other, non-COVID-19 sources of emergency demand.

In this study, taking into account the timeline of UK-wide non-pharmaceutical interventions (social distancing, school closures/lockdown) we illustrate use of our model in projecting estimates for the expected distributions of cases, deaths, asymptomatic and symptomatic infections, and demand for acute and intensive care (IC) beds. We present the model trajectories for SW England using publicly-available data.

Methods

For peer review only

We developed a deterministic, ordinary differential equation model of the transmission dynamics of COVID-19, including age-structured contact patterns, age specific disease progression and demand for hospitalisation, both to acute and IC. We then parameterised the model using available literature and calibrated the model to data from the SW. The model is readily adapted to fit data at sub-regional (e.g. Clinical Commissioning Group), regional or national level. Key assumptions of the model are summarised in the supplementary information.

The model was developed in R and all code and links to source data are freely available (*github.com/rdbooton/bricovmod*). The model is coded using package *deSolve*, with contact matrices from package *socialmixr*, and sampling from package *lhs*.

Model structure

The stages of COVID-19 included within this model are ***S***-susceptible, ***E***-exposed (not currently infectious, but have been exposed to the virus), ***A***- asymptomatic infection (will never develop symptoms), ***I***-symptomatic infection (consisting of pre-symptomatic or mild to moderate symptoms), ***H***-severe symptoms requiring

For peer review only

hospitalisation but not IC, $\boldsymbol{C}$-very severe symptoms requiring IC, $\boldsymbol{R}$-recovered and $\boldsymbol{D}$-death. The total population is $\boldsymbol{N = S + E + A + I + H + C + R + D}$ (Figure 1).

Each compartment $\boldsymbol{X_g}$ is stratified by age-group (0-4, 5-17, 18-29, 30-39, 40-49, 50-59, 60-69, ≥70) where $\boldsymbol{X}$ denotes the stage of COVID-19 (S,E,I,A,H,C,R,D) and $\boldsymbol{g}$ denotes the age group class of individuals. Age groups were chosen to capture key social contact patterns (primary, secondary and tertiary education and employment) and variability in hospitalisation rates and outcomes from COVID-19 especially in older age groups. The total in each age group is informed by recent Office for National Statistics (ONS) estimates [21].

Susceptible individuals become exposed to the virus at a rate governed by the force of infection $\boldsymbol{\lambda_g}$, and individuals are non-infectious in the exposed category. A proportion $\boldsymbol{\delta}$ move from exposed to symptomatic infection and the remaining to asymptomatic infection, both at the latent rate $\boldsymbol{\eta}$. Individuals leave both the asymptomatic and symptomatic compartments at rate $\boldsymbol{\mu}$. All asymptomatic individuals eventually recover and there are no further stages of disease: the rate of leaving the asymptomatic compartment is therefore equivalent to the infectious period, $\boldsymbol{\mu}$. A proportion of symptomatic individuals $\boldsymbol{\gamma_g}$ go on to develop severe symptoms which

For peer review only

require hospitalisation, but not intensive care. Once requiring hospitalisation, we assume individuals are no longer infectious to the general population due to self-isolation guidelines restricting further mixing with anyone aside from household members (if unable to be admitted to hospital) or frontline NHS staff (if admitted to hospital). Individuals move out of the acute hospitalised compartment at rate $\boldsymbol{\rho}$, either through death, being moved to intensive care at rate $\boldsymbol{\epsilon}$, or through recovery (all remaining individuals). A proportion $\boldsymbol{\omega_g}$ of patients requiring IC will die at rate $\boldsymbol{\psi}$, while the rest will recover.

The model (schematic in Figure 1) is therefore described by the following differential equations:

Susceptible $\boldsymbol{S_g}$ $$\frac{dS_g}{dt} = -\lambda_g S_g \#(1a)$$

Exposed $\boldsymbol{E_g}$ $$\frac{dE_g}{dt} = \lambda_g S_g - \eta E_g \#(1b)$$

Asymptomatic $\boldsymbol{A_g}$ $$\frac{dA_g}{dt} = \eta(1-\delta)E_g - \mu A_g \#(1c)$$

Infectious $\boldsymbol{I_g}$ $$\frac{dI_g}{dt} = \eta\delta E_g - \mu I_g \#(1d)$$

Hospitalised in acute bed $\boldsymbol{H_g}$ $$\frac{dH_g}{dt} = \mu\gamma_g I_g - \rho H_g \#(1e)$$

For peer review

For peer review only - http://bmjopen.bmj.com/site/about/guidelines.xhtml

Hospitalised in IC $\boldsymbol{C_g}$

$$\frac{dC_g}{dt} = \rho\epsilon H_g - \psi C_g \#(1f)$$

Recovered $\boldsymbol{R_g}$

$$\frac{dR_g}{dt} = \mu A_g + \mu(1-\gamma_g)I_g + (1-\epsilon)(1-\kappa)\rho H_g + (1-\omega_g)\psi C_g \#(1g)$$

Death $\boldsymbol{D_g}$

$$\frac{dD_g}{dt} = (1-\epsilon)\kappa\rho H_g + \omega_g \psi C_g \#(1h)$$

181

For peer review only

Contact patterns under national interventions

We assume the population is stratified into pre-defined age groups with age-specific mixing pattern represented by a contact matrix $\boldsymbol{M}$ with an element of $\boldsymbol{m_{ij}}$ representing the contacts between someone of age group $\boldsymbol{i} \in \boldsymbol{G}$ with someone in age group $\boldsymbol{j} \in \boldsymbol{G}$. The baseline contact matrix (with no interventions in place) is taken from the POLYMOD survey conducted in the United Kingdom [22]. The contact pattern may also be influenced by a range of interventions (social distancing was encouraged on 15th March 2020, schools were closed and lockdown occurred on 23rd March 2020). We implement these interventions by assuming that the percentage of 0-18-year olds attending school after the 23rd March 2020 was 5% (reducing all contacts between school age individuals by 95%) and that social distancing reduced all contacts by 0-50%. We take the element-wise minimum for each age group's contact with another age group from all active interventions (distancing, schools/lockdown). A study on post-lockdown contact patterns (CoMix [11]) is used to inform contacts after lockdown (first survey 24th March 2020, with an average of 73% reduction in daily contacts observed per person compared to POLYMOD).

For peer review only

Moving between contact matrices of multiple interventions was implemented by assuming a phased, linear decrease. After lockdown we vary a parameter (*endphase*) to capture the time taken to fully adjust (across the population, on average) to the new measures (allowed to vary from 1 to 31 days). This assumption represents the time taken for individuals to fully adapt to new measures (and household transmission), and is in line with data on the delay in the control of COVID-19 (reductions in hospital admissions and deaths after lockdown) [23]. The parameter *endphase* can be interpreted as accounting for the time taken to adjust to all interventions (and not just lockdown).

The force of infection

The age-specific force of infection $\boldsymbol{\lambda_g}$, depends on the proportion of the population who are infectious (asymptomatic $\boldsymbol{A_g}$ and symptomatic $\boldsymbol{I_g}$ only) and probability of transmission $\boldsymbol{\beta}$:

$$\lambda_g = \beta \sum_{i \in G} m_{ig} \left(\frac{A_i}{N_g} + \frac{I_i}{N_g} \right) \#(2)$$

The basic reproduction number R_0

For peer review only

The basic reproduction number R_0 of COVID-19 is estimated to be 2.79 $\pm$ 1.16 [24]. We include this estimate within our model by calculating the maximum eigenvalue of the contact matrix $\boldsymbol{M}$, and allowing the transmission parameter to vary such that R_0 is equal to the maximum eigenvalue of $\boldsymbol{M}$ multiplied by the infectious period μ and the transmission parameter β. This gives the value for the initial basic reproduction number R_0, which changes as the contact patterns change as lockdown and other interventions are implemented.

Parameter estimates and data sources

Model parameters are detailed in Table 1. We used available published literature to inform parameter estimates. We used the following publicly-available metrics for model fitting: regional cumulative cases in SW England (tested and confirmed case in hospital), and deaths (daily/cumulative counts) from the Public Health England COVID-19 dashboard [14], and ONS weekly provisional data on COVID-19 related deaths [25]. The case data is finalised prior to the previous five days, so we include all data until 14th May 2020, based on data reported until 18th May 2020. The mortality data from ONS does not explicitly state the number of COVID-19 related deaths

For peer review only

occurring in hospital, but they do report this value nationally (83.9% of COVID-19 deaths in hospital, as of 17th April 2020). We assume that this percentage applies to the SW data and re-scale the mortality to 83.9% to represent an estimate of total deaths in hospital.

Model calibration

Using the available data (Table 1), we define ranges for all parameters in our model and sample all parameters simultaneously between these minimum and maximum values assuming uniform distributions using Latin Hypercube Sampling (statistical method for generating random parameters from multidimensional distribution), for a total of 100,000 simulations. We used maximum likelihood estimation on total cumulative cases and cumulative deaths with a Poisson negative log likelihood calculated and summed over all observed and predicted points. For i observed cases X_i (from data), and i predicted cases Y_i (from simulations of the model), we select the best 100 parameter sets which maximise the log-likelihood $\sum X_i \log(Y_i) - Y_i$ from the total sample of 100,000 simulations. The best 100 samples were taken as part of a bias-variance trade-off (Supplementary Information, Sensitivity analysis), and the

For peer review only

qualitative inferences would not change with other choices of sample size. For each data point (taken from cases and deaths) we calculate this log-likelihood, and weight each according to the square-root of the mean of the respective case or death data. This ensures that we are considering case and death data equally within our likelihood calculations.

Symbol	*Description*	*Uniform prior (min and max) or point estimate*
$\mathbf{1/\eta}$	Duration of the non-infectious exposure period	5.1 days [26]
$\boldsymbol{\delta}$	Percentage of infections which become symptomatic	82.1% [27]; Vary between 73.15 – 91.05%
$\mathbf{1/\mu}$	Duration of symptoms whilst not hospitalised (independent of outcome)	Vary between 2 – 14 days
$\mathbf{1/\rho}$	Duration of stay in acute bed (independent of outcome)	Vary between 2 – 14 days
$\boldsymbol{\gamma_g}$	Percentage of symptomatic cases which will require hospitalisation	0-4 = 0.00%, 5-17 = 0.0408%, 18-29 = 1.04%, 30-39 = 2.04-7.00%, 40-49 = 2.53-8.68%, 50-59 = 4.86-16.7%, 60-69 = 7.01-24.0%, 70+ = 9.87-37.6% [16]
$\mathbf{1/\psi}$	Duration of stay in IC bed (independent of outcome)	3 – 11 days [28]
$\boldsymbol{\epsilon}$	Percentage of those requiring hospitalisation who will require IC	Vary between 0 – 30%
$\boldsymbol{\omega_g}$	Percentage of those requiring IC who will die	0-4 = 0.00%, 5-17 = 0.00%, 18-29 = 18.1%, 30-39 = 18.1%, 40-49 = 24.7%, 50-59 = 39.3%, 60-69 = 53.9%, 70+ = 65.3% [28]

For peer review only

κ	Percentage of those requiring acute beds (but not IC) who will die	Vary between 5 – 35%
school	Percentage of 0-18-year olds attending school after 23/03/2020	Assume 5%
distancing	Percentage reduction in contact rates due to social distancing after 15/03/2020	Vary between 0 – 50%
lockdown	Percentage reduction in contact rates due to lockdown after 23/03/2020	Retail/recreation: Bristol 86% Bath 90% Plymouth 85% Gloucs 84%, Somerset 82%, Devon 85%, Dorset 84% [29]<br>Transit stations: Bristol 78% Bath 71% Plymouth 65% Gloucs 69%, Somerset 67%, Devon 66%, Dorset 63% [29]<br>Vary between 63 - 90%
R_0	Initial reproductive number of COVID-19	1.63 – 3.95 [24]
endphase	Time taken to fully adjust (across the population, on average) to new interventions	Vary between 1 – 31 days

253 **Table 1:** Parameter estimates used in the model and their sources. The distributions of unknown
254 parameters are shown in Figure S1 for the best 100 fits.

255 Model outputs

256 For each of the 100 best parameter sets we run the model until 11th May 2020 and
257 output the cumulative cases and deaths in the SW. We output the predicted proportion
258 of the population who are infectious and who have ever been infected over time.
259 Finally, we estimate the daily and cumulative patterns of admission to and discharge

For peer review only

from hospital (intensive care and acute) and cumulative mortality from COVID-19. We perform sensitivity analysis on the performance of the model when calibrated to subsets of the full data.

Results and outputs

From 100,000 simulated parameter sets, we selected the best 100 baseline model fits on the basis of agreement to the calibration data on daily confirmed COVID-19 cases and weekly mortality due to COVID-19 in SW England. The distribution of the best fitting values are shown in Figure S1a. All results are shown with median and 95% credible intervals (95%CrI).

On 11th May 2020, the reported cumulative number of individuals with (lab confirmed) COVID-19 was 7,116 in SW England [14], and the most recent report on total cumulative deaths showed that 2,306 had died from COVID-19 (as of 8th May 2020) [25].

For peer review only

Estimating the total proportion of individuals in South West England with COVID-19

Figure 2 shows the projected numbers of exposed, recovered and infectious (asymptomatic and symptomatic infections) until lockdown measures were lessened on the 11th May 2020. On this date, the model predicts that a total of 5,793 (95%CrI 2,003 – 12,051) were infectious (0.10% of the total SW England population, 95%CrI 0.04 – 0.22%). The model also predicts that a total of 189,048 (95%CrI 141,580 – 277,955) have had the virus but recovered (either asymptomatically or symptomatically), which is 3.4% (95%CrI 2.5 – 5.0%) of the SW population (not infectious and not susceptible to reinfection).

Estimating the total COVID-19 hospitalised patients in acute and intensive care beds

The total number of patients in acute (non-intensive care) hospital beds across SW England was projected to be 701 (95%CrI 169 – 1,543) and the total number of patients in intensive care hospital beds was projected to be 110 (95%CrI 8 – 464) on

For peer review only

the 11th May 2020 (Figure 3). Note that these ranges are quite large due to the uncertainty in the data and as more data becomes available these predictions will change.

Estimating the reproduction number under interventions

Figure 4 shows the model prediction for the reproduction ("R") number over time until 11th May 2020, when lockdown measures were relaxed. All interventions (social distancing, school closures/lockdown) had a significant impact on the reproductive number for COVID-19 in the South West of England. We predict that prior to any interventions R was 2.6 (95%CrI 2.0 – 3.2), and the introduction of social distancing reduced this number to 2.3 (95%CrI 1.8 – 2.9). At the minimum, R was 0.6 (95%CrI 0.5 – 0.7) after all prior interventions were enacted and adhered to (social distancing, school closures and lockdown).

Additional results for the fitting performance of the model (Figure S2a, S2b, Table S2), the performance based on prior data (Figure S3a, S3b, S3c, S3d) and sensitivity analysis can be found in the supplementary information.

For peer review only

Discussion

We have developed a deterministic ordinary differential equation model of the epidemic trajectory of COVID-19 focussing on acute and IC hospital bed capacity planning to support local NHS authorities, calibrating to SW-specific data. The model is age-structured and includes time-specific implementation of current interventions (following advice and enforcement of social distancing, school closures and lockdown) to predict the potential range of COVID-19 epidemic trajectories.

Using the publicly-available data on cases and deaths, combined with the early estimates of parameters from early epidemics in other settings, we predict that on the 11th May 2020 a total of 5,793 (95%CrI 2,003 – 12,051) were infectious, which equates to 0.10% (95%CrI 0.04 – 0.22%) of the total SW population. In addition, we find that the model predicts a total of 189,048 (95%CrI 141,580 – 277,955) have had the virus but recovered, which is 3.4% (95%CrI 2.5 – 5.0%) of the SW population.

We also estimate that the total number of patients in acute hospital beds in SW England on 11th May 2020 was 701 (95%CrI 169 – 1,543) and in IC were 110 (95%CrI

For peer review only

8 – 464), while the R number has decreased from 2.6 (95%CrI 2.0 – 3.2) to 0.6 (95%CrI 0.5 – 0.7) after all interventions were enacted and fully adhered to.

The fits generally agree well with both the daily case data, and the cumulative count of deaths in the SW, although the model overestimates the case data at early stages, and underestimates later on (which can see seen in Figure S2a, and a scatter plot of expected versus observed outputs in Figure S2b). This could be because we are using formal fitting methods, or from the under-reporting of cases in the early epidemic. When assessing model performance by projecting the numbers of cases and deaths forward from four dates in April, the model performs reasonably well, with more reliable predictions occurring when more data is used to fit the model (Figures S3a, S3b, S3c and S3d). Even when using around half of the available data to generate forecasts (Figure S3d), the model performs reasonably well and captures the observed data later in May, but overestimates case numbers and underestimates deaths similar to those in the main analysis and in Figure S2a. This suggests that our model could perform reasonably well at predicting COVID-19 outcomes but may still slightly overestimate case numbers and underestimate deaths.

For peer review only

The primary strength of this study is that we have developed generalisable and efficient modelling code incorporating disease transmission, interventions and hospital bed demand which can be adapted for use in other regional or national scenarios, with the model available on GitHub for open review and use (*github.com/rdbooton/bricovmod*). We have worked closely with the NHS and at Clinical Commissioning Group (CCG) level to ensure the model captures key clinical features of disease management in SW hospitals and provides output data in a format relevant to support local planning. We combined local clinical expertise with detailed literature searches, to ensure reasonable parameter ranges and assumptions in the presence of high levels of parameter uncertainty.

The main challenge of this work is in balancing the urgent need locally for prediction tools which are up to date (i.e. not relying on the national trends to inform capacity planning), versus more exhaustive and robust methods for model comparison. The latter of which uses existing models and more time-consuming (but more robust) data fitting methods [30,31]. However, we believe that release of this paper and sharing of model code will facilitate multidisciplinary collaboration, rapid review and support future model comparison and uncertainty analyses [31].

For peer review only

As with all models of new infections there are significant parameter uncertainties. Rapidly emerging literature is exploring a wide range of biological and epidemiological factors concerning COVID-19, but due to the worldwide nature of these studies, often parameter bands are wide and may be context specific. For example, early estimates of the basic reproduction number ranged from 1.6 to 3.8 in different locations [32,33], with an early estimate of 2.4 used in UK model projections [8]. In addition, the information which informs our parameter selection is rapidly evolving as new data is made available, sometimes on a daily basis. From our initial analysis, we identified the following parameters as critical in determining the epidemic trajectory within our model – the percentage of infections which become symptomatic, the recovery time for cases which do not require hospital, the period between acute and IC occupancy, the length of stay in IC, the probability of transmission per contact and the gradual implementation of lockdown rather than immediate effect. Other parameters (such as the percentage reduction in school-age contacts from school closures) did not seem to influence the dynamic trajectory as strongly – and thus we assume point estimates for these parameters. However, for example assuming that 95% of school-age contacts are reduced as a direct result of school closures is

For peer review only

375 perhaps an overestimate, and future modelling work should address these
376 uncertainties and their impacts on the epidemic trajectory of COVID-19 (but in this
377 case, this value was somewhat arbitrary, and the assumption was used in the absence
378 of school-age contact survey data). In addition, we did not explicitly model the societal
379 effect prior to governmental advice (social distancing, school closures, lockdown),
380 instead assuming a fixed date, before which we assume there was no interventions.
381 This assumption may not be realistic and could have influenced the model output, but
382 it is difficult to quantify the percentage compliance with interventions prior to the official
383 release of governmental advice. More research is urgently needed to refine these
384 parameter ranges and to validate these biological parameters experimentally. These
385 estimates will improve the model as more empirical data become available. We look
386 forward to reducing the uncertainty in these parameters so that we can make better
387 predictions and fit the data more accurately.

388 We have also assumed that there is no nosocomial transmission of infection
389 between hospitalised cases and healthcare workers, as we do not have good data for
390 within-hospital transmission. However frontline healthcare staff were likely to have
391 been infected early on in the epidemic [34], which could have implications for our

For peer review only

predicted epidemic trajectory. Our model also assumes a closed system, which may not strictly be true due to continuing essential travel. But given that up until 11th May, travel restrictions are very severe due to lockdown measures [5], any remaining inter-regional travel is likely to have minimal effects on our model outputs. In addition, we assume that the transmission dynamics of asymptomatic individuals is equal to those of symptomatic individuals, due to the viral load of asymptomatic and symptomatic carriers being comparable [35]. However, this assumption should be further explored in future modelling studies due to the potential for asymptomatic carriers to engage in higher-risk behaviour, and potentially impact the transmission dynamics of COVID-19.

Similar to most other COVID-19 models, we use a variant on a susceptible-exposed-infectious-recovered (SEIR) structure [8–10,16,30,36,37]. We do not spatially structure the population as in other UK modelling [9,10], but we do include age-specific mixing based on POLYMOD data [22], and the post-lockdown CoMix study [11]. We also explicitly measure the total asymptomatic infection, and the total in each of the clinically relevant hospital classes (acute or IC), which is a strength of our approach. Future models could also take into account local bed capacity within hospitals (including Nightingale centres) and accommodate the effect of demand

For peer review only

outstripping supply leading to excess deaths, inclusive of non-hospital-based death such as is occurring within care homes. Future models should also address the way in which we have compartmentalised the flow of hospitalisation and death. From the symptomatic compartment, patients either recover or are admitted to hospital, from where they either die, recover or progress to IC. Under our assumption, the symptomatic recovery rate is equal to the hospitalisation rate, and the time taken for acute patients to move to IC is equal to the time to discharge for acute patients. These assumptions are a limitation of our model, because in reality those patients who progress to IC may have spent very little time in an acute bed (either due to rapid deterioration or presenting with severe symptoms). Future studies should assess the effects of these assumptions and consider other such progressions and outcomes for a COVID-19 patient through the hospital. As with all modelling, we have not taken into account all possible sources of modelling misspecification. Some of these misspecifications will tend to increase the predicted epidemic period, and others will decrease it. One factor that could significantly change our predicted epidemic period is the underlying structure within the population leading to heterogeneity in the average number of contacts under lockdown e.g. key workers have high levels of contact but

For peer review only

others are able to minimise contacts effectively, this might lead to an underestimate of ongoing transmission, but potentially an overestimate of the effect of releasing lockdown. We also know that there are important socio-economic considerations in determining people's ability to stay at home and particularly to work from home [38].

Early UK modelling predicted the infection peak to be reached roughly 3 weeks from the initiation of severe lockdown measures, as taken by the UK government in mid-March [8]. A more recent study factoring spatial distribution of the population indicated the peak to follow in early April due to R_0 reducing to below 1 in many settings in weeks following lockdown [9]. Other modelling indicated that deaths in the UK would peak in mid-late April; furthermore, that the UK would not have enough acute and IC beds to meet demand [39]. While modelling from the European Centre for Disease Prevention and Control estimated peak cases to occur in most Euopean countries in mid-April [20], these estimations were largely at a national level. Due to the expected lag of other regions behind London, these estimated peaks are likely to be shifted further into the future for the separate regions of the UK, and as shown by our model occurred in early- to mid-April. This is also likely to be true for future peaks which may result from relaxing lockdown.

For peer review only

443 Outside of the UK, similar modelling from France [36] (which went into lockdown
444 at a similar time the UK on 17th March), predicted the peak in daily IC admissions at
445 the end of March. Interestingly however, when dissected by region, the peak in IC bed
446 demand varied by roughly 2 weeks. Swiss modelling similarly predicted a peak in
447 hospitalisation and numbers of patients needing IC beds in early April, after lockdown
448 implementation commenced on 17th March [37]. US modelling [40] disaggregated by
449 State, also highlights the peak of excess bed demand varies geographically, with this
450 peak ranging between the 2nd week of April, through to May dependent on the State
451 under consideration. The modelling based in France also cautioned that due to only
452 5.7% of the population having been infected by 11th May when the restrictions would
453 be eased, the population would be vulnerable to a second epidemic peak thereafter
454 [36].

455 The ONS in England estimated that an average of 0.25% of the population had
456 COVID-19 between the 4th and 17th May 2020 (95% confidence interval: 0.16 – 0.38%)
457 [41], which is greater than the 0.10% (95%CrI 0.04 – 0.22%) we found with our model
458 (on 11th May 2020), but with some overlap. In addition, the ONS estimated that 6.78%
459 (95%CrI 5.21 – 8.64%) tested positive for antibodies to COVID-19 up to 24th May 2020

For peer review only

in England [42], and Public Health England estimated that approximately 4% (2 – 6%) tested positive for antibodies to COVID-19 between the 20th and 26th April 2020 in the SW [43]. Comparing to our model, 3.4% (95%CrI 2.5 – 5.0%) had recovered on the 11th May 2020 (2 weeks later), demonstrating that our model estimates may be within sensible bounds, and further highlighting the need for more regional estimates of crucial epidemiological parameters and seroprevalence. We have assumed that individuals are not susceptible to reinfection within the model timeframe, however in future work it will be important to explore this assumption. It is not known what the long term pattern of immunity to COVID-19 will be [44], and this will be key to understanding the future epidemiology in the absence of a vaccine or effective treatment options.

With this in mind, our findings demonstrate that there are still significant data gaps – and in the absence of such data, mathematical models can provide a valuable asset for local and regional healthcare services. This regional model will be used further in the SW as the pandemic evolves and could be used within other healthcare systems in other geographies to support localised predictions. Controlling intervention measures at a more local level could be made possible through monitoring and

For peer review only

assessment at the regional level through a combination of clinical expertise and local policy, guided by localised predictive forecasting as presented in this study.

Funding statement

This work was supported by Global Public Health strand of the Elizabeth Blackwell Institute for Health Research, funded under the University of Bristol's QR GCRF strategy (award number ISSF3: 204813/Z/16/Z). This work was also funded with support from Bristol UNCOVER (Bristol COVID Emergency Research, award number also ISSF3: 204813/Z/16/Z) and Medical Research Council UK (award number MR/S004769/1). LM, KJL, EBP and KMET acknowledge support from the NIHR Health Protection Research Unit in Behavioural Science and Evaluation at the University of Bristol (award number NIHR200877).

This work was also supported by Health Data Research UK, which is funded by the UK Medical Research Council, Engineering and Physical Sciences Research Council, Economic and Social Research Council, National Institute for Health Research, Chief Scientist Office of the Scottish Government Health and Social Care

For peer review only

Directorates, Health and Social Care Research and Development Division (Welsh Government), Public Health Agency (South Western Ireland), British Heart Foundation and Wellcome (award number CFC0129).

Competing interests statement

We declare no competing interests.

Author contributions

Conception and design of the study: LD, EBP. Acquisition of data: RDB, EBP, RW, KMET. Mathematical modelling: RDB, LM, LD, EBP, RW, KMET. Coding and simulations: RDB. Analysis and interpretation of results: RDB, LM, LV, KJL, CH, PB, IH, RL, FH, DL, LD, AP, EBP, RW, KMET. Writing and drafting of the manuscript: RDB, LM, LV, KJL, CH, PB, IH, RL, FH, DL, LD, AP, EBP, RW, KMET. Approval of the submitted manuscript: RDB, LM, LV, KJL, CH, PB, IH, RL, FH, DL, LD, AP, EBP, RW, KMET.

For peer review only

Data sharing statement

All model code is open source and available for download on GitHub https://github.com/rdbooton/bricovmod. All data is freely available via the GOV.UK COVID-19 dashboard [14] and ONS [21,25].

Ethics and dissemination

No ethical approval was required for this mathematical modelling work.

Patient and Public Involvement

This research was done without patient involvement. Patients were not invited to comment on the study design and were not consulted to develop patient relevant outcomes or interpret the results. Patients were not invited to contribute to the writing or editing of this document for readability or accuracy.

Figure Legends:

For peer review only

Figure 1: Compartmental flow model diagram depicting stages of disease and transitions between states. Asymptomatic infection represents the number of people never showing symptoms, while symptomatic infection includes all those who show pre-symptomatic / mild symptoms to those who show more severe symptoms (pre-hospitalisation). Those who are hospitalised first occupy a non-IC bed (acute bed) after which they can either move into IC, recover or die. Those in IC can either recover or die at an increased rate compared to those in acute beds. This model does not capture those deaths which occur outside of hospital as a result of COVID-19.

Figure 2: The predicted median size of the exposed (E), infectious (I) and recovered classes (R), along with the size of asymptomatic and symptomatic individuals on each day in SW England until 11th May 2020. Blue and red vertical lines represent the date the government introduced social distancing and school closures/lockdown, respectively.

Figure 3: The predicted number of hospitalised patients in acute and intensive care beds in the SW until 11th May 2020. The number of daily incoming patients diagnosed with COVID-19 are shown in orange (from SW daily case data [14]), 95% credible intervals are shown in light grey, 50% in dark grey and the median value of the fits is

For peer review only

highlighted in black. The shaded region indicates the prediction of the model from the data. Blue and red vertical lines represent the date the government introduced social distancing, and school closures/lockdown, respectively.

Figure 4: The effect of interventions on estimates of R (y-axis) over time until the 11th May 2020.

References

1. World Health Organization. WHO Director-General's opening remarks at the mission briefing on COVID-19. 2020 [cited 27 Apr 2020]. Available: https://www.who.int/dg/speeches/detail/who-director-general-s-opening-remarks-at-the-media-briefing-on-covid-19---11-march-2020

2. Johns Hopkins University. COVID-19 Data Center. COVID-19 Case Tracker. [cited 27 Apr 2020]. Available: https://coronavirus.jhu.edu/

3. Payne C. State intervention in China. Nat Hum Behav. 2020;1.

For peer review only

4. Public Health Agency of Sweden. Public Health Authority regulations and general advice on everyone's responsibility to prevent the infection of COVID-19. 2020 [cited 7 Apr 2020]. Available: https://www.folkhalsomyndigheten.se/publicerat-material/publikationsarkiv/h/hslf-fs-202012/

5. Public Health England. Guidance on social distancing for everyone in the UK. In: Guidance on social distancing for everyone in the UK [Internet]. 2020 [cited 27 Apr 2020]. Available: https://www.gov.uk/government/publications/covid-19-guidance-on-social-distancing-and-for-vulnerable-people/guidance-on-social-distancing-for-everyone-in-the-uk-and-protecting-older-people-and-vulnerable-adults

6. Prem K, Liu Y, Russell TW, Kucharski AJ, Eggo RM, Davies N, et al. The effect of control strategies to reduce social mixing on outcomes of the COVID-19 epidemic in Wuhan, China: a modelling study. Lancet Public Heal. 2020.

7. Flaxman S, Mishra S, Gandy A, Unwin JT, Coupland H, Mellan TA, et al. Estimating the number of infections and the impact of non-pharmaceutical interventions on COVID-19 in 11 European countries. Imp Coll London

For peer review only

COVID-19 Report No 13. 2020.

8. Ferguson NM, Laydon D, Gemma Nedjati-Gilani, Natsuko Imai, Kylie Ainslie MB, Sangeeta Bhatia, Adhiratha Boonyasiri, Zulma Cucunubá, Gina Cuomo-Dannenburg, Amy Dighe I, Dorigatti, Han Fu, Katy Gaythorpe, Will Green, Arran Hamlet, Wes Hinsley, Lucy C Okell S van, Elsland, Hayley Thompson, Robert Verity, Erik Volz, Haowei Wang, Yuanrong Wang PGW, et al. Impact of non-pharmaceutical interventions (NPIs) to reduce COVID19 mortality and healthcare demand. Imp Coll London COVID-19 Report No 9. 2020.

9. Challen R, Tsaneva-Atanasova K, Pitt M. Estimates of regional infectivity of COVID-19 in the United Kingdom following imposition of social distancing measures. medRxiv. 2020.

10. Danon L, Brooks-Pollock E, Bailey M, Keeling MJ. A spatial model of CoVID-19 transmission in England and Wales: early spread and peak timing. medRxiv. 2020.

11. Jarvis CI, Zandvoort K Van, Gimma A, Prem K, Group CC-19 working, Klepac P, et al. Quantifying the impact of physical distance measures on the transmission of COVID-19 in the UK. medRxiv. 2020.

For peer review only

12. The Novel Coronavirus Pneumonia Emergency Response Epidemiology Team. The epidemiological characteristics of an outbreak of 2019 novel coronavirus diseases (COVID-19) — China, 2020. Chin J Epidemiol. 2020;41.

13. Onder G, Rezza G, Brusaferro S. Case-fatality rate and characteristics of patients dying in relation to COVID-19 in Italy. JAMA - Journal of the American Medical Association. 2020.

14. Public Health England. Coronavirus (COVID-19) in the UK: data dashboard. 2020 [cited 28 Apr 2020]. Available: https://coronavirus.data.gov.uk/#

15. Yang X, Yu Y, Xu J, Shu H, Xia J, Liu H, et al. Clinical course and outcomes of critically ill patients with SARS-CoV-2 pneumonia in Wuhan, China: a single-centered, retrospective, observational study. Lancet Respir Med. 2020.

16. Verity R, Okell LC, Dorigatti I, Winskill P, Whittaker C, Imai N, et al. Estimates of the severity of coronavirus disease 2019 : a model-based analysis. Lancet Infect Dis. 2020.

17. McCurdy C. Ageing, fast and slow: when place and demography collide. Resolut Found. 2019. Available: https://wwwresolutionfoundationorg/app/uploads/2019/10/Ageing-fast-and-

For peer review only

slowpdf

18. Yang J, Zheng Y, Gou X, Pu K, Chen Z, Guo Q, et al. Prevalence of comorbidities in the novel Wuhan coronavirus (COVID-19) infection: a systematic review and meta-analysis. Int J Infect Dis. 2020.

19. Zhou F, Yu T, Du R, Fan G, Liu Y, Liu Z, et al. Clinical course and risk factors for mortality of adult inpatients with COVID-19 in Wuhan, China: a retrospective cohort study. Lancet. 2020.

20. Stockholm: ECDC (European Centre for Disease Prevention and Control). Coronavirus disease 2019 (COVID-19) pandemic: increased transmission in the EU/EEA and the UK – seventh update, 25 March 2020. 2020. Available: https://www.ecdc.europa.eu/sites/default/files/documents/RRA-seventh-update-Outbreak-of-coronavirus-disease-COVID-19.pdf

21. Office for National Statistics. Estimates of the population for the UK, England and Wales, Scotland and Northern Ireland. 2020. Available: https://www.ons.gov.uk/peoplepopulationandcommunity/populationandmigration/populationestimates/datasets/populationestimatesforukenglandandwalesscotlandandnorthernireland

For peer review only

22. Mossong J, Hens N, Jit M, Beutels P, Auranen K, Mikolajczyk R, et al. Social contacts and mixing patterns relevant to the spread of infectious diseases. PLoS Med. 2008. doi:10.1371/journal.pmed.0050074

23. Stedman M, Davies M, Anderson SG, Lunt M, Verma A, Heald A. A phased approach to unlocking during the COVID-19 pandemic; Lessons from trend analysis. medRxiv. 2020. doi:10.1101/2020.04.20.20072264

24. Liu Y, Gayle AA, Wilder-Smith A, Rocklöv J. The reproductive number of COVID-19 is higher compared to SARS coronavirus. J Travel Med. 2020;27. doi:10.1093/jtm/taaa021

25. Office for National Statistics. Deaths registered in England and Wales, provisional 2020: up to week ending 17 April 2020. 2020. Available: https://www.ons.gov.uk/peoplepopulationandcommunity/birthsdeathsandmarriages/deaths/datasets/weeklyprovisionalfiguresondeathsregisteredinenglandandwales

26. Lauer SA, Grantz KH, Bi Q, Jones FK, Zheng Q, Meredith HR, et al. The incubation period of coronavirus disease 2019 (COVID-19) from publicly reported confirmed cases: estimation and application. Ann Intern Med. 2020.

For peer review only

27. Mizumoto K, Kagaya K, Zarebski A, Chowell G. Estimating the asymptomatic proportion of coronavirus disease 2019 (COVID-19) cases on board the Diamond Princess cruise ship, Yokohama, Japan, 2020. Eurosurveillance. 2020.

28. ICNARC COVID-19 study case mix programme database. ICNARC report on COVID-19 in critical care 15 May 2020. 2020. Available: https://www.icnarc.org/Our-Audit/Audits/Cmp/Reports

29. Google. United Kingdom COVID-19 Community Mobility Report: Mobility changes April 5 2020. 2020. Available: https://www.gstatic.com/covid19/mobility/2020-04-05_GB_Mobility_Report_en.pdf

30. Wynants L, Van Calster B, Bonten MMJ, Collins GS, Debray TPA, De Vos M, et al. Prediction models for diagnosis and prognosis of covid-19 infection: systematic review and critical appraisal. BMJ. 2020.

31. Latif S, Usman M, Manzoor S, Iqbal W, Qadir J, Tyson G, Castro I, Razi A, Boulos MN CJ. Leveraging Data Science To Combat COVID-19: A Comprehensive Review. 2020.

For peer review only

32. Kucharski AJ, Russell TW, Diamond C, Liu Y, Edmunds J, Funk S, et al. Early dynamics of transmission and control of COVID-19: a mathematical modelling study. Lancet Infect Dis. 2020.

33. Read JM, Bridgen JRRE, Cummings DATA, Ho A, Jewell CP. Novel coronavirus 2019-nCoV: early estimation of epidemiological parameters and epidemic predictions. medRxiv. 2020.

34. The Centre for Evidence-Based Medicine Website. How many healthcare workers are infected? 2020 [cited 23 Apr 2020]. Available: https://wwwcebmnet/covid-19/covid-19-how-many-healthcare-workers-are-infected/

35. Huff H V, Singh A. Asymptomatic transmission during the COVID-19 pandemic and implications for public health strategies. Clin Infect Dis. 2020; ciaa654. doi:10.1093/cid/ciaa654

36. Salje H, Kiem C, Lefrancq N, Courtejoie N, Bosetti P. Estimating the burden of SARS-CoV-2 in France. HAL ID pasteur-02548181. 2020.

37. Althaus C. Real-time modeling and projections of the COVID-19 epidemic in Switzerland. Inst Soc Prev Med Univ Bern, Switz. 2020. Available:

For peer review only

https://ispmberngithubio/covid-19/swiss-epidemic-model/

38. Dingel J, Neiman B. How Many Jobs Can be Done at Home? NBER Work Pap. 2020.

39. Institute for Health Metrics and Evaluation. New COVID-19 forecasts for Europe: Italy & Spain have passed the peak of their epidemics; UK, early in its epidemic, faces a fast-mounting death toll. 2020. Available: http://www.healthdata.org/news-release/new-covid-19-forecasts-europe-italy-spain-have-passed-peak-their-epidemics-uk-early-its

40. IHME COVID-19 health service utilization forecasting team. Forecasting COVID-19 impact on hospital bed-days, ICU-days, ventilator-days and deaths by US state in the next 4 months. medxriv. 2020.

41. ONS. Coronavirus (COVID-19) Infection Survey pilot: England, 21 May 2020. 2020.

42. ONS. Coronavirus (COVID-19) Infection Survey pilot: 28 May 2020. 2020.

43. Public Health England. Weekly Coronavirus Disease 2019 (COVID-19) Surveillance Report, Week 22. 2020. Available: https://assets.publishing.service.gov.uk/government/uploads/system/uploads/a

For peer review only

ttachment_data/file/888254/COVID19_Epidemiological_Summary_w22_Final.pdf

44. Altmann DM, Douek DC, Boyton RJ. What policy makers need to know about COVID-19 protective immunity. The Lancet Comments. 2020.

For peer review only

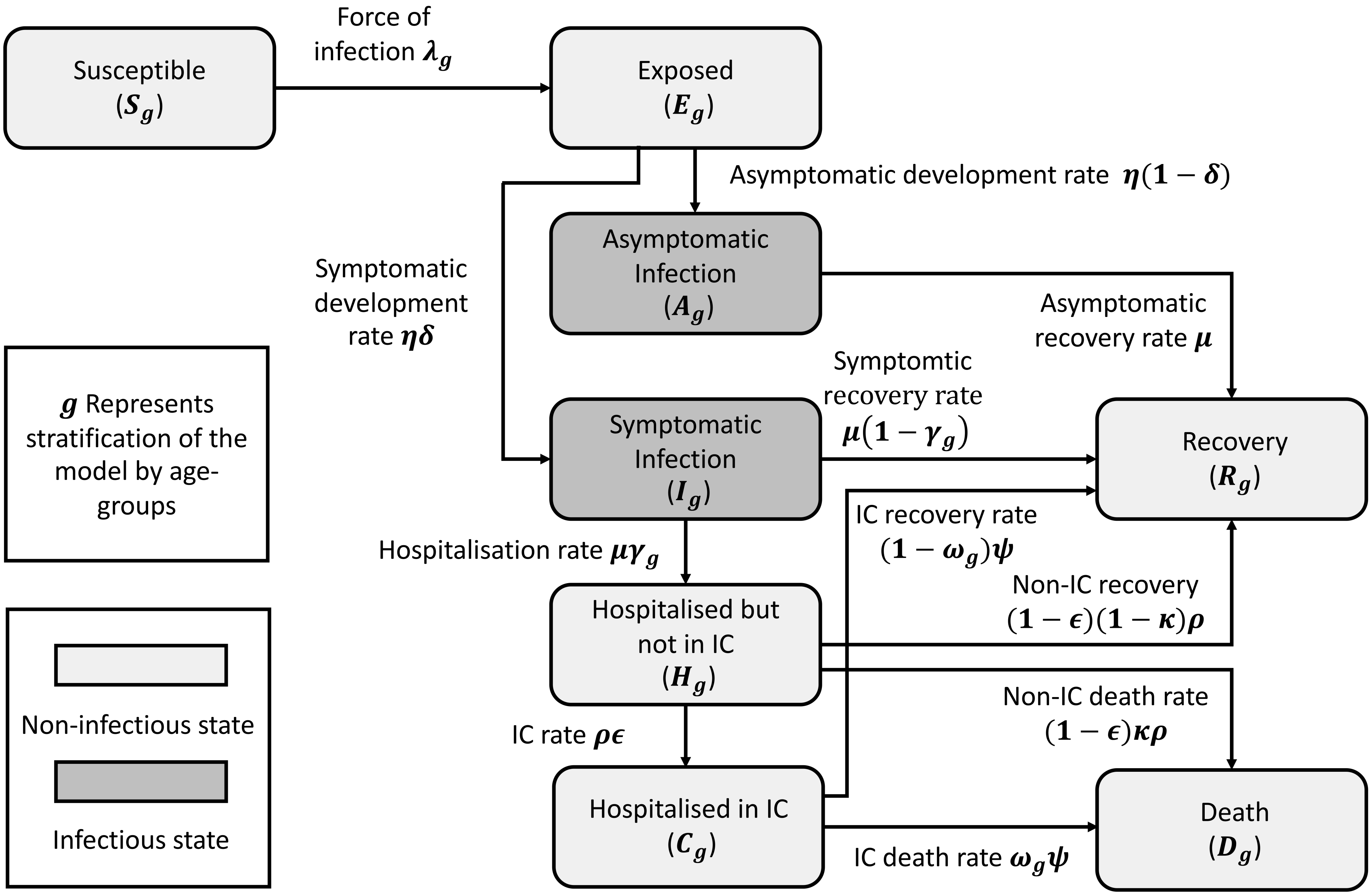
Susceptible
(S_g)
Force of infection λ_g
Exposed
(E_g)
Asymptomatic development rate $\eta(1-\delta)$
Asymptomatic Infection
(A_g)
Symptomatic development rate $\eta\delta$
Asymptomatic recovery rate μ
Symptomtic recovery rate $\mu(1-\gamma_g)$
Symptomatic Infection
(I_g)
Recovery
(R_g)
g Represents stratification of the model by age-groups
IC recovery rate $(1-\omega_g)\psi$
Hospitalisation rate $\mu\gamma_g$
Non-IC recovery $(1-\epsilon)(1-\kappa)\rho$
Hospitalised but not in IC
(H_g)
Non-infectious state
Non-IC death rate $(1-\epsilon)\kappa\rho$
IC rate $\rho\epsilon$
Hospitalised in IC
(C_g)
Death
(D_g)
Infectious state
IC death rate $\omega_g\psi$

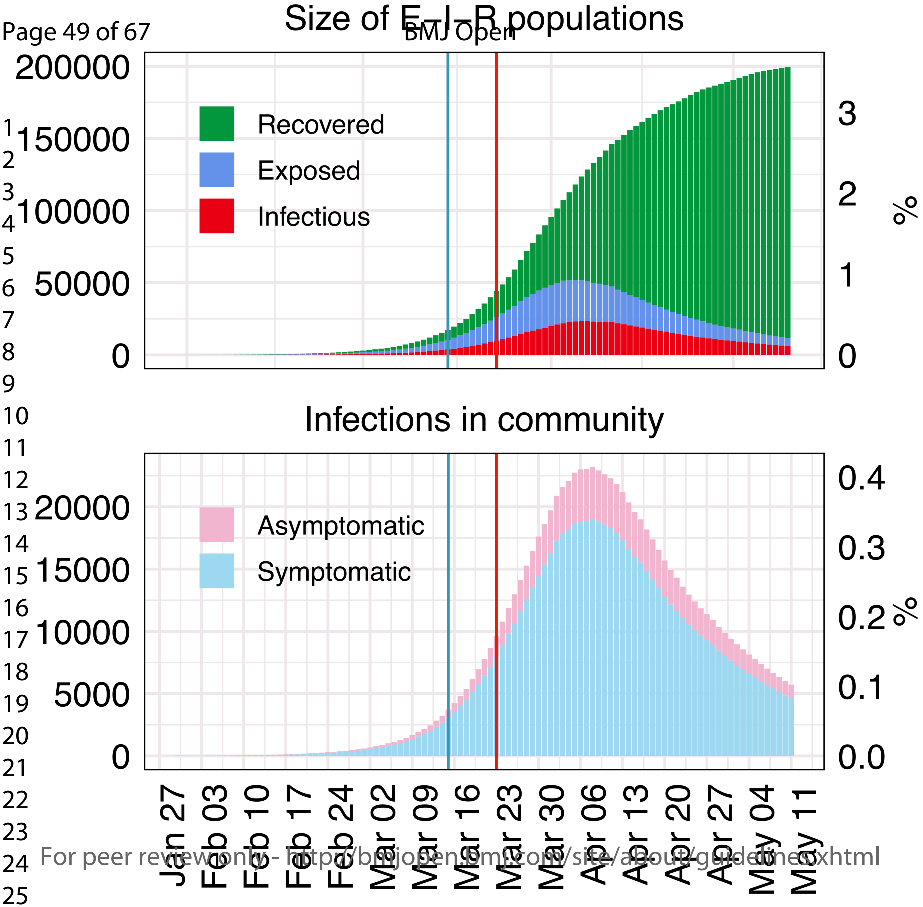

For peer review only - http://bmjopen.bmj.com/site/about/guidelines.xhtml

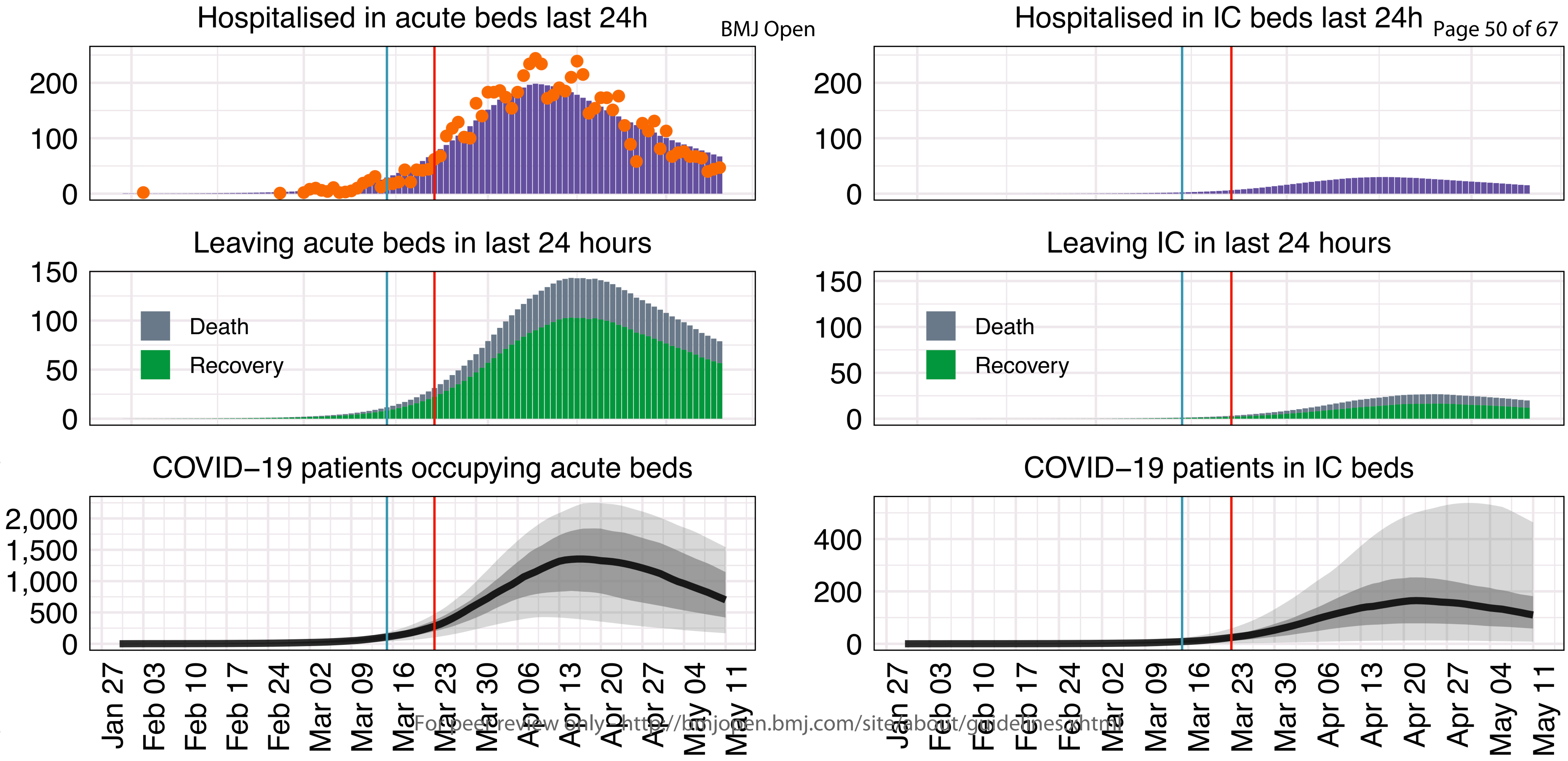

For peer review only - http://bmjopen.bmj.com/site/about/guidelines.xhtml

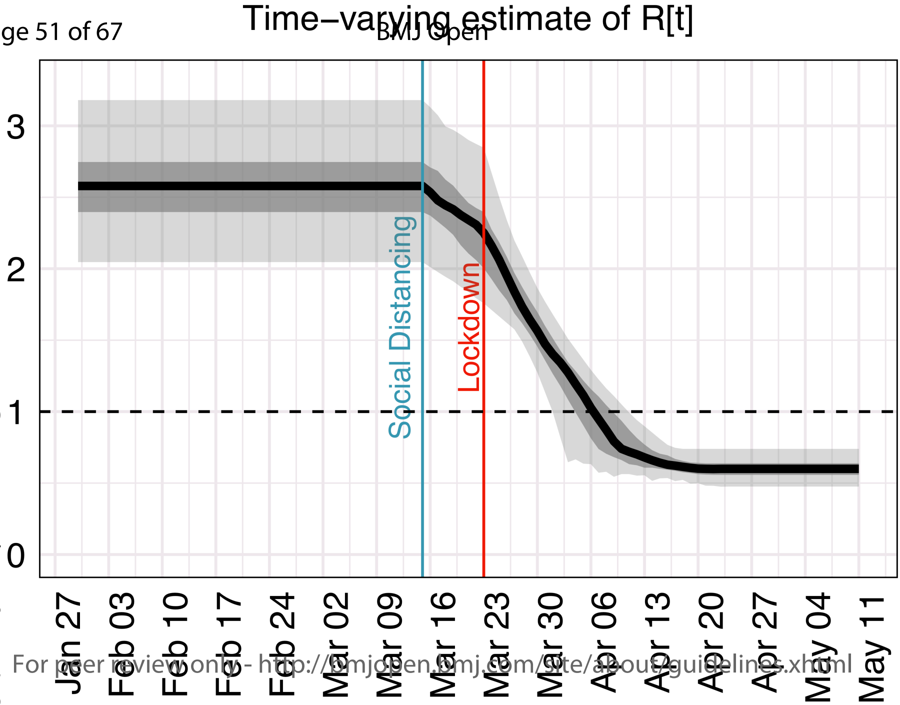

For peer review only - http://bmjopen.bmj.com/site/about/guidelines.xhtml

Supplementary information

"Estimating the COVID-19 epidemic trajectory and hospital capacity requirements in South West England: a mathematical modelling framework"

Ross D. Booton[1], Louis MacGregor[2,3], Lucy Vass[1,2], Katharine J. Looker[2,3], Catherine Hyams[4], Philip D. Bright[5], Irasha Harding[6], Rajeka Lazarus[7], Fergus Hamilton[8], Daniel Lawson[9], Leon Danon[10], Adrian Pratt[11], Richard Wood[12,13], Ellen Brooks-Pollock[1,2,3,†], Katherine M.E. Turner[1,2,3,13,†,*]

[1]*Bristol Veterinary School, University of Bristol, Bristol, UK*

[2]*Population Health Science Institute, Bristol Medical School, University of Bristol, Bristol, UK*

[3]*NIHR Health Protection Research Unit in Behavioural Science and Evaluation*

[4]*Academic Respiratory Unit, Southmead Hospital, University of Bristol, Bristol, UK*

[5]*Immunology, Pathology Sciences, North Bristol NHS Trust, Bristol, UK*

[6]*Consultant in Microbiology, University Hospitals Bristol, Bristol, UK*

[7]*Consultant in Microbiology and Infectious Diseases, University Hospitals Bristol, Bristol, UK*

[8]*Infection Science, Southmead Hospital, North Bristol NHS Trust, Bristol, UK*

[9]*School of Mathematics, University of Bristol, Bristol, UK*

[10]*College of Engineering, Mathematics and Physical Sciences, University of Exeter, Exeter, UK*

[11]*Principal BI Analyst Modelling and Analytics, NHS Bristol, North Somerset and South Gloucestershire CCG*

[12]*Head of Modelling and Analytics, NHS Bristol, North Somerset and South Gloucestershire CCG*

[13]*Health Data Research UK South-West of England Partnership*

[†]*should be considered joint senior author*

**Correspondence: katy.turner@bristol.ac.uk*

For peer review only

Age group	Population size SW England	Percent population size SW England	Percent population size UK
0-4	296,357	5.3%	6.0%
5-18	805,965	14.4%	15.4%
18-29	806,885	14.4%	15.4%
30-39	654,469	11.7%	13.4%
40-49	684,872	12.2%	12.8%
50-59	782,317	14.0%	13.4%
60-69	671,294	12.0%	10.5%
70+	897,576	16.0%	13.1%

Table S1: Demography of SW England compared to the UK.

For peer review only

Key assumptions used in the model

i. Closed, static population size with no immigration or emigration due to the model being run over a short period of time, and current travel restrictions which should prevent significant movement of individuals in and out of the South West.

ii. No nosocomial transmission.

iii. Recovered individuals are not susceptible to reinfection within the timeframe of the model horizon.

iv. Trajectory of model outputs is assuming that there is no easing of lockdown situation over the timeframe of the model horizon.

v. There is no restriction on number of hospital (IC and acute) beds available.

vi. Asymptomatic and symptomatic are equally infectious and recover at the same rate.

vii. 95% reduction in 0-18-year old contacts from school closures

viii. Range of 0 - 50% reduction in contacts from social distancing and range of 63 - 90% reduction in contact rates due to lockdown

ix. The reduction of any given contact rate is taken to be the minimum from lockdown, school closures and social distancing.

x. R_0 is sampled between 2.79 +/- 1.16 and the infectious period and transmission probability are chosen to achieve this

xi. Proportion symptomatic which require hospitalisation depends on age, with increased risk for older age groups

xii. Proportion in IC who will die depends on age, with increased risk for older age groups

xiii. The percentage of infections which become symptomatic is 73.15 - 91.05%

xiv. The percentage of those requiring hospital who will require IC 0 - 30%

xv. The percentage of those requiring acute beds (but not IC) who will die 5 - 35%

For peer review only

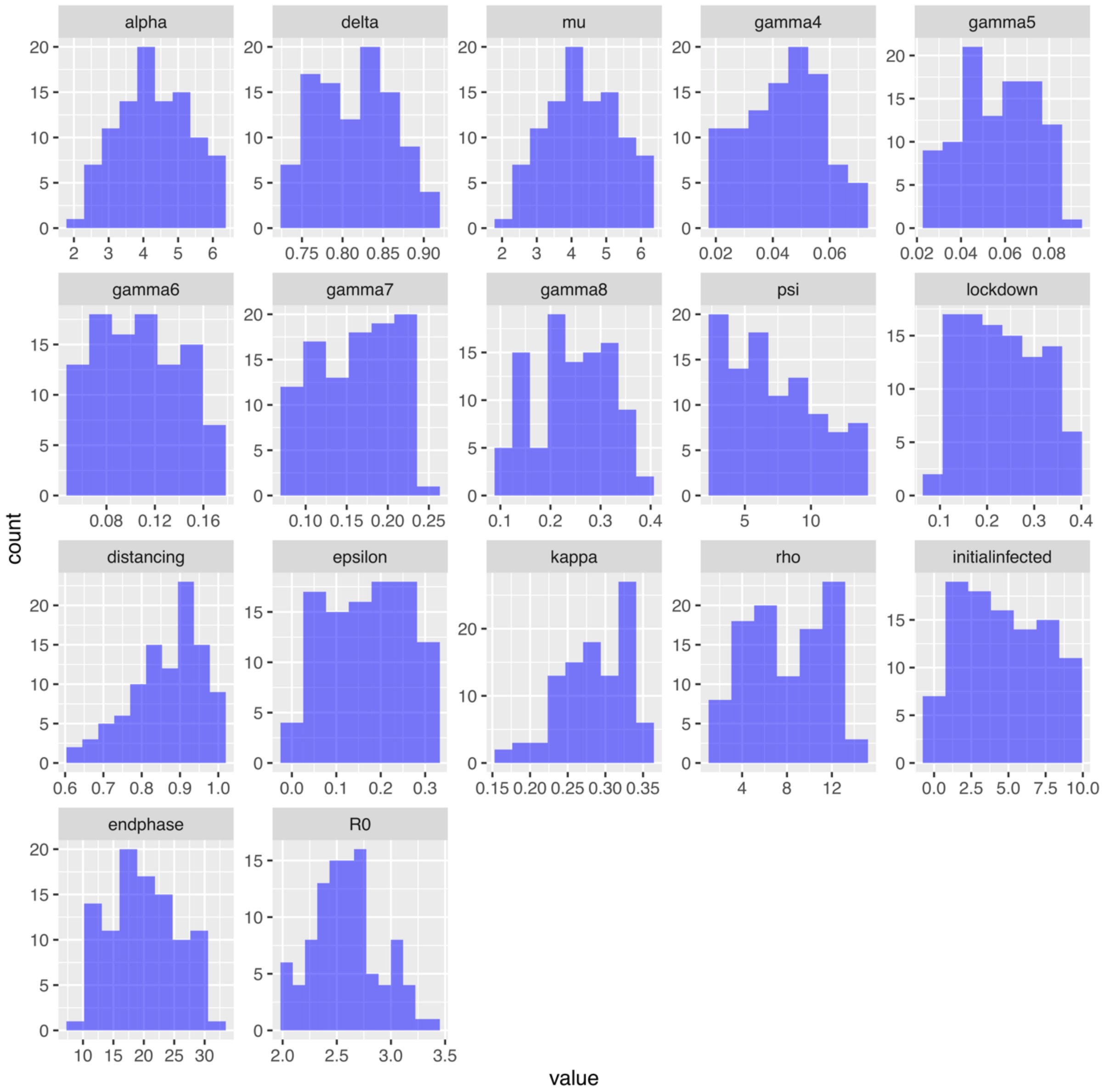

Figure S1a: The distribution of the best 100 parameters selected for in the Latin Hypercube Sampling and likelihood fitting from 100,000 simulations.

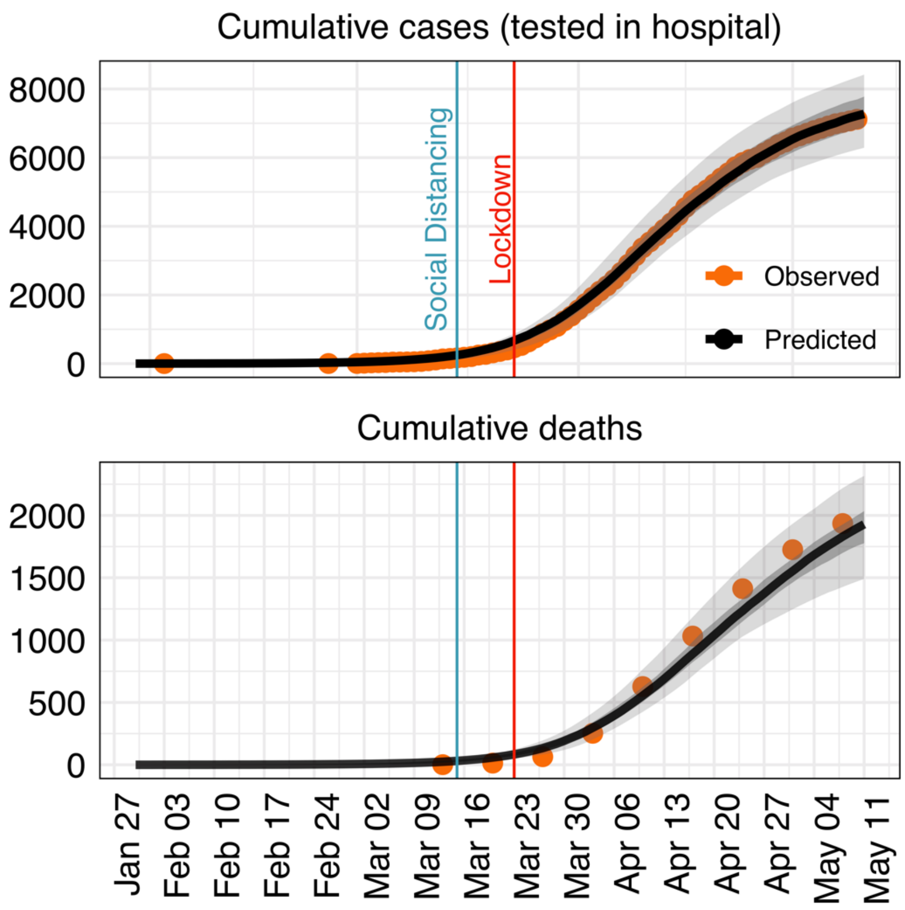

Figure S2a: Fitting performance of the model. The cumulative case numbers and hospital deaths for COVID-19 in SW England until lockdown measures were gradually lifted (11th May 2020), based on case data and death data (orange). 95% credible intervals of our model projections are shown in light grey, 50% in dark grey and the median value of the model is highlighted in black. The shaded region indicates the prediction of the model from the data. We did not consider the effects of lockdown being lifted, and our transmission rates are fitted to both before lockdown and after lockdown. Blue and red vertical lines represent the dates when social distancing and school closures/lockdown were introduced nationally, respectively.

For peer review only - http://bmjopen.bmj.com/site/about/guidelines.xhtml

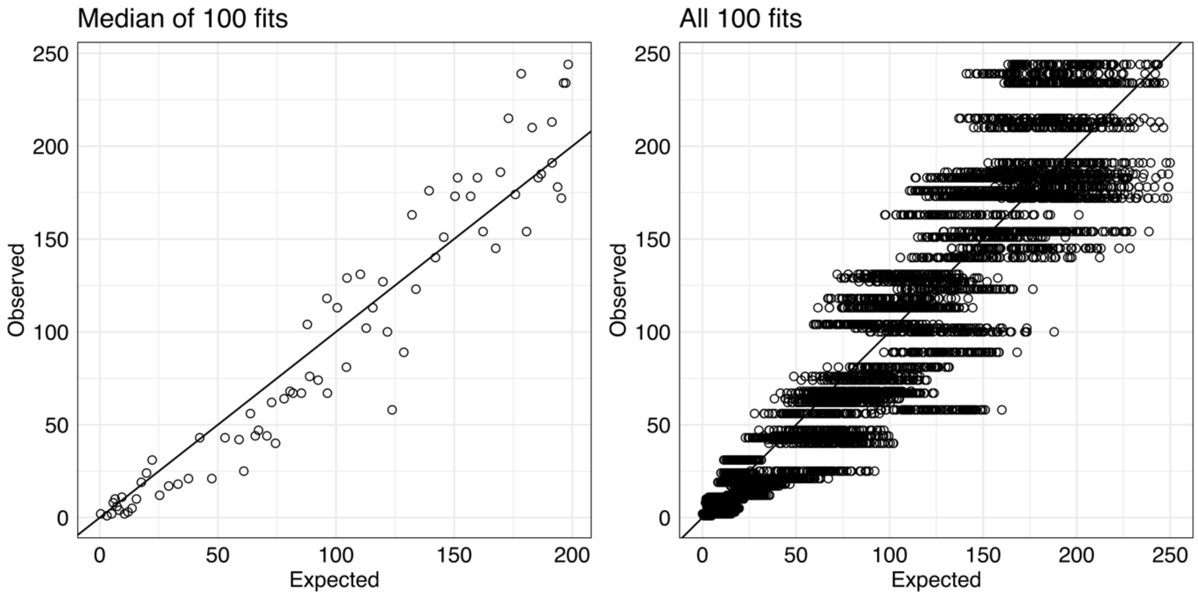

Figure S2b: The expected (model) versus observed (data) for the median of 100 fits, and all 100 fits for counts of daily case numbers. The model tends to overestimate the case data at earlier stages of the epidemic and underestimate at later stages of the epidemic.

	Cumulative Cases				Cumulative Deaths			
Date	Observed	Predicted	Predicted Lower	Predicted Upper	Observed	Predicted	Predicted Lower	Predicted Upper
30/01/2020		1	0	2		0	0	0
31/01/2020		1	0	3		0	0	0
01/02/2020		2	0	4		0	0	0
02/02/2020		2	0	4		0	0	0
03/02/2020	2	2	0	5		0	0	0
04/02/2020		3	0	6		0	0	0
05/02/2020		3	0	7		0	0	1
06/02/2020		4	0	8		0	0	1
07/02/2020		4	1	8		0	0	1
08/02/2020		4	1	9		0	0	1
09/02/2020		5	1	10		0	0	1
10/02/2020		5	1	11		1	0	2
11/02/2020		6	1	12		1	0	2
12/02/2020		6	1	13		1	0	2
13/02/2020		7	1	15		1	0	2

For peer review only

14/02/2020		8	1	16		1	0	2
15/02/2020		9	1	17		1	0	3
16/02/2020		10	2	19		1	0	3
17/02/2020		11	2	21		1	0	3
18/02/2020		12	2	23		2	0	4
19/02/2020		13	3	25		2	0	4
20/02/2020		14	3	27		2	0	4
21/02/2020		16	3	30		2	0	5
22/02/2020		18	4	33		2	1	5
23/02/2020		20	5	37		3	1	6
24/02/2020		23	5	40		3	1	6
25/02/2020		25	6	45		3	1	7
26/02/2020	3	28	7	49		4	1	8
27/02/2020		32	9	55		4	1	9
28/02/2020		36	10	61		5	1	9
29/02/2020		40	12	67		5	1	10
01/03/2020	5	45	14	75		6	2	12
02/03/2020	13	50	16	83		7	2	13
03/03/2020	23	57	19	93		8	2	14
04/03/2020	29	64	22	104		8	3	16
05/03/2020	33	72	26	116		10	3	17
06/03/2020	44	82	30	129		11	4	19
07/03/2020	46	92	35	144		12	4	21
08/03/2020	49	105	41	159		14	5	24
09/03/2020	54	118	47	177		15	6	26
10/03/2020	64	134	55	196		17	7	29
11/03/2020	83	151	65	218		19	8	32
12/03/2020	107	171	76	243		21	9	36
13/03/2020	138	194	88	271	1	24	11	40
14/03/2020	150	219	103	303		28	13	44
15/03/2020	167	248	119	339		31	15	49
16/03/2020	185	281	138	382		35	18	55
17/03/2020	206	320	161	432		40	21	62
18/03/2020	249	364	187	488		45	25	69
19/03/2020	270	410	220	551		51	28	77

For peer review only

20/03/2020	313	461	258	618	13	58	33	86
21/03/2020	355	521	301	693		65	38	97
22/03/2020	399	589	351	776		74	43	110
23/03/2020	461	669	403	868		83	50	124
24/03/2020	529	754	458	969		94	57	139
25/03/2020	633	840	519	1085		106	64	155
26/03/2020	751	946	588	1216		119	73	174
27/03/2020	880	1045	670	1354	65	134	82	195
28/03/2020	982	1154	761	1499		152	93	218
29/03/2020	1082	1271	860	1648		170	105	244
30/03/2020	1245	1406	967	1824		192	119	273
31/03/2020	1385	1552	1078	2010		216	135	305
01/04/2020	1568	1702	1201	2220		240	155	340
02/04/2020	1751	1861	1331	2435		266	177	378
03/04/2020	1937	2025	1467	2655	253	297	203	417
04/04/2020	2111	2193	1612	2881		327	231	458
05/04/2020	2265	2365	1772	3107		360	260	503
06/04/2020	2448	2552	1940	3333		394	290	550
07/04/2020	2661	2747	2121	3556		431	322	599
08/04/2020	2895	2941	2312	3782		472	356	652
09/04/2020	3139	3134	2491	4006		512	390	707
10/04/2020	3373	3327	2674	4226	628	554	424	765
11/04/2020	3545	3517	2858	4442		598	461	820
12/04/2020	3723	3710	3044	4636		641	498	876
13/04/2020	3914	3891	3227	4852		689	538	936
14/04/2020	4099	4075	3392	5065		738	578	998
15/04/2020	4309	4262	3553	5258		789	620	1061
16/04/2020	4548	4446	3710	5454		840	665	1123
17/04/2020	4763	4614	3866	5652	1032	889	714	1184
18/04/2020	4908	4779	4026	5841		938	762	1245
19/04/2020	5062	4930	4184	6021		988	807	1305
20/04/2020	5235	5090	4338	6193		1039	852	1365
21/04/2020	5408	5256	4480	6356		1089	900	1424
22/04/2020	5559	5408	4614	6510		1141	946	1481
23/04/2020	5735	5548	4746	6657		1190	989	1538

For peer review only

24/04/2020	5858	5689	4876	6796	1412	1234	1029	1593
25/04/2020	5947	5839	5001	6927		1284	1063	1647
26/04/2020	6005	5971	5122	7051		1327	1096	1699
27/04/2020	6132	6085	5239	7169		1373	1127	1749
28/04/2020	6245	6203	5353	7279		1421	1158	1797
29/04/2020	6376	6317	5450	7384		1467	1187	1844
30/04/2020	6457	6428	5539	7496		1512	1217	1889
01/05/2020	6570	6539	5624	7607	1725	1554	1247	1933
02/05/2020	6637	6638	5704	7706		1598	1276	1974
03/05/2020	6711	6722	5780	7795		1643	1304	2014
04/05/2020	6787	6801	5852	7880		1683	1331	2053
05/05/2020	6854	6869	5923	7960		1721	1357	2096
06/05/2020	6921	6947	5992	8038		1759	1383	2139
07/05/2020	6985	7010	6057	8113		1794	1403	2180
08/05/2020	7025	7090	6119	8193	1935	1830	1427	2219
09/05/2020	7069	7160	6178	8268		1864	1449	2253
10/05/2020	7116	7218	6235	8341		1897	1470	2286
11/05/2020	7172	7273	6288	8415		1928	1491	2317

81 **Table S2:** The predicted median and 95% credible interval for cumulative cases and deaths alongside
82 the observed cumulative cases and deaths.
83

For peer review only

Model performance

Here we evaluate the model performance as a tool to forecast the potential cumulative cases and deaths related to COVID-19. We do this by using only a certain percentage of the total available data until a) 27th April 2020, b) 20th April 2020, c) 13th April 2020 and d) 6th April 2020) to fit the model, then comparing these projections to the actual data observed past each of these dates. This gives an interpretable way to estimate how well the forecast generated by the model could be expected to perform.

Figures S3a-d show the overall performance of the model, which in general overestimates the total cumulative cases, but captures the cumulative deaths more accurately. All final estimates from the data are within the 95% expected credible interval predictions regardless of the date chosen to fit the model to.

Figure S3a shows the model prediction using data only up to 27th April 2020, which is based on 82% of the available data. This the most reliable prediction, while the most variable predictions come from those which use 53% of the available data (Figure S3d), suggesting that the model can accurately forecast the immediate future with higher reliability – while further time points are harder to predict (although the model predictions based on only 53% of the data also perform reasonably well and the final actuals are within the predicted 95% credible interval).

For peer review only

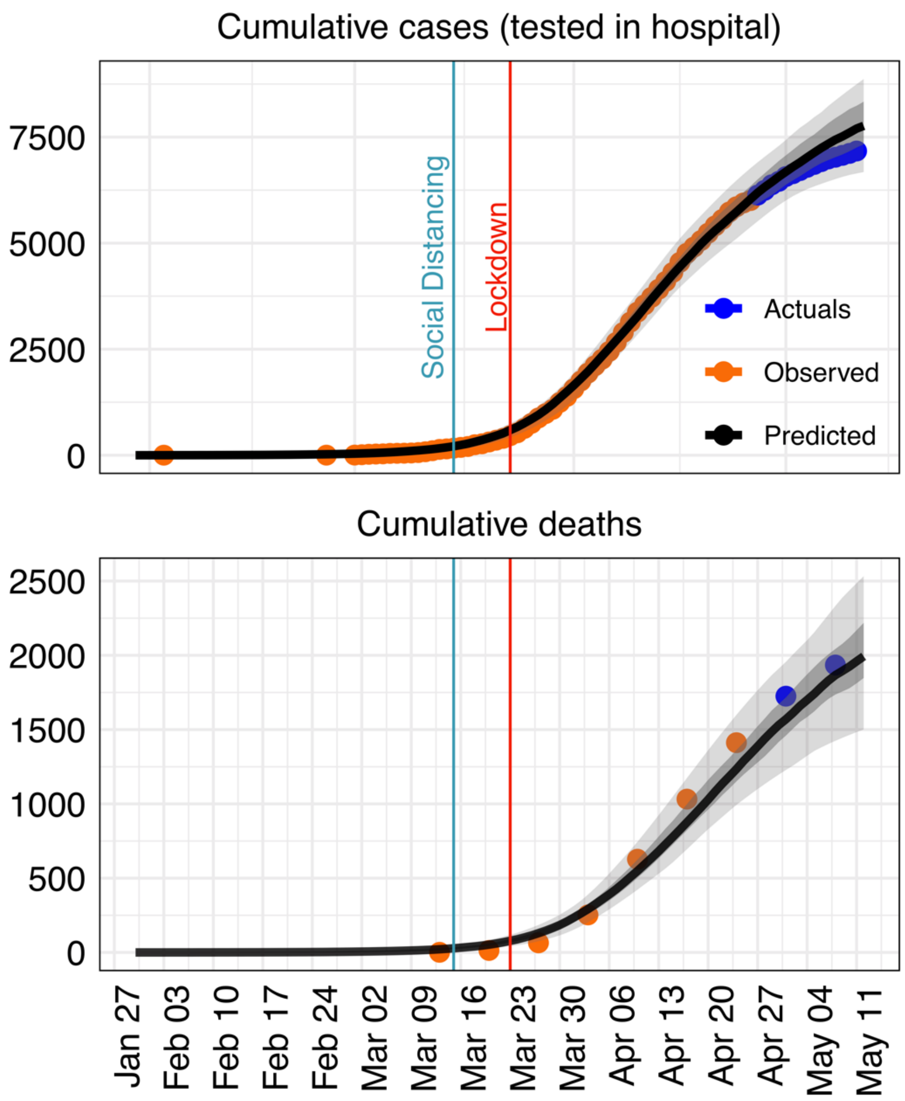

Figure S3a: Fitting performance of the model using only the data until the 27th April 2020 (which represents 82% of the available data points). 95% credible intervals of our model projections are shown in light grey, 50% in dark grey and the median value of the model is highlighted in black. The shaded region indicates the prediction of the model from the data. Blue and red vertical lines represent the dates when social distancing and school closures/lockdown were introduced nationally, respectively. ‘Actuals’ represent the points not included in the fitting, while observed data are used to fit the model.

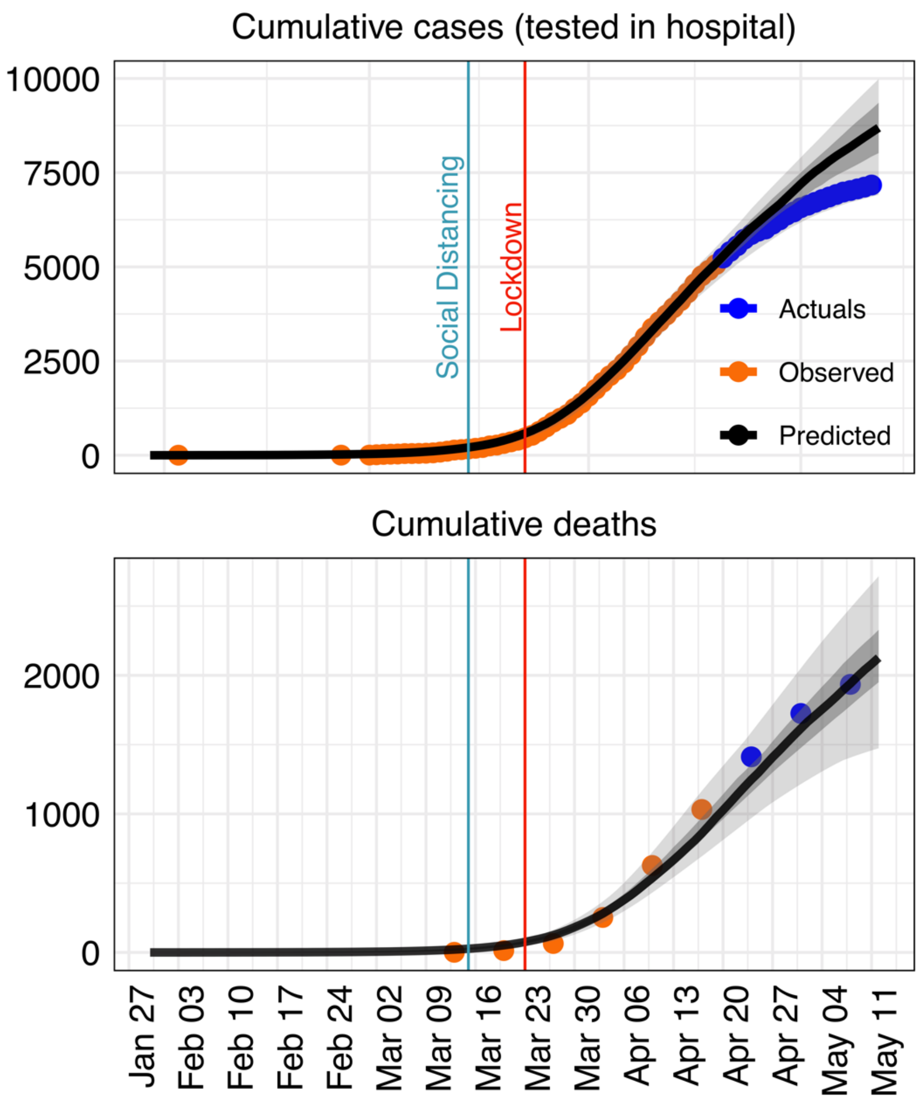

Figure S3b Fitting performance of the model using only the data until the 20th April 2020 (which represents 72% of the available data points).

only

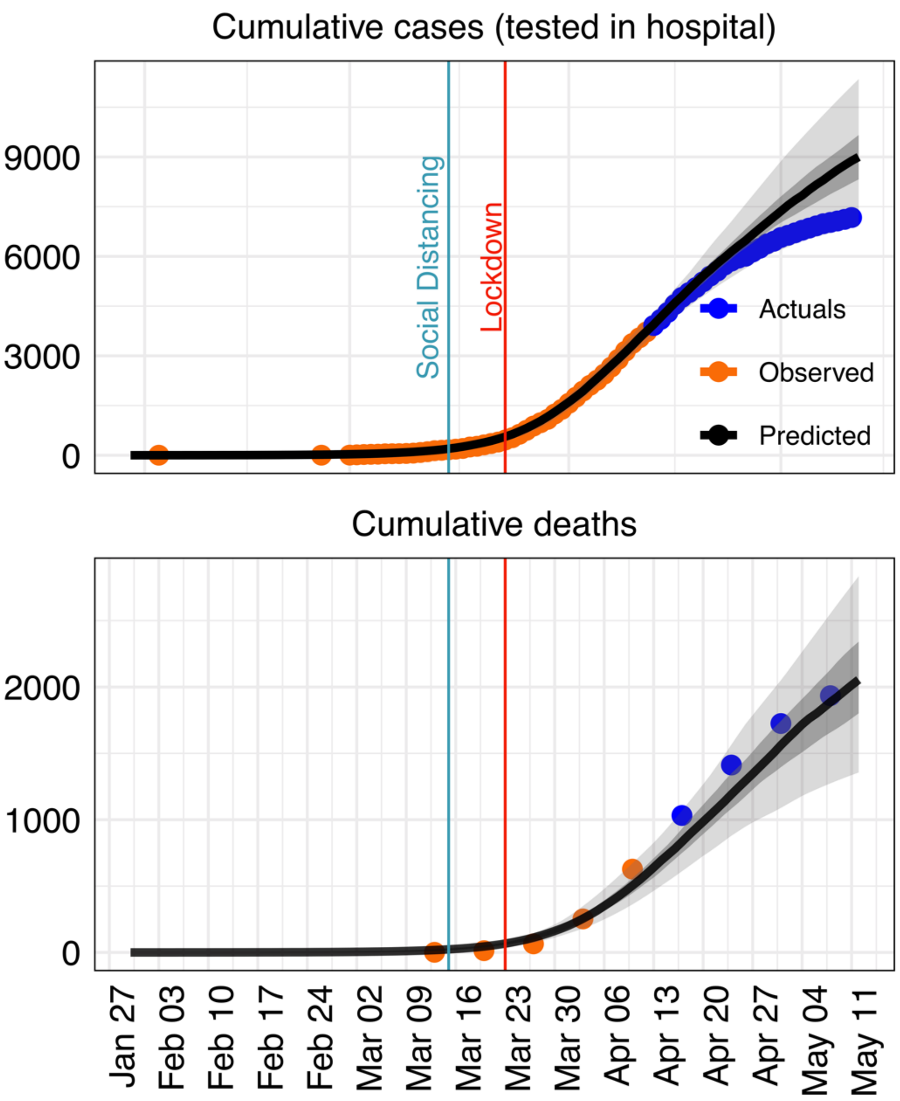

119

120 **Figure S3c:** Fitting performance of the model using only the data until the 13th April 2020 (which

121 represents 62% of the available data points).

122

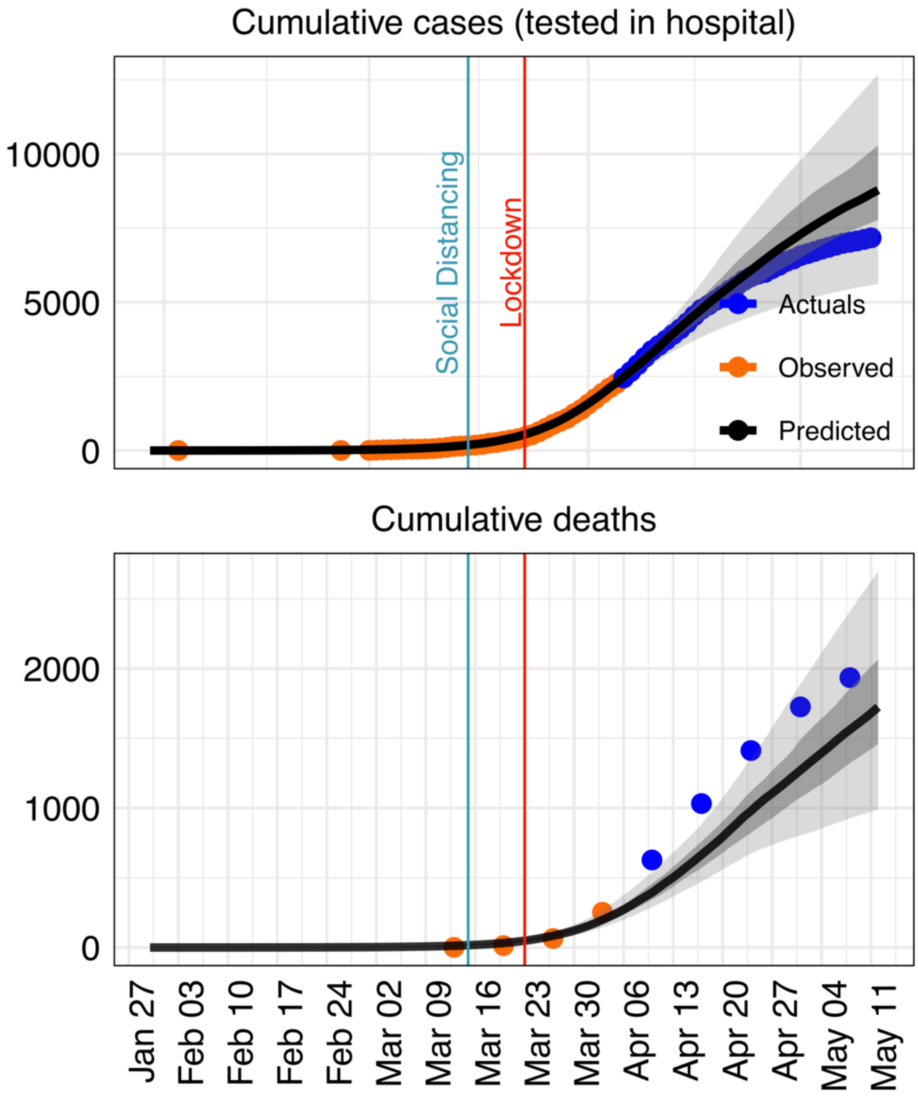

Figure S3d: Fitting performance of the model using only the data until the 6th April 2020 (which represents 53% of the available data points).

only

Sensitivity analysis

The choice of the final sample size *m*=100 taken from 100k samples is based on a bias-variance trade-off with lower m giving a better model fit but higher *m* allowing more predictions to be included. The choice of *m* is not of practical significance for the median and 95% credible intervals of the main outcome measures of the model – bed modelling - as shown in the below table.

	**Median (95% CrI) value on 11th May 2020**			
**Size of final sample size from 100k samples**	*Total recovered*	*Total infectious*	*Total in acute beds*	*Total in IC beds*
50	191k (143k – 276k)	5k (2k – 10k)	579 (152 – 1424)	107 (14 – 497)
100	189k (142k – 278k)	6k (2k – 12k)	701 (169 – 1543)	110 (8 – 464)
200	189k (141k - 276k)	6k (2k – 16k)	709 (171 – 1564)	105 (5 – 454)
500	191k (132k – 289k)	7k (2k – 20k)	690 (171 – 1644)	109 (5 – 426)

Increasing *m* does slightly increase the estimate of the infectious population (*total infectious)*. This is explained by including models with slightly higher *R* value, i.e. *R* is biased upwards as we move further away from the best answer. The bias is small compared to the modelling uncertainty.

	**Median (95% CrI) of parameter**							
**Size of final sample size from 100k samples**	*R*	δ	$1/\mu$	$1/\rho$	$1/\psi$	ϵ	κ	*endphase*
50	2.5 (2.1 - 3.1)	82 (74 – 90%)	3.9 (2.6 – 5.8)	6.3 (2.4 – 13.1)	6.5 (2.6 – 13.8)	19 (4 – 28%)	29 (19 – 34%)	21.3 (11.1 – 30.0)
100	2.6 (2.1 - 3.2)	82 (74 – 90%)	4.3 (2.5 – 6.0)	7.7 (2.3 – 13.3)	6.7 (2.5 – 13.8)	16 (2 – 30%)	28 (19 – 34%	19.7 (10.8 – 30.0)
200	2.6 (2.1 - 3.3)	82 (74 – 91%)	4.5 (2.4 – 6.7)	7.8 (2.3 – 13.6)	6.7 (2.4 – 13.8)	16 (1 – 30%)	28 (17 – 34%)	18.5 (8.6 – 30.3)
500	2.7 (2.1 - 3.5)	82 (74 – 90%)	5.0 (2.5 – 8.3)	7.3 (2.4 – 13.6)	7.2 (2.5 – 13.8)	17 (1 – 29%)	28 (15 – 35%)	18.3 (6.4 – 30.3)

For peer review only

139 The inverse of μ and ψ becomes bigger (as it is related to the selection of R), as the sample size becomes larger. This then reduces the parameters *endphase*, κ, and ϵ in order to account for this bias in R.

143 We choose to report values for *m*=100 samples as part of the bias-variance trade-off. The bias is small for this choice, with R close to unbiased and the confidence intervals capture all of the significant variation components. The qualitative inferences would not change with any of the above choices of *m* and uncertainty has been well captured.

For peer review only

TRIPOD Checklist: Prediction Model Development

Section/Topic	Item	Checklist Item	Page
**Title and abstract**			
Title	1	Identify the study as developing and/or validating a multivariable prediction model, the target population, and the outcome to be predicted.	**1**
Abstract	2	Provide a summary of objectives, study design, setting, participants, sample size, predictors, outcome, statistical analysis, results, and conclusions.	**2**
**Introduction**			
Background and objectives	3a	Explain the medical context (including whether diagnostic or prognostic) and rationale for developing or validating the multivariable prediction model, including references to existing models.	**3**
	3b	Specify the objectives, including whether the study describes the development or validation of the model or both.	**3, 4**
**Methods**			
Source of data	4a	Describe the study design or source of data (e.g., randomized trial, cohort, or registry data), separately for the development and validation data sets, if applicable.	**11, 12, 13**
	4b	Specify the key study dates, including start of accrual; end of accrual; and, if applicable, end of follow-up.	**14**
Participants	5a	Specify key elements of the study setting (e.g., primary care, secondary care, general population) including number and location of centres.	**14**
	5b	Describe eligibility criteria for participants.	**n/a**
	5c	Give details of treatments received, if relevant.	**n/a**
Outcome	6a	Clearly define the outcome that is predicted by the prediction model, including how and when assessed.	**14**
	6b	Report any actions to blind assessment of the outcome to be predicted.	**n/a**
Predictors	7a	Clearly define all predictors used in developing or validating the multivariable prediction model, including how and when they were measured.	**12, 13, 14**
	7b	Report any actions to blind assessment of predictors for the outcome and other predictors.	**n/a**
Sample size	8	Explain how the study size was arrived at.	**12**
Missing data	9	Describe how missing data were handled (e.g., complete-case analysis, single imputation, multiple imputation) with details of any imputation method.	**12**
Statistical analysis methods	10a	Describe how predictors were handled in the analyses.	**12**
	10b	Specify type of model, all model-building procedures (including any predictor selection), and method for internal validation.	**6 - 9**
	10d	Specify all measures used to assess model performance and, if relevant, to compare multiple models.	**12, 14**
Risk groups	11	Provide details on how risk groups were created, if done.	**6**
**Results**			
Participants	13a	Describe the flow of participants through the study, including the number of participants with and without the outcome and, if applicable, a summary of the follow-up time. A diagram may be helpful.	**9**
	13b	Describe the characteristics of the participants (basic demographics, clinical features, available predictors), including the number of participants with missing data for predictors and outcome.	**6, 7**
Model development	14a	Specify the number of participants and outcome events in each analysis.	**12**
	14b	If done, report the unadjusted association between each candidate predictor and outcome.	**n/a**
Model specification	15a	Present the full prediction model to allow predictions for individuals (i.e., all regression coefficients, and model intercept or baseline survival at a given time point).	**14 - 17**
	15b	Explain how to the use the prediction model.	**6 - 9**
Model performance	16	Report performance measures (with CIs) for the prediction model.	**14 - 17**
**Discussion**			
Limitations	18	Discuss any limitations of the study (such as nonrepresentative sample, few events per predictor, missing data).	**18 - 23**
Interpretation	19b	Give an overall interpretation of the results, considering objectives, limitations, and results from similar studies, and other relevant evidence.	**18 - 23**
Implications	20	Discuss the potential clinical use of the model and implications for future research.	**23**
**Other information**			
Supplementary information	21	Provide information about the availability of supplementary resources, such as study protocol, Web calculator, and data sets.	**33 - 40**
Funding	22	Give the source of funding and the role of the funders for the present study.	**23, 24**

We recommend using the TRIPOD Checklist in conjunction with the TRIPOD Explanation and Elaboration document.

For peer review only - http://bmjopen.bmj.com/site/about/guidelines.xhtml
